# Indolizines and pyrrolo[1,2-*c*]pyrimidines decorated with a pyrimidine and a pyridine unit respectively

**DOI:** 10.3762/bjoc.11.121

**Published:** 2015-06-26

**Authors:** Marcel Mirel Popa, Emilian Georgescu, Mino R Caira, Florentina Georgescu, Constantin Draghici, Raluca Stan, Calin Deleanu, Florea Dumitrascu

**Affiliations:** 1 Center for Organic Chemistry C.D. Nenitzescu, Romanian Academy, Spl. Independentei 202B, Bucharest 060023, Romania; 2 Faculty of Applied Chemistry and Materials Science, ‘Politehnica’ University of Bucharest, Polizu Street 1-7, 011061 Bucharest, Romania; 3 Research Center Oltchim, Str. Uzinei 1, RO 240050, Ramnicu Valcea, Romania; 4 Department of Chemistry, University of Cape Town, Rondebosch 7701, South Africa; 5 Research Dept., Teso Spec SRL, Str. Muncii 53, RO-915200 Fundulea, Calarasi, Romania

**Keywords:** indolizine, nitrogen heterocycles, *N*-ylide, 4-pyridylpyrimidine, pyrrolo[1,2-*c*]pyrimidine

## Abstract

The three possible structural isomers of 4-(pyridyl)pyrimidine were employed for the synthesis of new pyrrolo[1,2-*c*]pyrimidines and new indolizines, by 1,3-dipolar cycloaddition reaction of their corresponding *N*-ylides generated in situ from their corresponding cycloimmonium bromides. In the case of 4-(3-pyridyl)pyrimidine and 4-(4-pyridyl)pyrimidine the quaternization reactions occur as expected at the pyridine nitrogen atom leading to pyridinium bromides and consequently to new indolizines via the corresponding pyridinium *N*-ylides. However, in the case of 4-(2-pyridyl)pyrimidine the steric hindrance directs the reaction to the pyrimidinium *N*-ylides and, subsequently, to the formation of the pyrrolo[1,2-*c*]pyrimidines. The new pyrrolo[1,2-*c*]pyrimidines and the new indolizines were structurally characterized through NMR spectroscopy. The X-ray structures of two of the starting materials, 4-(2-pyridyl)pyrimidine and 4-(4-pyridyl)pyrimidine, are also reported.

## Introduction

Two heteroarenes linked through a single bond [1] proved to be versatile structural motifs for a broad range of compounds with applications as advanced fluorescent materials [2–3], ligands [4–5], bioactive compounds [6], and for the design of dynamic chemical devices [7–10]. The most accessible route to hybrid heteroarenes is the direct coupling [1,11–12]. However, in some cases alternative routes could be useful to achieve structural variety [13]. An alternative route is to start from accessible simple heteroarenes linked by a single bond and to transform one of them or both into more complex structures [14–15].

**Figure 1:**
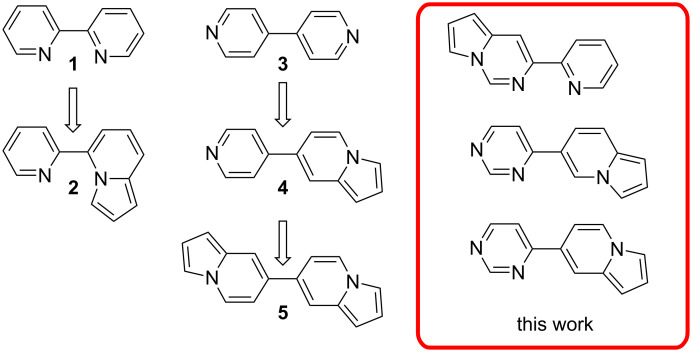
Examples of hybrid heteroarenes from the class of indolizines.

Nitrogen-containing compounds from the class of indolizines and azaindolizines are known as fluorescent materials [2–4,15] with applications as chemosensors [16] and as bioactive compounds [17–20]. For example, starting from bispyridyl derivatives such as **1** and **3**, fluorescent compounds from the class of indolizines, as for example compounds **2**, **4** and **5** (Figure 1) were obtained [14–15].

Based on our previous experience [21–25] we investigated the synthesis and structural characterization of new pyrrolo[1,2-*c*]pyrimidines that are 3-substituted with a pyridine unit and new indolizines substituted in positions 6 or 7 with a pyrimidine. Herein, we report the efficient synthesis of small libraries of structurally diverse pyrrolo-fused heterocycles starting from the 4-pyridylpyrimidine isomers via 1,3-dipolar cycloaddition of corresponding *N*-ylides with several activated alkynes. The biologic and luminescent properties of newly synthesized 3-pyridylpyrrolo[1,2-*c*]pyrimidine and 7-pyrimidinylindolizine derivatives are under investigation.

## Results and Discussion

**Figure 2:**
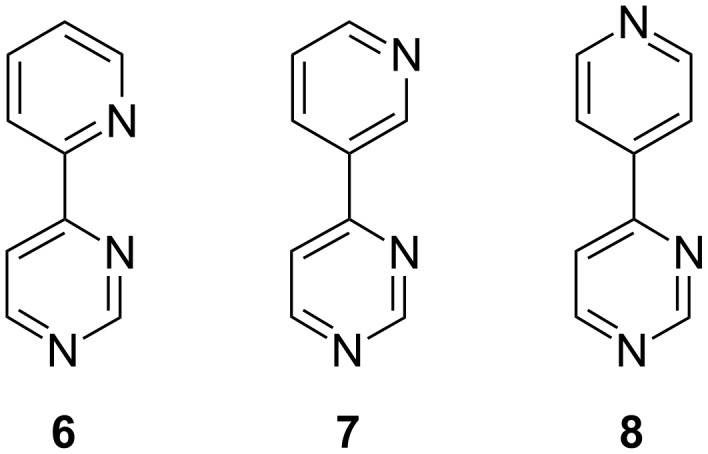
Starting 4-pyridylpyrimidines **6**–**8**.

Our purpose was to obtain new hetarylpyridines and hetarylpyrimidines. Thus, starting from the 4-pyridylpyrimidine isomers **6**–**8** (Figure 2) we have obtained new pyrrolo[1,2-*c*]pyrimidines or pyrimidinyl-substituted indolizines. The synthetic strategy was the 1,3-dipolar cycloaddition reaction of the corresponding *N*-ylides [21–25]. The structural variety of the compounds was determined by the series of dipolarophiles employed. The new final compounds were structurally characterized and our preliminary investigations suggest that some of them possess relevant degrees of fluorescence.

For the synthesis of 4-pyridylpyrimidines **6**–**8** there are some synthetic methods reported in the literature [26–29]. 4-(2-Pyridyl)pyrimidine (**6**) was obtained according to Bejan et al. [28] in 44% yield while the reaction for obtaining 4-(3-pyridyl)pyrimidine did not work. However, we devised an appropriate method for the synthesis of 4-pyridylpyrimidines, analogous to that reported for the synthesis of 4-phenylpyrimidines [30]. Thus, by the reaction of trisformylaminomethane and corresponding substituted acetylpyridines in the presence of *p*-toluenesulfonic acid the 4-pyridylpyrimidines were obtained in 20–25% yields. In the case of 4-(4-pyridyl)pyrimidine (**8**) the reaction was carried out starting from trisformylaminomethane and the 4-acetylpyridine hydrochloride, the reaction yield increasing to 41%. The compounds were crystallized from methylcyclohexane resulting in waxy crystals of 4-(2-pyridyl)-pyrimidine with mp 75–78 °C (lit: [28], 77–80 °C), 4-(3-pyridyl)pyrimidine with mp 85–88 °C (lit: [26], 89 °C) and 4-(4-pyridyl)pyrimidine with mp 131–133 °C (lit: [29], 132–133 °C).

The chemistry of 4-(2-pyridyl)pyrimidine (**6**) is rather scarce compared to its structural 2,2’-bipyridyl analogue but should raise interest for similar synthetic applicative purposes. Also the isomers of **6**, compounds **7** and **8**, are of interest for coordination chemistry both as ligands and as bridging compounds. For this reason we paid some attention to their structural characterization. To our knowledge the X-ray structures for the parent compounds **6**–**8** were not reported previously and we thus attempted to obtain suitable single crystals. Only compounds **6** and **8** could be isolated as monocrystals and their X-ray structures were determined. Disordered models were necessarily invoked to account for the requirement of molecular centrosymmetry imposed by their respective crystallographic space groups. The detailed description of the X-ray structures is given below.

Having at our disposal 4-(2-pyridyl)pyrimidine (**6**), 4-(3-pyridyl)pyrimidine (**7**), and 4-(4-pyridyl)pyrimidine (**8**) as starting materials we managed to obtain new pyrrolo-fused derivatives by 1,3-dipolar cycloaddition reactions of their corresponding *N*-ylides in 1,2-epoxybutane as reaction medium and deprotonating agent.

### Pyrrolo[1,2-*c*]pyrimidines from 4-(2-pyridyl)pyrimidine (**6**)

**Scheme 1:**
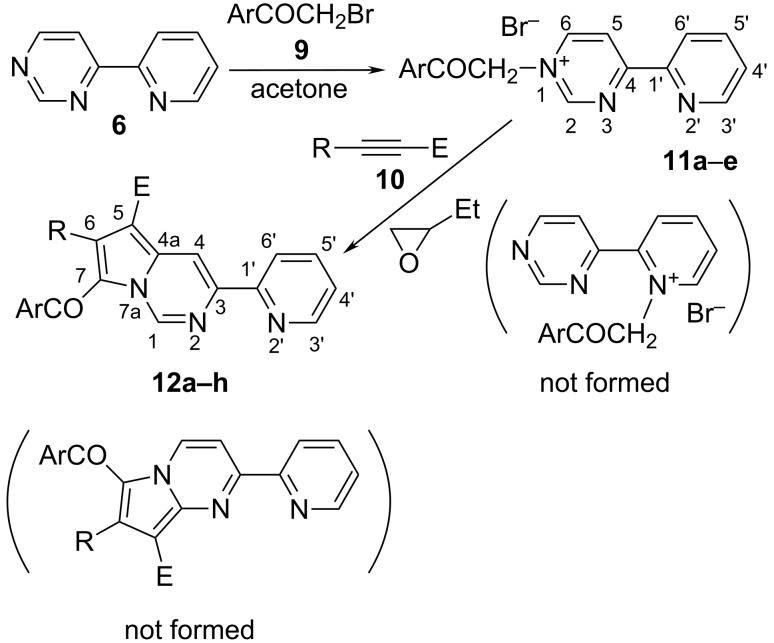
The synthesis of new pyrimidinium bromides **11** and pyrrolo[1,2-*c*]pyrimidines **12**.

Counterintuitively, starting from **6,** we obtained new pyrrolo[1,2-*c*]pyrimidines **12** instead of the expected indolizines. The nitrogen atom of the pyridine moiety is more reactive and it is expected that the quaternization reaction takes place preferentially at that atom, rather than at one of the nitrogen atoms in the pyrimidine. However due to steric hindrance, in the case of **6** the quaternization reaction takes place at the N1 nitrogen of the pyrimidine leading to the corresponding pyrimidinium bromides **11** (Scheme 1). The compounds **11** and **12** are presented in Table 1.

**Table 1:** The bromides **11** and the new pyrrolo[1,2-*c*]pyrimidines **12**.

entry	Ar	E	R	yield	mp (°C)

**11a**	C_6_H_5_	—	—	80	227–229
**11b**	4-ClC_6_H_4_	—	—	87	222–223
**11c**	4-BrC_6_H_4_	—	—	90	242–243
**11d**	3-NO_2_C_6_H_4_	—	—	80	219–221
**11e**	4-NO_2_C_6_H_4_	—	—	78	226–228
**12a**	4-BrC_6_H_4_	COMe	H	41	263–265
**12b**	3-NO_2_C_6_H_4_	COMe	H	46	255–257
**12c**	C_6_H_5_	CO_2_Me	H	52	218–219
**12d**	4-BrC_6_H_4_	CO_2_Me	H	62	234–236
**12e**	3-NO_2_C_6_H_4_	CO_2_Me	H	54	233–235
**12f**	4-ClC_6_H_4_	CO_2_Et	H	40	204–205
**12g**	4-NO_2_C_6_H_4_	CO_2_Et	H	48	218–220
**12h**	3-NO_2_C_6_H_4_	CO_2_Me	CO_2_Me	45	246–248

**Figure 3:**
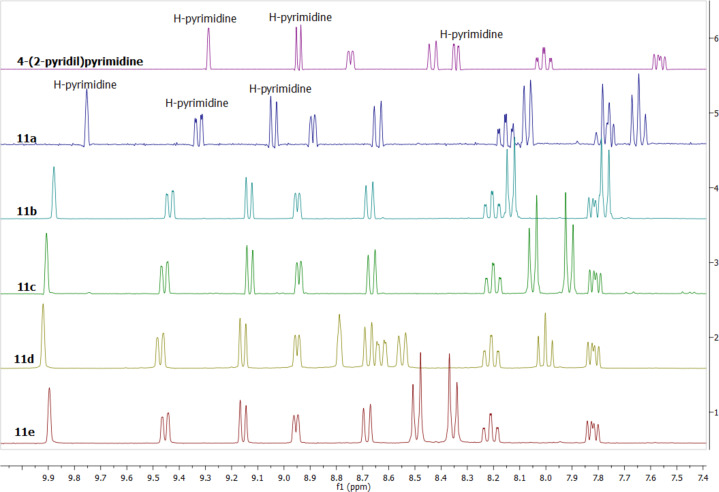
The ^1^H NMR spectra (DMSO-*d*_6_) of 4-(2-pyridyl)pyrimidine (**6**) and the corresponding bromides **11a–e** (the aromatic region).

No quaternization product at the N3 or at the nitrogen atom of the pyridine is observed. The new bromides **11** were obtained in good yields in acetone at room temperature by reacting **6** with different bromoacetophenones, and they were characterized by NMR spectroscopy which confirmed their structure. The ^1^H NMR spectra of the bromides **11** confirm that the quaternization reaction took place at the N1 of the pyrimidine ring. This was confirmed by the deshielding effect of the quaternization at the N1 atom of the pyrimidine upon the atoms H-2, H-5 and H-6 (Figure 3).

The three sets of signals in the pyrimidine moiety appear as expected, thus H-5 and H-6 appear as two doublet of doublets (*J* = 6.6, 0.8, and *J* = 6.6, 1.7 Hz, respectively; H-5 may appear as a doublet due to the small value of the coupling constant with H-2). The hydrogen H-2 is the most deshielded and appears at 9.86–9.92 ppm (compared to 9.30 ppm in compound **6**, Figure 3) as a broad singlet due to the coupling with H-5 and H-6. The atoms in the pyridine moiety appear as four sets of signals with the expected multiplicities (they were assigned by COSY and HETCOR experiments). The methylene protons appear in the range 6.43–6.67 ppm as a sharp singlet for all the compounds **11a**–**e**. The ^13^C NMR spectra of **11a**–**e** present all the expected signals. The C=O is observed at about 190 ppm. The most deshielded tertiary carbon atoms are C-2, C-6 and C-3’, which are directly bonded to nitrogen atoms. The aliphatic carbon atom appears at 62.8–63.2 ppm. All the signals in the benzoyl moieties appear as expected.

The pyrimidinium bromides **11** were allowed to react with different activated alkynes in 1,2-epoxybutane under reflux with stirring for 24 h, to obtain the new pyrrolo[1,2-*c*]pyrimidines **12** decorated with a pyridyl moiety. No trace of the corresponding pyrrolo[1,2-*a*]pyrimidine was observed as previously reported by us [31–32]. The yields were moderate to good.

The NMR data of compounds **12** show strong evidence for the formation of the new compounds. The main ^1^H NMR features are the signals in the pyrrolo[1,2-*c*]pyrimidine which were assigned by COSY and HETCOR 2D experiments. Thus, H-6 appears as a singlet at around 7.83–7.91 ppm confirming the regioselectivity of the cycloaddition reaction in the case of compounds **12a–g**. The atoms H-1 and H-4 appear as two doublets with *J* = 1.4 Hz (1.6 Hz in CDCl_3_ with TFA added). The signals in the pyridine moiety and benzoyl moiety appear as expected. The ^13^C NMR exhibits all the carbon atom signals as expected. The CO signals appear in accordance with the nature of the substituents. In the case when acetyl is replaced by a carbomethoxy or a carboethoxy group C-5 is shielded from around 118 ppm (**12a,b**) to around 108 ppm (**12c–h**). The carbon C-1 is the most deshielded in all the series of compounds due to its direct bond to two nitrogen atoms.

### New indolizines starting from 4-(3-pyridyl)pyrimidine and 4-(4-pyridyl)pyrimidine

In the case of **7** and **8** the reaction proceeds as expected, the quaternization reactions taking place at the nitrogen atom in the pyridine moiety compared to 4-(2-pyridyl)pyrimidine (**6**). The reactions were carried out via a multicomponent approach by mixing all the starting materials in 1,2-epoxybutane under reflux. In some cases the isolation and characterization of the intermediate bromide salts was performed and will be discussed in each case.

**Scheme 2:**
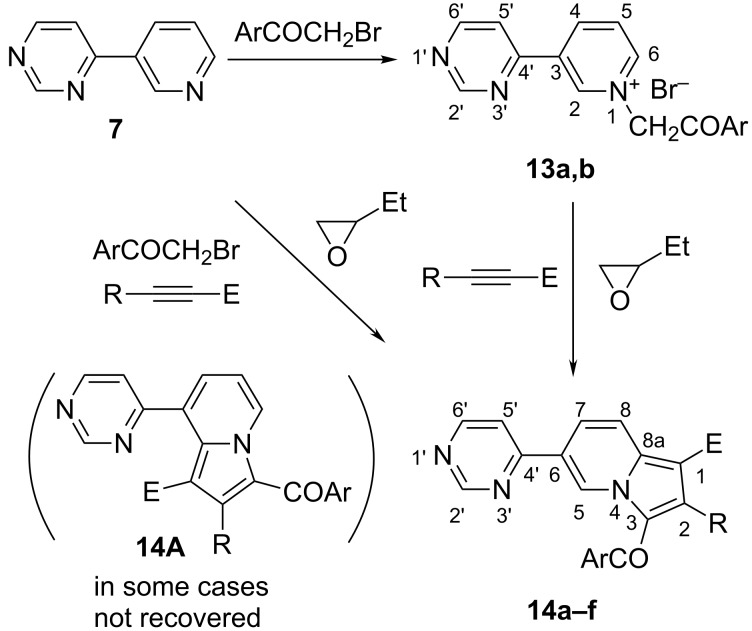
The synthesis of new pyridinium bromides **13a,b** and indolizines **14a–f**.

In the case of pyridinium ylides generated from 4-(3-pyridyl)pyrimidine (**7**), new indolizines **14a**–**f** decorated with a pyrimidine moiety were obtained (Scheme 2). The reaction appears to be selective towards the indolizines **14**. In some cases the other possible indolizine (**14A**) was observed also in the bulk reaction mass in low to medium quantities but was not recovered, further work being in progress to investigate this aspect. Due to the fact that the salts **13** are not easy to work up we ran the reaction in a multicomponent approach and only in two cases, **13a,b**, we separated the salts and assigned their structures by NMR spectroscopy. The ^1^H NMR spectra of the bromides **13a** and **13b** show that the quaternization reaction took place at the nitrogen atom of the pyridine. This was concluded by assigning all the protons by COSY and HETCOR experiments. For the pyrimidine the signals are easy to assign as a doublet of doublets for H-5’ (*J* = 5.2, 1.3 Hz), H-6’ as a doublet (*J* = 5.2 Hz) and H-2’ (which is the most deshielded hydrogen atom in the pyrimidine moiety) as a doublet with *J* = 1.3 Hz. The most deshielded proton is H-2 in the pyridine moiety at about 10 ppm for both **13a** and **13b**. The multiplicities of the hydrogen atoms in the pyridine moiety are more complex but could be assigned by 2D experiments. Another main feature of the spectra of **13a,b** is the singlet of the methylene protons at ca. 6.70 ppm. The ^13^C NMR spectra present all the expected signals. The C=O is observed at around 190 ppm. The aliphatic carbon atom appears at around 66 ppm.

The new indolizines **14a–f** were obtained in good yields (Table 2) and were characterized through NMR spectroscopy. The ^1^H NMR spectra of the indolizines **14** confirm their structure. The discrimination between the two possible isomers **14** and **14A** was made on the basis of the multiplicity of the proton signals which is unequivocal. The main features are the signals of H-2 which appears as a singlet and H-5, the most deshielded proton appearing as a multiplet in the range of 10.70–10.75 ppm. For the pyrimidine the spectra show three sets of signals for each proton. These signals are easy to assign for H-5’ and H-6’ as a doublet of doublets (*J* = 5.2, 1.4 Hz) and a doublet (*J* = 5.2 Hz) respectively. The hydrogen H-2’ is the most deshielded hydrogen atom in the pyrimidine moiety, as a doublet with *J* = 1.4 Hz. The ^13^C NMR spectra present all the expected signals. The most deshielded carbon atoms are C-2’ and C-5’ at 159 and 157 ppm, respectively for all the series of compounds **14a**–**f**. As in the case of pyrrolo[1,2-*c*]pyrimidines **12**, replacing the acetyl group at C-1 with a carbomethoxy or carboethoxy group induces a shielding effect on the C-1 atom from 115 ppm in the case of **14a**, to 104.8–108.1 ppm for **14b**–**f**.

**Table 2:** The bromide salts **13** and the new 6-pyrimidinylindolizines **14**.

entry	Ar	E	R	yield	mp (°C)

**13a**	C_6_H_5_	—	—	90	214–215
**13b**	4-FC_6_H_4_	—	—	78	218–222
**14a**	4-FC_6_H_4_	COMe	H	64	242–244
**14b**	C_6_H_5_	CO_2_Me	H	68	199–200
**14c**	4-NO_2_C_6_H_4_	CO_2_Et	H	48	247–249
**14d**	4-ClC_6_H_4_	CO_2_Et	H	54	212–214
**14e**	4-FC_6_H_4_	CO_2_Et	H	60	176–178
**14f**	C_6_H_5_	CO_2_Me	CO_2_Me	45	133–136

**Scheme 3:**
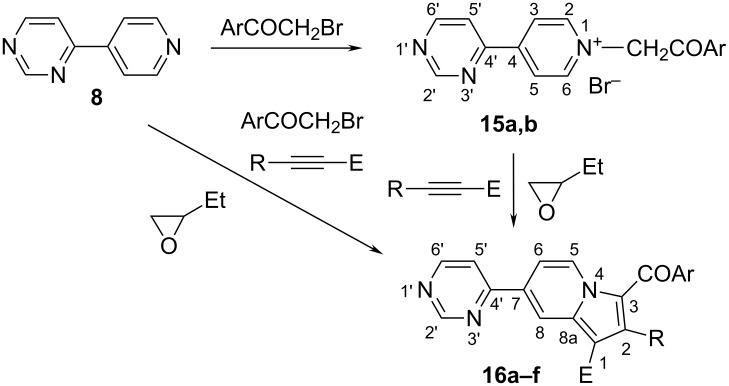
The synthesis of the new pyridinium bromides **15** and 7-pyrimidylindolizines **16a**–**f**.

Similarly, in the case of pyridinium *N*-ylides generated from **8**, the new indolizines **16** were synthesized (Scheme 3) and structurally characterized. Again, in the case of some compounds, the salts were not easily purified and we preferred the multicomponent approach. However, we isolated and characterized the bromides **15a,b** (Table 3). The new 7-(pyrimidyl)indolizines were obtained in moderate to good yields and were characterized by NMR spectroscopy. The ^1^H NMR spectra of the bromides **15** show that the quaternization reaction took place at the pyridine nitrogen atom. The first definitive indications are the signals of the protons in the pyridine moiety, which appear as doublet of doublets either in the starting 4-(4-pyridyl)pyrimidine (**8**) or after quaternization in **15a,b**. The positive charge at the nitrogen induces a deshielding effect for H-2 and H-6 (see numbering in Scheme 3) in **15a,b** compared to **8**. The four protons were assigned as two doublets with *J* = 6.9 Hz. For the pyrimidine, the signals are easily assigned similarly as for the indolizines **14**. Another main feature of the spectra of **15** is the singlet of the methylene protons at 6.6 ppm. The ^13^C NMR spectra present all the expected signals. The C=O is observed at around 190 ppm.

**Table 3:** The bromides **15a,b** and the new 7-pyrimidinylindolizines **16a**–**f**.

entry	Ar	E	R	yield	mp (°C)

**15a**	4-BrC_6_H_4_	—	—	90	264–268
**15b**	4-MeOC_6_H_4_	—	—	88	238–240
**16a**	C_6_H_5_	COMe	H	44	236–238
**16b**	4-ClC_6_H_4_	COMe	H	56	287–289
**16c**	4-FC_6_H_4_	COMe	H	62	240–242
**16d**	4-BrC_6_H_4_	CO_2_Me	H	50	281–282
**16e**	4-MeOC_6_H_4_	CO_2_Me	H	54	249–250
**16f**	3-NO_2_C_6_H_4_	CO_2_Et	H	42	208–210

The new compounds **16** were obtained by a multicomponent approach by mixing all the reagents in 1,2-epoxybutane and heating under reflux for 48 h (Scheme 3). In some cases we carried out the reaction in two steps by isolating the salts **15**, the yields being similar.

The ^1^H NMR spectra of the indolizines **16a**–**f** confirm their structure. All the atoms in the indolizine moiety were assigned by COSY and HETCOR experiments. The main features are the signal for H-2, which appears as a singlet, and those for H-5 which is the most deshielded proton, appearing as a doublet of doublets with *J* = 7.4, 0.8 Hz. The pyrimidine moiety was assigned as previously described for the compounds **15**. The ^13^C NMR spectra present all the expected signals. As in the case of pyrrolo[1,2-*c*]pyrimidines **12** and indolizines **14** replacing the acetyl group at C-1 with a carbomethoxy or carboethoxy group induces a shielding effect on the C-1 atom from 115 ppm for **16a**–**c**, to 108 ppm in the case of **16d**–**f**.

**Scheme 4:**
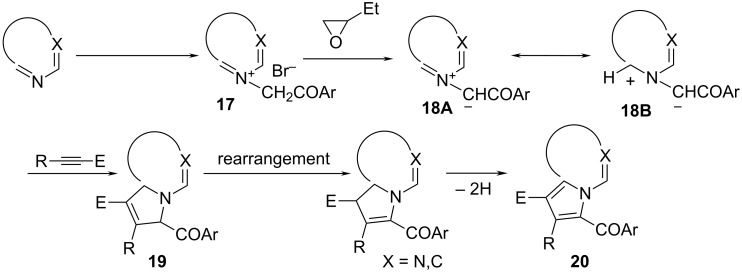
Reaction mechanism.

The reaction mechanism in all cases implies the attack of the bromide salt of type **17** on the oxirane ring in the 1,2-epoxybutane (Scheme 4). The reactive intermediate obtained by the ring opening of the 1,2-epoxybutane abstracts a methylene proton from the salt, generating the corresponding *N*-ylide (**18**) in situ.

The *N*-ylide reacts further with the acetylenic dipolarophile to give a primary cycloadduct **19** which spontaneously rearranges and dehydrogenates under the reaction conditions, to the final aromatic compounds **20**.

### X-ray crystal structures of the starting compounds 4-(2-pyridyl)pyrimidine (**6**) and 4-(4-pyridyl)pyrimidine (**8**)

Full details of the structural solution and refinements are given in Supporting Information File 1. Structural refinements were non-routine owing to subtle disorder occurring in both crystals. Taking into account the values of *Z* (the number of molecules per unit cell) for the crystals of **6** and **8**, their respective space groups, *P*2_1_/*n* and *P*−1, require that each molecule be located on a centre of symmetry and hence be planar. Since neither molecule possesses a centre of symmetry, this implies that they must both be disordered in their respective crystals. Accordingly, centrosymmetric structural models were proposed by considering the possible planar rotamers for both molecules as well as the effect of adding a centre of inversion to each of these. The disordered models assigned were consistent with the observed difference Fourier electron densities, in particular those of the hydrogen atoms, whose relative values indicated that the sites of their parent atoms were either fully occupied by carbon atoms or by nitrogen atoms, or partially occupied by carbon and nitrogen atoms simultaneously.

**Figure 4:**
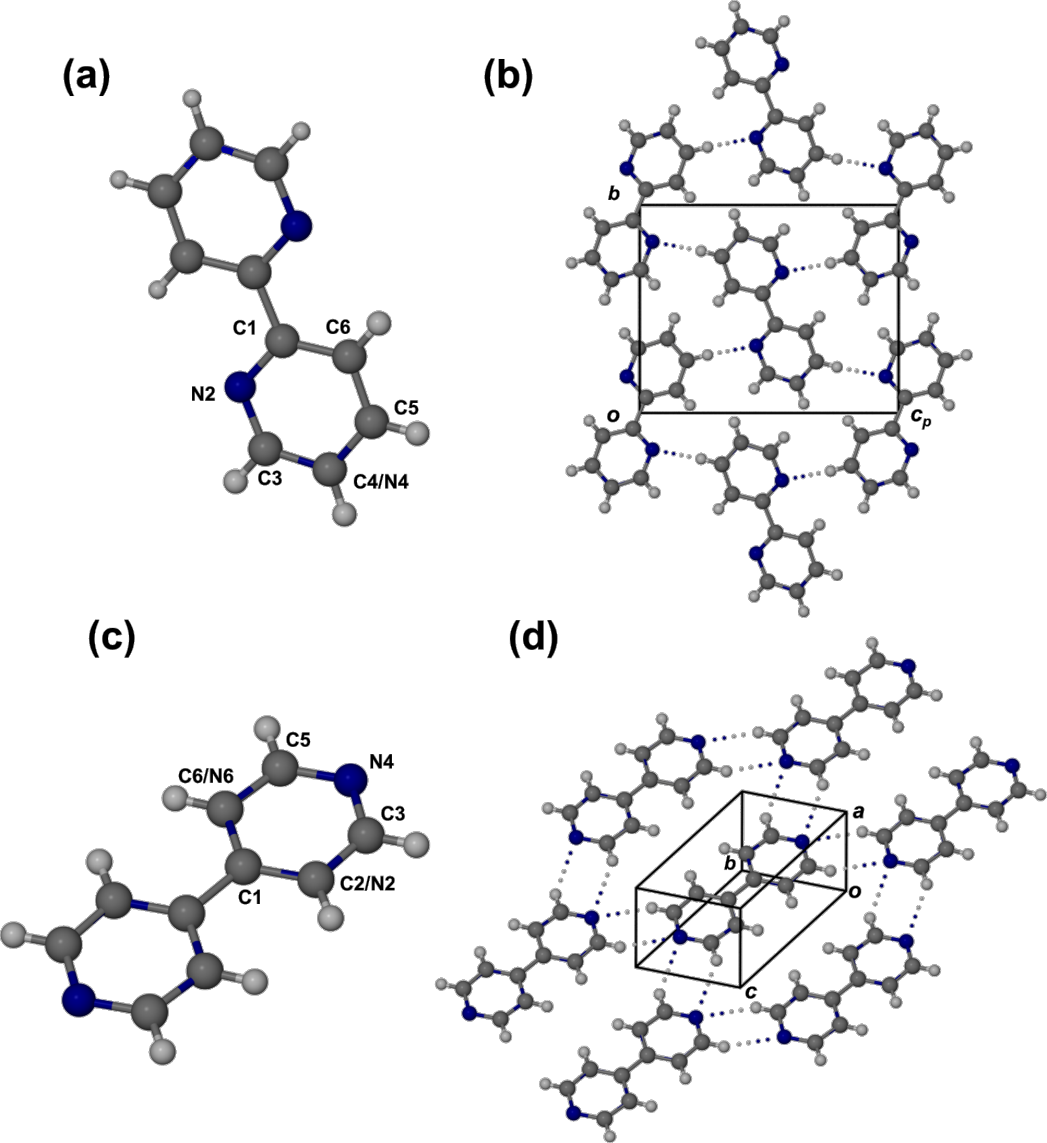
Molecular and crystal structures of **6** (a,b) and **8** (c,d). The molecules are located on centers of inversion and their asymmetric units are labelled, disordered atoms being indicated as C*n*/N*n*.

Figure 4a and 4b show the molecular and crystal structures of 4-(2-pyridyl)pyrimidine (**6**). Here, all atoms in the molecule have a site-occupancy factor (s.o.f.) equal to 1 except the atom labeled ‘C4/N4’, where C and N atoms have equal occupancy (s.o.f. = 0.5 for each and for H-4), which is required to satisfy the condition of centrosymmetry. The molecules are linked into large concatenated rings with graph-set descriptor [33] R_2_^2^(22) by one crystallographically unique, weak C–H^…^N hydrogen bond.

In the centrosymmetric structural model for 4-(4-pyridyl)pyrimidine (**8**) (Figure 4c), atom N4 is necessarily present with s.o.f. = 1, but the evidence from the X-ray analysis indicated the presence of rotamers, with both the 2- and the 6-positions in the two rings occupied by C and N atoms with s.o.f. values of 0.75 and 0.25, respectively, to account for a net total of three carbon atoms and one nitrogen atom. The crystal of **8** is composed of layers, a portion of such a layer being shown in Figure 4d. Two crystallographically distinct cyclic C–H^…^N hydrogen bonded motifs with graph-set descriptor R_2_^2^(6) occur [33] resulting in a more extensive H-bonding in the crystal of **8** than in that of **6**. This is consistent with their significantly different melting points (131–133 °C for **8** and 75–78 °C for **6**). In both crystals, C–H^…^N hydrogen bonding is complemented by offset π-stacking, with shortest ring centroid-to-centroid distances of 3.743 Å in **6** and 3.766 Å in **8**.

It was ascertained from a search of the Cambridge Structural Database [34] (CSD) that the crystal of **6** is isostructural with that of 4,4’-bipyrimidine as the *trans*-rotamer (CSD refcode SACPAN). This is not surprising when one compares this molecule with the centrosymmetric model of Figure 4a. Isostructurality was established from closely matching unit cell parameters and the common space group, and confirmed by the near superposition of their simulated powder X-ray patterns (see Supporting Information File 1). No isostructural analogues for **8** were evident.

## Conclusion

In conclusion, we have obtained 20 new hybrid heteroarenes from the class of indolizines and pyrrolo[1,2-*c*]pyrimidines by a simple multicomponent or two-step approach. All compounds, including the 9 intermediate bromide salts, were structurally characterized by NMR spectroscopy. The biological and fluorescence properties of the new synthesized compounds will be intensely investigated further. Reported methods were employed in obtaining the 4-pyridylpyrimidine isomers used as starting materials. X-ray analyses of the 4-pyridylpyrimidines **6** and **8** were non-trivial, requiring refinement of disordered models due to their location on centers of symmetry.

## Experimental

### General

Melting points were measured using a Boetius hot plate microscope and are uncorrected. ^1^H NMR and ^13^C NMR spectra were recorded on a Varian Gemini 300BB operating at 300 MHz for ^1^H and 75 MHz for ^13^C. The spectra were recorded in CDCl_3_ or DMSO-*d_6_* at 298 K and the chemical shifts are relative to TMS used as the internal standard. The bidimensional correlations spectra (COSY, HETCOR) were performed for complete assignment of chemical shifts. Fourier-transform IR spectra (for representative compounds) were recorded on a Bruker Vertex 70 spectrometer with horizontal device for attenuated reflectance and diamond crystal, in a spectral window ranging from 4000 to 400 cm^−1^ or on a Nicolet Impact 410 Spectrometer in KBr pellets. Elemental analysis was performed on a Perkin Elmer CHNS/O analyzer Series II 2400 apparatus and the results were in agreement with the calculated values. All starting materials and solvents were purchased from common commercial suppliers and were used without purification unless otherwise noted.

### General procedure for obtaining 4-(2-pyridyl)pyrimidinium bromides **11a**–**e**

4-(2-Pyridyl)pyrimidine (10 mmol) and bromoacetophenones (**9**) (10 mmol) were stirred in 50 mL acetone under reflux for 8 h and then were left at room temperature overnight. The precipitated 4-(2-pyridyl)pyrimidinium bromides **11a**–**e** were removed by filtration.

1-[2-Phenyl-2-oxoethyl]-4-(2-pyridyl)pyrimidinium bromide (**11a**). Light brown crystals with mp 227–229 °C. Yield 80%. Anal. calcd for C_17_H_14_BrN_3_O: N, 11.80; found N, 12.13; ^1^H NMR (DMSO-*d*_6_, 300 MHz) δ 6.43 (s, 2H, CH_2_), 7.52–7.68 (m, 2H, H-3”, H-5”), 7.74–7.80 (m, 2H, H-4’, H-4”), 8.06–8.08 (m, 2H, H-2”, H-6”), 8.16 (td, *J* = 7.7, 1.9 Hz, 1H, H-5’), 8.63–8.65 (m, 1H, H-6’), 8.89 (m, 1H, H-3’), 9.04 (dd, *J* = 6.6 Hz, 0.8 Hz, 1H, H-5), 9.32 (dd, *J* = 6.6, 1.7 Hz, 1H, H-6), 9.75 (bs, 1H, H-2); ^13^C NMR (DMSO-*d*_6_, 75 MHz) δ 63.4 (CH_2_), 119.0 (C-5), 125.0 (C-6’), 128.9 (C-2”, C-6”), 129.3 (C-4’), 129.9 (C-3”, C-5”), 133.7, 150.2, 168.5 (C-4, C-1’, C-1”), 135.7 (C-4”), 139.2 (C-5’), 151.5 (C-3’), 154.7 (C-6), 155.3 (C-2), 190.7 (COAr); IR (KBr, cm^−1^): 1696, 1627, 1550, 1450, 1341, 1213.

### General procedure for obtaining pyrrolo[1,2-*c*]pyrimidines **12a**–**h**

4-(2-Pyridyl)pyrimidinium bromide (3 mmol) **11a–e** previously obtained and acetylenic dipolarophiles **10** (3.5 mmol) were stirred under reflux in 20 mL 1,2-epoxybutane for 24 h. The products were precipitated with ethanol and removed by filtration. Further purification was made by crystallization from ethanol or by column chromatography on Al_2_O_3_ using methylene chloride as eluent.

3-(2-Pyridyl)-5-acetyl-7-(4-bromobenzoyl)-pyrrolo[1,2-*c*]pyrimidine (**12a**)*.* Pale yellow powder with mp 263–265 °C. Yield 41%. Anal. calcd for C_21_H_14_BrN_3_O_2_: C, 60.02; H, 3.36; Br, 19.01; N, 10.00; found: C, 60.30; H, 3.65; Br, 19.24; N, 10.22; ^1^H NMR (CDCl_3_, 300 MHz) δ 2.60 (CH_3_), 7.64 (s, 4H, H-2”, H-3”, H-5”, H-6”), 7.80 (s, 1H, H-6), 7.96–8.00 (m, 1H, H-4’), 8.60 (td, *J* = 7.7, 1.9 Hz, 1H, H-5’), 8.70–8.73 (m, 1H, H-6’), 8.86–8.89 (m, 1H, H-3’), 9.30 (d, *J* = 1.6 Hz, 1H, H-4), 10.49 (d, *J* = 1.6 Hz, 1H, H-1); ^13^C NMR (CDCl_3_+TFA, 75 MHz) δ 28.0 (CH_3_), 114.8 (C-4), 117.8 (C-5), 124.6, 128.8, 136.3, 138.5, 142.0, 146.5 (C-3, C-7, C-4a, C-1’, C-1”, C-4”), 124.3 (C-6’), 127.7 (C-4’), 130.3 (C-6), 130.7 (C-2”, C-6”), 132.9 (C-3”, C-5”), 138.6 (C-5’), 142.4 (C-3’), 147.7 (C-1), 185.5 (COAr), 197.5 (CO); IR (ATR, cm^−1^): 3054, 1650, 1627, 1585, 1469, 1328, 1217, 887.

### Procedure for obtaining 4-(3-pyridyl)pyrimidinium bromides **13a**,**b**

4-(3-Pyridyl)pyrimidine (**7**, 10 mmol) and bromoacetophenone (**9**) (10 mmol) were stirred in 50 mL acetone under reflux for 8 h and then were left at room temperature overnight. The precipitated bromides **13a,b** were removed by filtration.

1-[2-Phenyl-2-oxoethyl]-3-(4-pyrimidinyl)pyridinium bromide (**13a**). Red powder with mp 214–215 °C. Yield 90%. Anal. calcd for C_17_H_14_BrN_3_O: N, 11.80; found N, 12.07; ^1^H NMR (DMSO-*d*_6_, 300 MHz) δ 6.71 (s, 2H, CH_2_), 7.65–7.70 (m, 2H, H-3”, H-5”), 7.77–7.83 (m, 1H, H-4”), 8.08–8.12 (m, 2H, H-2”, H-6”), 8.40 (dd, *J* = 5.2, 1.3 Hz, 1H, H-6’), 8.50 (dd, *J* = 8.2, 6.0 Hz, 1H, H-5), 9.14 (d, *J* = 5.2 Hz, 1H, H-5’), 9.24 (dt, *J* = 6.0, 1.4 Hz, 1H, H-6), 9.44 (d, *J* = 1.3 Hz, 1H, H-2’), 9.48 (dt, *J* = 8.2, 1.4 Hz, 1H, H-4), 10.00 (m, 1H, H-2); ^13^C NMR (DMSO-*d*_6_, 75 MHz) δ 66.6 (CH_2_), 118.5 (C-5’), 128.1 (C-5); 128.3 (C-2”, C-6”), 129.1 (C-3”, C-5”), 133.5, 135.5, 156.9 (C-3, C-4’, C-1”), 134.7 (C-4”), 143.9 (C-4), 145.4 (C-2), 147.6 (C-6), 159.0 (C-2’), 159.4 (C-6’), 190.6 (COAr).

### General procedure for obtaining indolizines **14a**–**f**

4-(3-Pyridyl)pyrimidine (**7**, 3 mmol), bromoacetophenones **9** (3 mmol) and different acetylenic dipolarophiles **10** (3.5 mmol) were stirred at reflux in 20 mL 1,2-epoxybutane for 48 h. The products were precipitated with ethanol and removed by filtration. Further purification was made by crystallization from ethanol or column chromatography on Silicagel-60 (Merck, 70–230 mesh) using methylene chloride as eluent.

1-Acetyl-3-(4-fluorobenzoyl)-6-(4-pyrimidinyl)indolizine (**14a**). Yellow crystals with mp 242–244 °C. Yield 64%. Anal. calcd for C_21_H_14_FN_3_O_2_: C, 70.19; H, 3.93; N, 11.69; found C, 70.40; H, 4.21; N, 11.88; ^1^H NMR (CDCl_3_, 300 MHz) δ 2.56 (s, 3H, CH_3_), 7.25 (t, *J* = 8.8 Hz, 1H, H-3”, H-5”), 7.74 (s, 1H, H-2), 7.81 (dd, *J* = 5.2, 1.4 Hz, 1H, H-6’), 7.85–7.93 (m, 2H, H-2”, H-6”), 8.23 (dd, *J* = 9.3, 1.6 Hz, 1H, H-7), 8.74 (dd, *J* = 9.3, 1.1 Hz, 1H, H-8), 8.85 (d, *J* = 5.2, 1H, H-5’), 9.32 (d, *J* = 1.4 Hz, 1H, H-2’), 10.71–10.72 (m, 1H, H-5); ^13^C NMR (CDCl_3_, 75 MHz) δ 28.0 (CH_3_), 115.3 (C-1), 115.8 (*J* = 21.7 Hz, C-3”, C-5”), 116.7 (C-5’), 120.6 (C-8), 126.8 (C-7), 128.8 (C-5), 129.3 (C-2), 131.4 (*J* = 8.9 Hz, C-2”, C-6”), 122.8, 126.1, 135.7, 139.5, 160.9 (C-3, C-8a, C-6, C-4’, C-1”), 157.9 (C-6’), 159.4 (C-2’), 165.1 (*J* = 252.3 Hz, C-4”), 184.3 (COAr), 193.1 (CO); IR (KBr, cm^−1^): 1659, 1630, 1581, 1510, 1485, 1431, 1359, 1213, 1152.

### General procedure for obtaining the 4-(4-pyrimidinyl)pyridinium bromides **15**

4-(4-Pyridyl)pyrimidine (**8**) (10 mmol) and different bromoacetophenones **9** (10 mmol) were stirred in 50 mL acetone under reflux for 8 h and then were left at room temperature overnight. The precipitated bromides **15a,b** were removed by filtration.

1-[2-(4-Bromophenyl)-2-oxoethyl]-4-(4-pyrimidinyl)pyridinium bromide (**15a**). Brown powder with mp 264–268 °C. Yield 90%. Anal. calcd for C_17_H_13_Br_2_N_3_O: N, 9.66; found N, 9.73; ^1^H NMR (DMSO-*d*_6_, 300 MHz) δ 6.61 (s, 2H, CH_2_), 7.90 (d, *J* = 8.7 Hz, 2H, H-3”, H-5”), 8.02 (d, *J* = 8.7 Hz, 2H, H-2”, H-6”), 8.54 (dd, *J* = 5.3, 1.3 Hz, H-5’), 9.01 (d, *J* = 6.9 Hz, 2H, H-3, H-5), 9.21–9.24 (m, 3H, H-2, H-6, H-6’), 9.53 (d, *J* = 1.3 Hz, 1H, H-2’); ^13^C NMR (DMSO-*d*_6_, 75 MHz) δ 66.1 (CH_2_), 119.8 (C-5’), 125.0 (C-3, C-5), 128.9, 132.6, 151.5, 159.3 (C-4, C-1’, C-1”, C-4”), 130.3 (C-2”, C-6”), 132.3 (C-3”, C-5”), 147.2 (C-2, C-6), 159.6 (C-6’), 159.9 (C-2’), 190.0 (COAr).

### General procedure for obtaining the indolizines **16a**–**f**

4-(4-Pyridyl)pyrimidine (**8**) (3 mmol), bromoacetophenones **9** (3 mmol) and acetylenic dipolarophile **10** (3.5 mmol) in 20 mL 1,2-epoxybutane were stirred under reflux for 48 h. The products were precipitated with ethanol and removed by filtration. Further purification was performed by crystallization from ethanol or column chromatography on Silicagel-60 (Merck) or neutral Al_2_O_3_ by using methylene chloride as an eluent.

1-Acetyl-3-benzoyl-7-(4-pyrimidinyl)indolizine (**16a**). Yellow crystals with mp 236–238 °C. Yield 44%. Anal. calcd for C_21_H_15_N_3_O_2_: C, 73.89; H, 4.43; N, 12.31; found C, 74.11; H, 4.69; N, 12.59; ^1^H NMR (CDCl_3_, 300 MHz) δ 2.51 (s, 3H, Me), 7.48–7.58 (m, 3H, H-3”, H-4”, H-5”), 7.69 (s, 1H, H-2), 7.78–7.81 (m, 2H, H-2”, H-6”), 7.91 (dd, *J* = 5.3, 1.4 Hz, 1H, H-5’), 7.95 (dd, *J* = 7.4, 1.9 Hz, 1H, H-6), 8.83 (d, *J* = 5.4 Hz, 1H, H-6’), 9.28 (dd, *J* = 1.9, 0.8 Hz, 1H, H-8), 9.30 (d, *J* = 1.4 Hz, 1H, H-2’), 9.97 (dd, *J* = 7.4, 0.8 Hz, 1H, H-5); ^13^C NMR (CDCl_3_, 75 MHz) δ 28.0 (Me), 114.0 (C-6), 116.5 (C-1), 117.4 (C-5’), 119.0 (C-8), 123.2, 136.5, 138.9, 139.7, 161.0 (C-3, C-8a, C-7, C-1’, C-1”), 129.1 (C-5), 129.3 (C-2), 128.7, 129.1, 132.0 (C-2”, C-6”, C-3”, C-4”, C-5”), 158.1 (C-6’), 159.4 (C-2’), 185.9 (COAr), 193.4 (COMe); IR (KBr, cm^−1^): 1655, 1616, 1575, 1507, 1394, 1348, 1229, 1193.

## Supporting Information

Experimental procedures and structural characterization (^1^H, ^13^C NMR data) for all newly synthesized compounds.

File 1 Additional experimental data.
