# Supplementary material for: Indolizines and pyrrolo[1,2-c]pyrimidines decorated with a pyrimidine and a pyridine unit respectively
Source: Beilstein J Org Chem. 2015 Jun 26;11:1079–88. doi: 10.3762/bjoc.11.121 (PMC4505085; doi:10.3762/bjoc.11.121)
Supplement: File 1 — Additional experimental data. [file Beilstein_J_Org_Chem-11-1079-s001.pdf]

Supporting Information

for

Indolizines and pyrrolo[1,2-*c*]pyrimidines decorated with a pyrimidine and a pyridine unit respectively

Marcel Mirel Popa*[1,2], Emilian Georgescu[3], Mino R. Caira*[4], Florentina Georgescu[5], Constantin Draghici[1], Raluca Stan[2], Calin Deleanu[1] and Florea Dumitrascu[1]

Address: [1]Center for Organic Chemistry C.D. Nenitzescu, Romanian Academy, Spl. Independentei 202B, Bucharest 060023, Romania; [2]Faculty of Applied Chemistry and Materials Science, 'Politehnica' University of Bucharest, Polizu Street 1-7, 011061 Bucharest, Romania; [3]Research Center Oltchim, Str. Uzinei 1, RO 240050, Ramnicu Valcea, Romania. [4]Department of Chemistry, University of Cape Town, Rondebosch 7701, South Africa and [5]Research Dept., Teso Spec SRL, Str. Muncii 53, RO-915200 Fundulea, Calarasi, Romania

Email: Marcel Mirel Popa* - mirelupb@gmail.com;

Mino R. Caira* - mino.caira@uct.ac.za

* Corresponding author

Additional experimental data

Contents:

1. General……………………………………………………………………………………….….…S2
2. Structural elucidation of compounds 6 and 8 by single crystal X-ray analysis ..……....S2
3. 4-(2-Pyridyl)pyrimidinium bromides 11a-e……………..………………………………….……..….S7
4. Pyrrolo[1,2-*c*]pyrimidines 12a-h……………………………………………..………………….…S10
5. 4-(3-Pyridyl)pyrimidinium bromides 13a,b...S14
6. 6-(Pyrimidinyl)indolizines 14a-e ……………………..…………………….…..……..........…S15
7. 4-(4-Pyrimidinyl)pyridinium bromides 15a,b………………………...............................S18
8. 7-(4-Pyrimidinyl)indolizines 16a-f………………...S18
9. Annexes (Spectral Data)……………………………………………………….……..…….S21

1. General

Melting points were measured using a Bőetius hot plate microscope and are uncorrected. ^{1}H NMR and ^{13}C NMR spectra were recorded on a Varian Gemini 300BB operating at 300 MHz for ^{1}H and 75 MHz for ^{13}C. The spectra were recorded in $CDCl_3$ and DMSO at 298K and the chemical shifts are relative to TMS used as the internal standard. The bidimensional correlation spectra (COSY, HETCOR) were performed for complete assignment of chemical shifts. Fourier-transform IR spectra were recorded on a Bruker Vertex 70 spectrometer with horizontal device for attenuated reflectance and diamond crystal, on a spectral window ranging from 4000 to 400 cm^{-1} or on a Nicolet Impact Spectrometer 410 in KBr pellets. Elemental analysis was performed on a Perkin Elmer CHNS/O Analyser Series II 2400 apparatus and the results were in agreement with the calculated values. All starting materials and solvents were purchased from common commercial suppliers and were used without purification unless otherwise noted.

2. Structural elucidation of compounds 6 and 8 by single crystal X-ray analysis

Experimental:

All experimental conditions and diffractometer details are listed in the CIF files that accompany the submission. These files also list all relevant crystallographic data

(atomic co-ordinates, thermal displacement parameters, bond lengths and angles, and torsion angles) as well as software employed in data-collection, data-processing, structural solutions and refinements. The strategy for refining the disordered structures is described below.

Crystal data for 6: $C_9H_7N_3$, M = 157.18, light brown block, 0.20 × 0.16 × 0.14 mm^3, monoclinic, space group $P2_1/n$ (No. 14), a = 3.7427(2), b = 8.9922(6), c = 11.2378(6) Å, β = 95.192(3)°, V = 376.66(4) $Å^3$, Z = 2, D_c = 1.386 g/cm^3, F_{000} = 164, Nonius Kappa CCD diffractometer, MoKα radiation, λ = 0.71073 Å, T = 173(2)K, $2\theta_{max}$ = 56.6°, 1769 reflections collected, 913 unique (R_{int} = 0.0176). Final $GooF$ = 1.073, R_1 = 0.0380, wR_2 = 0.0985, R indices based on 748 reflections with I > 2σ(I) (refinement on F^2), 55 parameters, 0 restraints. Lorentz-polarization and absorption corrections applied, μ = 0.088 mm^{-1}, **CCDC number 1050641**.

Crystal data for 8: $C_9H_7N_3$, M = 157.18, light brown block, 0.20 × 0.17 × 0.16 mm^3, triclinic, space group P-1 (No. 2), a = 3.7657(5), b = 5.7899(7), c = 8.8023(11) Å, α = 99.719(9), β = 92.247(7), γ = 100.966(7)°, V = 185.20(4) $Å^3$, Z = 1, D_c = 1.409 g/cm^3, F_{000} = 82, Nonius Kappa CCD diffractometer, MoKα radiation, λ = 0.71073 Å, T = 173(2)K, $2\theta_{max}$ = 52.8°, 1252 reflections collected, 744 unique (R_{int} = 0.0156). Final $GooF$ = 1.111, R_1 = 0.0673, wR_2 = 0.1587, R indices based on 682 reflections with I > 2σ(I) (refinement on F^2), 55 parameters, 0 restraints. Lorentz-polarization and absorption corrections applied, μ = 0.090 mm^{-1}, **CCDC number 1050642**.

Details of structural modelling

As explained in the manuscript, since the molecules of compound **6** (Z = 2 molecules per unit cell) and compound **8** (Z = 1) are not centrosymmetric, but are required to be located on centres of inversion in their respective space groups (monoclinic, $P2_1$/n and triclinic, P(-1)), it was necessary to postulate (planar) centrosymmetric molecular models.

The initial structure solutions by direct methods yielded the expected hexagonal asymmetric unit (symbolic co-ordinates x, y, z) in each case, to which the second

hexagon (at -x, -y, -z) of each molecule could be added by applying the centre of inversion.

Compound **6**: The two planar rotamers (**6a, 6b**) are shown in Figure S1, together with the results of adding a centre of inversion '(-1)' to each. Thus, if the rotamer 6a were to crystallize as such, the requirement of centrosymmetry would result in model A, with the N atoms at positions 2, 4 and 6, each having a site-occupancy factor (s.o.f.) of 0.5 (and therefore each being coincident with carbon atoms, also having a s.o.f. of 0.5 each).

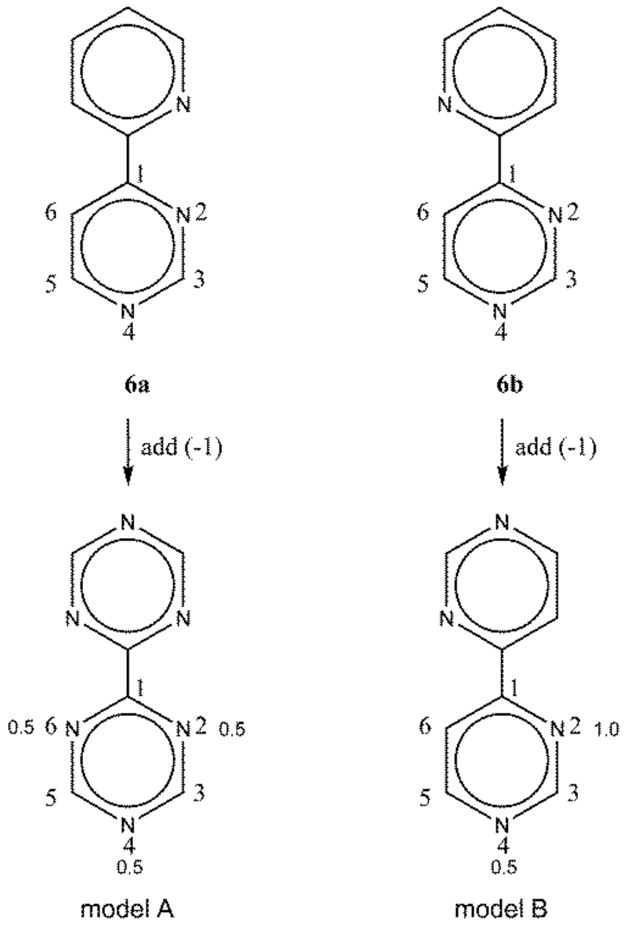

Figure S1: Centrosymmetric models of **6** arising from its planar rotamers with key s.o.f.s included.

If, instead, the rotamer **6b** were to crystallize, model B would result in the crystal. These models should be distinguishable most obviously from the pattern of H atoms appearing in difference Fourier syntheses. Specifically, for model A, atoms 2-6 should all

have attached H atoms, those at positions associated with atoms 3 and 5 having full occupancy and those at positions associated with atoms 2, 4 and 6 only half occupancy. Instead, for model B the ring should contain no H atom attached to N at position 2, full H atoms associated with positions 3, 5 and 6, and a H atom with half occupancy associated with position 4.

During the refinement of the structure of 6 with isotropic thermal parameters, it was quite clear that the H atoms appeared at the expected positions corresponding to model B, at ~ 1Å away from the parent atoms with difference electron densities at the positions indicated having the values shown in parentheses: 2 (0.0 eÅ^{-3}), 3 (0.60 eÅ^{-3}), 4 (0.37 eÅ^{-3}), 5 (0.72 eÅ^{-3}), 6 (0.70 eÅ^{-3}). This enabled unequivocal assignment of atom 2 as a nitrogen with s.o.f. =1.0. During the refinement, the s.o.f.s of the atoms thus corresponded to those of model B, with H atoms assigned the corresponding s.o.f.s. (in particular with H4 at half-occupancy). The SHELX commands EXYZ and EADP together with appropriate PART instructions ensured that position 4 was modelled with the co-ordinates of C4 and N4 coinciding during and after refinement, each with s.o.f.s equal to 0.5.

Similar considerations were given to obtaining an appropriate centrosymmetric model for the molecule of **8**. Figure S2 illustrates the model obtained by the same reasoning as before, taking into account rotameric possibilities and the operation of addition of a centre of inversion to the molecule. In this case the N atom at position 2 has equal statistical probability of occurring at the four equivalent positions shown in the model.

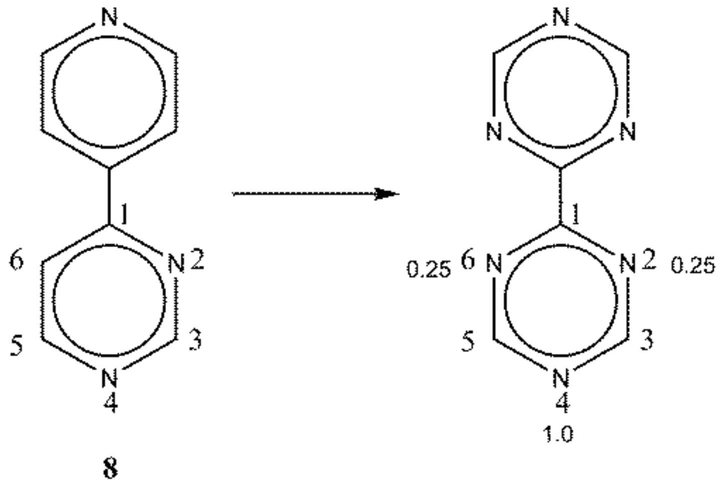

Figure S2: Centrosymmetric model of **8** arising from its planar rotamers with key s.o.f.s included.

Since carbon atoms dominate (s.o.f. = 0.75) at the four locations equivalent to N2, and full carbon atoms occur at positions 3 and 5, it follows that almost equal electron densities for H atoms associated with positions 2, 3, 5 and 6 should be observed. In fact, the observed difference electron density values at these four positions were in the range 0.52-0.58 e$Å^{-3}$, confirming the validity of the model.

Following isotropic refinement, anisotropic thermal displacement parameters were introduced (with EADP maintained). All H atoms were placed in a riding model with U_{iso} = 1.2U_{eq}(parent atom).

<u>*Hydrogen bond data*</u>

D-H···A	D-H (Å)	H···A (Å)	D···A (Å)	D-H···A°
Compound 6				
C5-H5···N2[a]	0.95	2.66	3.552	156
Compound 8				
C3-H3···N4[b]	0.95	2.67	3.484	144
C5-H5···N4[c]	0.95	2.70	3.514	144

Symmetry code: [a] *-½+x, ½-y, ½+z;* [b] *1-x, -y, -z;* [c] *2-x, 1-y, -z*

Isostructurality of 6 and 4,4'-bipyrimidine

Reference is made to this instance of crystal isostructurality in the manuscript. The crystallographic data for compound **6** and those for 4,4'-bipyrimidine ([1], refcode SACPAN in the Cambridge Structural Database) were used as input to the program *Lazy Pulverix* [2] to compute their powder X-ray diffraction patterns. These are shown as Figure S3 below, from which it is clear that the *two phases are isostructural.*

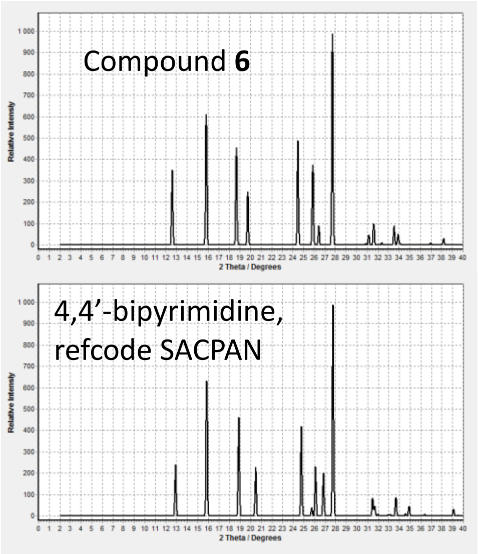

Figure S3: Computed PXRD patterns (CuKα-radiation, λ = 1.5418 Å) for 6 and SACPAN.

References:

[1] Tapolsky, G.; Robert, F.; Launay, J.P. *New J.Chem.* **1988,** *12*, 761.

[2] Yvon, K.; Jeitschko, W.; Parthé, E. *J. Appl. Crystallogr*. **1977,** *10*, 73–74.

3. General procedure for obtaining 4-(2-pyridyl)pyrimidinium bromides 11a-e.

4-(2-Pyridyl)pyrimidine (10 mmol) and bromoacetophenone **9** (10 mmol) were stirred in 50 mL acetone under reflux for 8 h and then were left at room temperature overnight. The precipitated 4-(2-pyridyl)pyrimidinium bromides **11a-e** were removed by filtration.

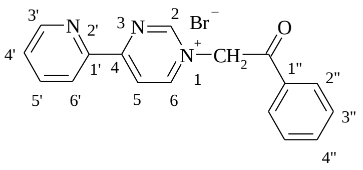

1-[2-Phenyl-2-oxoethyl]-4-(2-pyridyl)pyrimidinium bromide (11a).

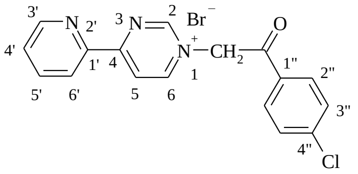

1-[2-(4-Chlorophenyl)-2-oxoethyl]-4-(2-pyridyl)pyrimidinium bromide (11b). Brown crystals with mp 222-223 °C. Yield 87%. Calcd. $C_{17}H_{13}BrClN_3O$: N 10.76. Found N 10.98; ^{1}H-NMR (DMSO, 300 MHz, δ): 6.54 (s, 2H, CH_2); 7.78 (d, *J* = 8.5 Hz, 2H, H-3”, H-5”); 7.77-7.84 (m, 1H, H-4’); 8.13 (d, *J* = 8.5 Hz, 2H, H-2”, H-6”); 8.21 (td, *J* = 7.7, 1.9 Hz, 1H, H-5’); 8.66-8.69 (m, 1H, H-6’); 8.94-8.96 (m, 1H, H-3’); 9.13 (dd, *J* = 6.6 Hz, 0.8 Hz, 1H, H-5); 9.44 (dd, *J* = 6.6, 1.7 Hz, 1H, H-6); 9.87 (bs, 1H, H-2). ^{13}C-NMR (DMSO, 75 MHz, δ): 62.8 (CH_2); 118.5 (C-5), 124.3 (C-6’); 128.6 (C-4’); 129.4 (C-2”, C-6”): 130.3 (C-3”, C-5”); 132.1, 139.7, 149.8, 167.3 (C-4, C-1’, C-1”, C-4”); 138.6 (C-5’); 151.0 (C-3’); 154.4 (C-6); 154.8 (C-2); 189.4 (COAr); IR (KBr, cm^{-1}): 1694, 1626, 1547, 1450, 1336, 1237, 1210, 1091.

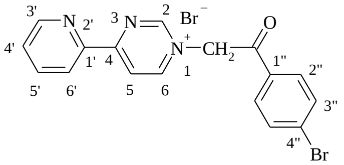

1-[2-(4-Bromophenyl)-2-oxoethyl]-4-(2-pyridyl)pyrimidinium bromide (11c). Brown crystals with mp 242-243 °C. Yield 90%. Calcd. $C_{17}H_{13}Br_2N_3O$: N 9.66. Found N 9.93; ^{1}H-NMR (DMSO, 300 MHz, δ): 6.56 (s, 2H, CH_2); 7.79-7.83 (m, 1H, H-4’);7.91(d, *J* = 8.8 Hz, 2H, H-3”, H-5”); 8.05 (d, *J* = 8.8 Hz, 2H, H-2”, H-6”); 8.20 (td, *J* = 7.7, 1.9 Hz, 1H, H-5’); 8.65-8.68 (m, 1H, H-6’); 8.93-8.95 (m, 1H, H-3’); 9.13 (dd, *J* = 6.6 Hz, 0.8 Hz, 1H, H-5); 9.46 (dd, *J* = 6.6, 1.7 Hz, 1H, H-6); 9.91 (bs, 1H, H-2); ^{13}C-NMR (DMSO, 75 MHz, δ): 62.8 (CH_2); 118.5 (C-5), 124.3 (C-6’); 128.7 (C-4’); 130.4 (C-2”, C-6”): 132.4 (C-3”, C-5”); 129.0, 132.5, 149.8, 167.7 (C-4, C-1’, C-1”, C-4”); 138.6 (C-5’); 150.9 (C-3’); 154.4 (C-6); 154.8 (C-2); 189.6 (COAr); IR (KBr, cm^{-1}): 1694, 1626, 1585, 1547, 1450, 1337, 1235, 1209, 1181.

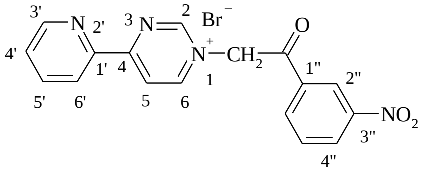

1-[2-(3-Nitrophenyl)-2-oxoethyl]-4-(2-pyridyl)pyrimidinium bromide (11d). Brown crystals with mp 219-221 ^{o}C. Yield 80%. Calcd. $C_{17}H_{13}BrN_4O_3$: N 13.96. Found N 14.15; ^{1}H-NMR (DMSO, 300 MHz, δ): 6.67 (s, 2H, CH_2); 7.80-7.84 (m, 1H, H-4'); 8.00 (t, *J* = 8.0, 1H, H-5"); 8.21 (td, *J* = 7.7, 1.9 Hz, 1H, H-5'); 8.53-8.56 (m, 1H, H-4"); 8.61-8.65 (m, 1H, H-6"); 8.67-8.69 (m, 1H, H-6'); 8.79 (t, *J* = 1.9 Hz, 1H, H-2"); 8.94-8.96 (m, 1H, H-3'); 9.16 (dd, *J* = 6.6 Hz, 0.8 Hz, 1H, H-5); 9.47 (dd, *J* = 6.6, 1.7 Hz, 1H, H-6); 9.92 (bs, 1H, H-2); ^{13}C-NMR (DMSO, 75 MHz, δ): 63.0 (CH_2); 118.6 (C-5), 122.7, 128.8, 131.2, 134,6 (C-2", C-4", C-5", C-6"); 124.3 (C-6'); 128.7 (C-4'); 134.8, 148.1, 149.8, 167.9 (C-4, C-1', C-1", C-3"); 138.6 (C-5'); 150.9 (C-3'); 154.4 (C-6); 154.8 (C-2); 189.7 (COAr); IR (KBr, cm^{-1}): 1708, 1631, 1528, 1457, 1353, 1214.

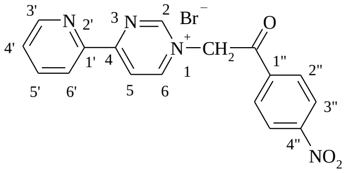

1-[2-(4-Nitrophenyl)-2-oxoethyl]-4-(2-pyridyl)pyrimidinium bromide (11e). Brown crystals with mp 226-228 ^{o}C. Yield 78%. Calcd. $C_{17}H_{13}BrN_4O_3$: N 13.96. Found N 14.28; ^{1}H-NMR (DMSO, 300 MHz, δ): 6.57 (s, 2H, CH_2); 7.80-7.85 (m, 1H, H-4'); 8.21 (td, *J* = 7.7, 1.9 Hz, 1H, H-5'); 8.34(d, *J* = 9.0 Hz, 1H, H-3", H-5"); 8.48 (d, *J* = 9.0 Hz, 1H, H-2", H-6"); 8.68-8.70 (m, 1H, H-6'); 8.95-8.97 (m, 1H, H-3'); 9.16 (dd, *J* = 6.6 Hz, 0.8 Hz, 1H, H-5); 9.42 (dd, *J* = 6.6, 1.7 Hz, 1H, H-6); 9.86 (bs, 1H, H-2); ^{13}C-NMR (DMSO, 75 MHz, δ): 63.2 (CH_2); 118.6 (C-5), 124.3 (C-2", C-6"): 124.3 (C-6'); 128.7 (C-4'); 129.9 (C-3", C-5"); 138.1, 149.8, 150.7, 167.9 (C-4, C-1', C-1", C-4"); 138.6 (C-5'); 150.9 (C-3'); 154.4 (C-6); 154.8 (C-2); 189.7 (COAr); IR (KBr, cm^{-1}): 1705, 1627, 1549, 1520, 1453, 1337, 1211.

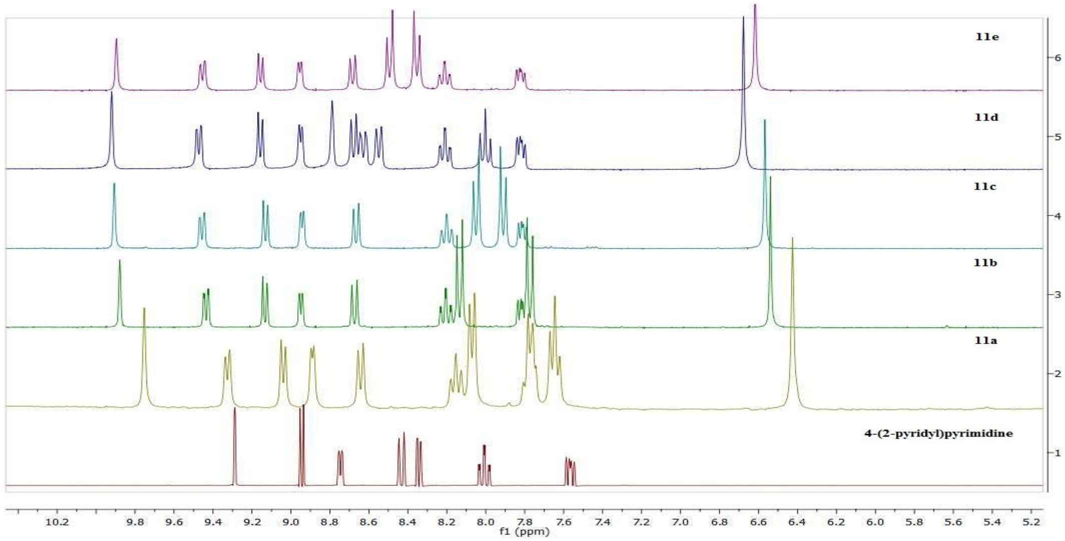

4. General procedure for obtaining pyrrolo[1,2-*c*]pyrimidines 12a-h.

4-(2-Pyridyl)pyrimidinium bromide (3 mmol) **11a-e** previously obtained and acetylenic dipolarophiles **10** (3.5 mmol) were stirred under reflux in 20 mL 1,2-epoxybutane for 24 h. The products were precipitated with ethanol and removed by filtration. Further purification was made by crystallization from ethanol or by column chromatography on Al_2O_3 using methylene chloride as eluent.

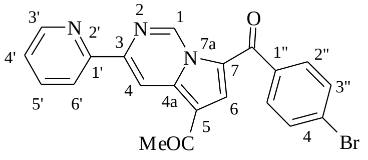

3-(2-Pyridyl)-5-acetyl-7-(4-bromobenzoyl)-pyrrolo[1,2-*c*]pyrimidine (12a).

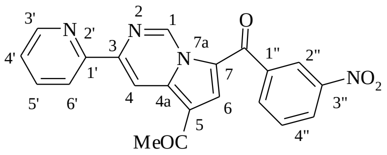

3-(2-Pyridyl)-5-acetyl-7-(3-nitrobenzoyl)-pyrrolo[1,2-*c*]pyrimidine (12b). Pale yellow powder with mp 255-257 ^oC. Yield 46%. Calcd. $C_{21}H_{14}N_4O_4$: C 65.28, H 3.65, N 14.50. Found C 65.52, H 3.47, N 14.61; ^1H-NMR ($CDCl_3$+TFA, 300 MHz, δ): 2.75 (CH3); 7.84 (t, *J* = 8.0, 1H, H-5”); 7.91 (s, 1H, H-6); 8.12-8.18 (m, 1H, H-4’); 8.25-8.29 (m, 1H, H-4”); 8.57-8.60 (m, 1H, H-6”); 8.70 (t, *J* = 1.9 Hz, 1H, H-2”); 8.73-8.79 (m, 1H, H-5’); 8.88-8.90 (m, 1H, H-6’); 9.03-9.05 (m, 1H, H-3’); 9.47 (d, *J* = 1.6 Hz, 1H, H-4); 10.67 (d, *J* = 1.6 Hz,

1H, H-1). ^{13}C-NMR ($CDCl_3$+TFA, 75 MHz, δ): 28.0 (CH3); 114.8 (C-4); 117.9 (C-5), 123.9, 127.7, 130.7, 134.8 (C-2", C-4", C-5", C-6"); 124.2, 138.9, 142.0, 146.5, 148.4 (C-3, C-7, C-4a, C-1', C-1"); 124.4 (C-6'); 127.8 (C-4'); 130.2 (C-6); 139.0 (C-5'); 142.5 (C-3'); 147.7 (C-1); 148.4 (C-3"); 183.7 (COAr); 197.7 (CO); IR (ATR, cm^{-1}): 1661, 1612, 1516, 1470, 1417, 1347, 1216.

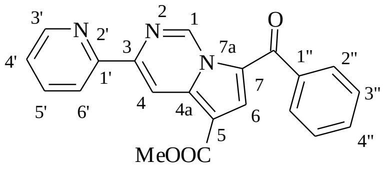

Methyl 3-(2-pyridyl)-7-benzoylpyrrolo[1,2-*c*]pyrimidine-5-carboxylate (12c). Pale yellow powder with mp 218-219 ^{o}C. Yield 52%. Calcd. $C_{21}H_{15}N_3O_3$: C 70.58, H 4.23, N 11.76. Found C 70.42, H 4.51, N 11.98; ^{1}H-NMR ($CDCl_3$, 300 MHz, δ): 3.97 (s, 2H, Me); 7.32-7.37 (m, 1H, H-4'); 7.51-7.57 (m, 2H, H-3", H-5"); 7.60-7.65 (m, 1H, H-4"); 7.82-7.89 (m, 3H, H-5', H-2", H-6"); 7.86 (s, 1H, H-6); 8.46 (dt, *J* = 7.7, 1.1 Hz, 1H, H-6'); 8.72-8.74 (m, 1H, H-3'); 9.18 (d, *J* = 1.4 Hz, 1H, H-4); 10.60 (d, *J* = 1.4 Hz, 1H, H-1). ^{13}C-NMR ($CDCl_3$, 75 MHz, δ): 51.8 (CH_3); 108.1(C-5), 110.3 (C-4); 121.7 (C-6'); 122.8, 138.9, 140.4, 148.6 153.8 (C-3, C-7, C-4a, C-1', C-1"); 124.4 (C-4'); 128.6 (C-2", C-6'); 129.1 (C-3", C-5"); 130.4 (C-2'); 132.3 (C-4"); 137.1 (C-5'); 140.7 (C-3'); 149.8 (C-1); 163.9 (CO); 185.5 (COAr); IR (ATR, cm^{-1}): 1717, 1620, 1523, 1467, 1337, 1216.

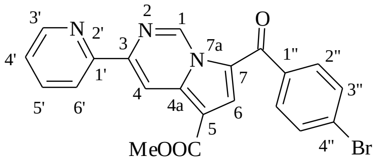

Methyl 3-(2-pyridyl)-7-(4-bromobenzoyl)pyrrolo[1,2-*c*]pyrimidine-5-carboxylate (12d). Pale yellow powder with mp 234-236 ^{o}C. Yield 62%. Calcd. $C_{21}H_{14}BrN_3O_3$: C 57.82, H 3.23, Br 18.32, N 9.63. Found C 58.07, H 3.44, Br 18.60, N 9.91; ^{1}H-NMR ($CDCl_3$, 300 MHz, δ): 3.98 (s, 2H, Me); 7.34-7.39 (m, 1H, H-4'); 7.69(d, *J* = 8.8 Hz, 2H, H-3", H-5"); 7.74 (d, *J* = 8.8 Hz, 2H, H-2", H-6"); 7.86 (s, 1H, H-6); 7.87 (td, *J* = 7.7, 1.9 Hz, 1H, H-5'); 8.46 (dt, *J* = 7.7, 1.1 Hz, 1H, H-6'); 8.93-8.95 (m, 1H, H-3'); 9.19 (d, *J* = 1.4 Hz, 1H, H-4); 10.57 (d, *J* = 1.4 Hz, 1H, H-1); ^{13}C-NMR ($CDCl_3$, 75 MHz, δ): 51.9

(CH_3); 108.3 (C-5), 110.4 (C-4); 121.9 (C-6'); 122.4, 127.2, 137.7, 148.9, 153.7 (C-3, C-7, C-4a, C-1', C-1", C-4"); 124.6 (C-4'); 130.3 (C-6); 130.6 (C-2", C-6'); 132.0 (C-3", C-5"); 137.2 (C-5'); 140.7 (C-3'); 149.9 (C-1); 163.8 (CO); 184.3 (COAr); IR (ATR, cm^{-1}): 1702, 1615, 1467, 1349, 1241, 1212.

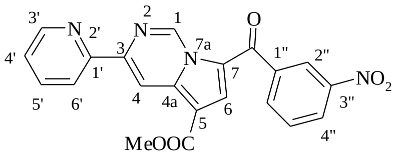

Methyl 3-(2-pyridyl)-7-(3-nitrobenzoyl)pyrrolo[1,2-*c*]pyrimidine-5-carboxylate (12e). Pale yellow powder with mp 233-235 ^oC. Yield 54%. Calcd. $C_{21}H_{14}N_4O_5$: C 62.69, H 3.51, N 13.92. Found C 62.86, H 3.84, N 14.21. ATR-IR: 1203, 1345, 1525, 1623, 1709, 2957, 3060 cm^{-1}; ^1H-NMR ($CDCl_3$, 300 MHz, δ): 3.98 (s, 2H, Me); 7.36-7.41 (m, 1H, H-4'); 7.77 (t, *J* = 8.0, 1H, H-5"); 7.86 (s, 1H, H-6); 7.88 (td, *J* = 7.7, 1.9 Hz, 1H, H-5'); 8.17-8.20 (m, 1H, H-4"); 8.40-8.46 (m, 2H, H-6', H-6"); 8.70 (t, *J* = 1.9 Hz, 1H, H-2"); 8.75-8.76 (m, 1H, H-3'); 9.22 (d, *J* = 1.4 Hz, 1H, H-4); 10.60 (d, *J* = 1.4 Hz, 1H, H-1); ^{13}C-NMR ($CDCl_3$, 75 MHz, δ): 52.0 (CH_3); 108.8 (C-5), 110.4 (C-4); 121.9 (C-6'); 123.9, 126.5, 130.0, 134.6 (C-2", C-4", C-5", C-6"); 140.4, 141.1, 148.9, 149.4, 153.6, (C-3, C-7, C-4a, C-1', C-1", C-3"); 124.7 (C-4'); 130.3 (C-6); 137.3 (C-5'); 140.6 (C-3'); 149.9 (C-1); 163.6 (CO); 182.6 (COAr); IR (ATR, cm^{-1}); 1709, 1623, 1581, 1525, 1467, 1345, 1203.

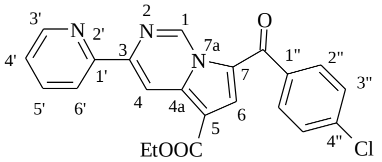

Ethyl 3-(2-pyridyl)-7-(4-chlorobenzoyl)pyrrolo[1,2-*c*]pyrimidine-5-carboxylate (12f). Pale yellow powder with mp 204-205 ^oC. Yield 40%. Calcd. $C_{22}H_{16}ClN_3O_3$: C 65.11, H 3.97, Cl 8.74, N 10.35. Found C 65.37, H 4.14, Cl 8.96, N 10.71; ^1H-NMR ($CDCl_3$, 300 MHz, δ): 1.45 (t, *J* = 7.1 Hz, 3H, CH_3); 4.44 (q, *J* = 7.1 Hz, 2H, CH_2); 7.32-7.37 (m, 1H, H-4'); 7.52 (d, *J* = 8.5 Hz, 2H, H-3", H-5"); 7.81 (d, *J* = 8.5 Hz, 2H, H-2", H-6"); 7.83 (s, 1H, H-6); 7.85 (td, *J* = 7.7, 1.9 Hz, 1H, H-5'); 8.45 (dt, *J* = 7.7, 1.1 Hz, 1H, H-6'); 8.72-

8.75 (m, 1H, H-3’); 9.20 (d, *J* = 1.4 Hz, 1H, H-4); 10.54 (d, *J* = 1.4 Hz, 1H, H-1); ^{13}C-NMR ($CDCl_3$, 75 MHz, δ): 14.6 (CH_3); 60.7 (CH_2); 108.5 (C-5), 110.3 (C-4); 121.7 (C-6’); 122.3, 137.3, 138.6, 140.6, 148.7, 153.7 (C-3, C-7, C-4a, C-1’, C-1”, C-4”); 124.4 (C-4’); 129.0 (C-2”, C-6’); 130.5 (C-3”, C-5”);130.0 (C-6); 137.0 (C-5’); 140.5 (C-3’); 149.8 (C-1); 163.3 (CO); 184.0 (COAr); IR (ATR, cm^{-1}); 1703, 1631, 1585, 1467, 1331, 1200.

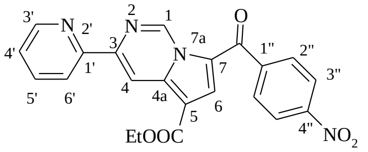

Ethyl 3-(2-pyridyl)-7-(4-nitrobenzoyl)pyrrolo[1,2-*c*]pyrimidine-5-carboxylate (12g). Pale yellow powder with mp 218-220 ^oC. Yield 48%. Calcd. $C_{22}H_{16}N_4O_5$: C 63.46, H 3.87, N 13.46. Found C 63.74, H 4.14, N 13.76; ^{1}H-NMR ($CDCl_3$, 300 MHz, δ): 1.45 (t, *J* = 7.1 Hz, 3H, CH_3); 4.46 (q, *J* = 7.1 Hz, 2H, CH_2); 7.37-7.41 (m, 1H, H-4’); 7.83 (s, 1H, H-6); 7.89 (td, *J* = 7.7, 1.9 Hz, 1H, H-5’); 8.02 (d, *J* = 8.8 Hz, 2H, H-3”, H-5”); 8.42 (d, *J* = 8.8 Hz, 2H, H-2”, H-6”); 8.48 (dt, *J* = 7.7, 1.1 Hz, 1H, H-6’); 8.75-8.77 (m, 1H, H-3’); 9.30 (d, *J* = 1.4 Hz, 1H, H-4); 10.64 (d, *J* = 1.4 Hz, 1H, H-1). ^{13}C-NMR ($CDCl_3$, 75 MHz, δ): 14.7 (CH_3); 61.0 (CH_2); 109.2 (C-5), 110.6 (C-4); 122.0 (C-6’); 122.0, 141.4, 144.5, 149.5, 149.8, 153.7 (C-3, C-7, C-4a, C-1’, C-1”, C-4”); 124.8 (C-4’); 130.7 (C-6); 124.0 (C-2”, C-6’); 130.0 (C-3”, C-5”); 137.3 (C-5’); 140.7 (C-3’); 150.0 (C-1); 163.4 (CO); 183.2 (COAr); IR (KBr, cm^{-1}): 1709, 1625, 1597, 1524, 1467, 1346, 1330, 1207.

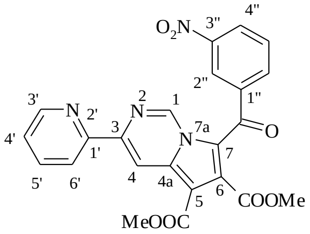

Dimethyl 3-(2-pyridyl)-7-(3-nitrobenzoyl)pyrrolo[1,2-*c*]pyrimidine-5,6-dicarboxylate (12h). Pale yellow powder with mp 246-248 ^oC. Yield 45%. Calcd. $C_{23}H_{16}N_4O_7$: C 60.00, H 3.50, N 12.15. Found C 60.21, H 3.77, N 12.39; ^{1}H-NMR ($CDCl_3$, 300 MHz, δ): 3.40, 3.97 (2 CH_3); 7.40 (ddd, *J* = 7.7, 4.7, 1.1 Hz, 1H, H-4’) 7.71 (t, *J* = 8.0, 1H, H-5”); 7.89 (td, *J* = 7.7, 1.1 Hz, 1H, H-5’); 8.04-8.07 (m, 1H, H-4”); 8.43-8.47 (m, 2H, H-6’, H-6”); 8.58 (t, *J* = 1.9 Hz, 1H, H-2”); 8.75-8.76 (m, 1H, H-3’); 9.22 (d, *J* = 1.6 Hz, 1H, H-4); 10.39 (d, *J* = 1.6 Hz, 1H, H-1); ^{13}C-NMR ($CDCl_3$, 75 MHz, δ): 52.4, 52.8 (2OMe); 106.8

(C-5); 110.5 (C-4); 122.0 (C-6'); 123.7, 126.6, 129.7, 134.4 (C-2", C-4", C-5", C-6"); 119.7, 134.0, 139.7, 140.1, 147.7, 153.3 (C-6, C-3, C-7, C-4a, C-1', C-1"); 124.9 (C-4'); 137.3 (C-5'); 140.4 (C-3'); 149.9 (C-1); 149.6 (C-3"); 162.6, 164.4 (2CO); 183.6 (COAr); IR (ATR, cm^{-1}): 1738, 1704, 1627, 1518, 1446, 1347, 1212.

---

5. Procedure for obtaining 4-(3-pyridyl)pyrimidinium bromides 13a,b.

4-(3-Pyridyl)pyrimidine **7** (10 mmol) and bromoacetophenones **9** (10 mmol) were stirred in 50 mL acetone under reflux for 8 h and then were left at room temperature overnight. The precipitated bromides **13a,b** were removed by filtration.

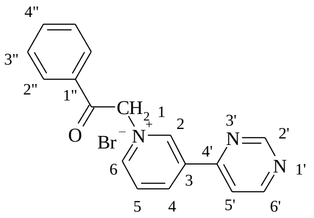

1-[2-Phenyl-2-oxoethyl]-3-(4-pyrimidinyl)pyridinium bromide (13a).

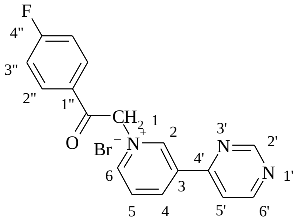

1-[2-(4-Fluorophenyl-2-oxoethyl]-3-(4-pyrimidinyl)pyridinium bromide (13b). Brown powder with mp 218-222 ^{o}C. Yield 78%. Calcd. $C_{17}H_{13}BrFN_3O$: N 11.23. Found N 11.51; ^{1}H-NMR (DMSO, 300 MHz, δ): 6.70 (s, 2H, CH_2); 7.49 (t, 1H, *J* = 8.8 Hz, H-3", H-5"); 8.17-8.22 (m, 2H, H-2", H-6"); 8.40 (dd, 1H, *J* = 5.2, 1.3 Hz, H-5'); 8.59 (dd, 1H, *J* = 8.2, 6.0 Hz, H-5); 9.16 (d, 1H, *J* = 5.2 Hz, H-6'); 9.23 (dt, *J* = 6.0, 1.4 Hz, 1H, H-6); 9.42 (d, 1H, *J* = 1.3 Hz, H-2'); 9.44 (dt, *J* = 8.2, 1.4 Hz, 1H, H-4); 10.00 (m, 1H, H-2); ^{13}C-NMR (DMSO, 75 MHz, δ): 66.5 (CH_2); 116.4 (*J* = 21.7 Hz, C-3", C-5"); 118.6 (C-5'), 128.1 (C-5); 131.4 (*J* = 8.9 Hz, C-2", C-6"); 130.4, 135.6, 157.0 (C-3, C-4', C-1"); 144.0 (C-4); 145.5 (C-2); 147.6 (C-6); 159.0 (C-2'); 159.5 (C-6'); 165.3 (*J* = 252.3 Hz, C-4"); 189.4 (COAr).

---

6. General procedure for obtaining indolizines 14a-f.

4-(3-Pyridyl)pyrimidine **7** (3 mmol), bromoacetophenones **9** (3 mmol) and different acetylenic dipolarophiles **10** (3.5 mmol) were stirred under reflux in 20 mL 1,2-epoxybutane for 48 h. The products were precipitated with ethanol and removed by filtration. Further purification was effected by crystallization from ethanol or column chromatography on Silicagel-60 (Merck, 70-230 mesh) using methylene chloride as eluent.

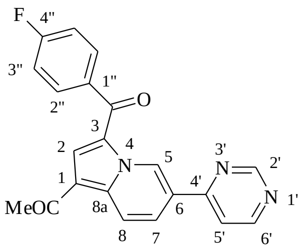

1-Acetyl-3-(4-fluorobenzoyl)-6-(4-pyrimidinyl)indolizine (14a).

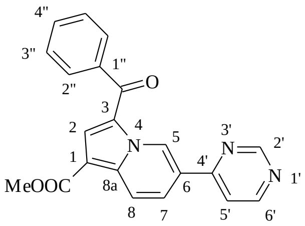

Methyl 3-benzoyl-6-(4-pyrimidinyl)indolizine-1-carboxylate (14b). Pale yellow crystals with mp 199-200 ^{o}C. Yield 68%. Calcd. $C_{21}H_{15}N_3O_2$: C 73.89, H 4.43, N 12.31. Found C 74.23, H 4.76, N 12.58; ^{1}H-NMR ($CDCl_3$, 300 MHz, δ): 3.93 (s, 3H, CH_3); 7.51-7.65 (m, 3H, H-3", H-4", H-5"); 7.82-7.88 (m, 3H, H-6', H-2", H-6"); 7.90 (s, 1H, H-2); 8.21 (dd, 1H, *J* = 9.3, 1.6 Hz, H-7); 8.50 (dd, 1H, *J* = 9.3, 1.1 Hz, H-8); 8.84 (d, *J* = 5.2, 1H, H-5'); 9.32 (d, 1H, *J* = 1.4 Hz, H-2'); 10.76-10.77 (m, 1H, H-5); ^{13}C-NMR ($CDCl_3$, 75 MHz, δ): 51.6 (CH_3); 106.8 (C-1); 116.8 (C-5'); 120.2 (C-8); 125.8 (C-7); 128.6 (C-2", C-6"); 128.7 (C-5); 129.9 (C-3", C-5"); 130.1 (C-2); 132.0 (C-4"); 120.3, 125.4, 130.6, 139.6, , 161.0 (C-3, C-8a, C-6, C-4', C-1"); 157.9 (C-6'); 159.4 (C-2'); 164.3 (CO); 185.9 (COAr); IR (ATR, cm^{-1}): 1711, 1620, 1586, 1523, 1475, 1448, 1359, 1219.

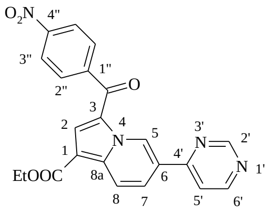

Ethyl 3-(4-nitrobenzoyl)-6-(4-pyrimidinyl)indolizine-1-carboxylate (14c). Yellow crystals with mp 247-249 ^{o}C. Yield 48%. Calcd. $C_{22}H_{16}N_4O_5$: C 63.46, H 3.87, N 13.46. Found C 63.71, H 4.11, N 13.77; ^{1}H-NMR ($CDCl_3$, 300 MHz, δ): 1.43 (t, 3H, *J* = 7.1 Hz, CH_3); 4.42 (q, 2H, *J* = 7.1 Hz, CH_2); 7.82 (s, 1H, H-2); 7.86 (dd, 1H, *J* = 5.2, 1.4 Hz, H-6'); 8.00 (d, 2H, *J* = 8.8 Hz, H-2", H-6"); 8.26 (dd, 1H, *J* = 9.3, 1.6 Hz, H-7); 8.41 (d, 2H, *J* = 8.8 Hz, H-3", H-5"); 8.55(dd, 1H, *J* = 9.3, 1.1 Hz, H-8); 8.88 (d, *J* = 5.2, 1H, H-5'); 9.34 (d, 1H, *J* = 1.4 Hz, H-2'); 10.80 (m, 1H, H-5); ^{13}C-NMR ($CDCl_3$, 75 MHz, δ): 14.7 (CH_3); 60.8 (CH_2); 108.1 (C-1); 116.8 (C-5'); 120.0 (C-8); 124.0 (C-3", C-5"); 126.6 (C-7); 129.2 (C-5); 129.9 (C-2", C-6"); 130.2 (C-2); 122.7, 126.0, 140.5, 145.0, 149.7, 160.7 (C-3, C-8a, C-6, C-4', C-1", C-4"); 158.1 (C-6'); 159.5 (C-2'); 163.6 (CO); 183.4 (COAr); IR (KBr, cm^{-1}): 1701, 1612, 1598, 1523, 1345, 1215, 1173.

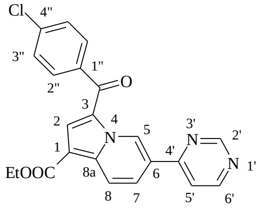

Ethyl 3-(4-chlorobenzoyl)-6-(4-pyrimidinyl)indolizine-1-carboxylate (14d). Yellow crystals with mp 212-214 ^{o}C. Yield 54%. Calcd. $C_{22}H_{16}ClN_3O_3$: C 65.11, H 3.97, Cl 8.74, N 10.35. Found C 65.29, H 4.22, Cl 9.02, N 10.47; ^{1}H-NMR ($CDCl_3$, 300 MHz, δ): 1.41 (t, 3H, *J* = 7.1 Hz, CH_3); 4.39 (q, 2H, *J* = 7.1 Hz, CH_2); 7.50 (d, 2H, *J* = 8.2 Hz, H-2", H-6"); 7.74-7.83 (m, 4H, H-2, H-6', H-3", H-5"); 8.18 (dd, 1H, *J* = 9.3, 1.6 Hz, H-7); 8.48(dd, 1H, *J* = 9.3, 1.1 Hz, H-8); 8.82 (d, *J* = 5.2, 1H, H-5'); 9.29 (d, 1H, *J* = 1.4 Hz, H-2'); 10.70 (br s, 1H, H-5); ^{13}C-NMR ($CDCl_3$, 75 MHz, δ): 14.6 (CH_3); 60.6 (CH_2); 107.3 (C-1); 116.7 (C-5'); 119.8 (C-8); 125.9 (C-7); 128.9 (C-3", C-5"); 129.0 (C-5); 129.7 (C-2); 130.0 (C-2", C-6"); 123.0, 125.5, 137.8, 138.3, 140.0, 160.8 (C-3, C-8a, C-6, C-4', C-1", C-4"); 157.9 (C-6'); 159.4 (C-2'); 163.7 (CO); 184.4 (COAr); IR (ATR, cm^{-1}): 1693, 1630, 1581, 1361, 1219.

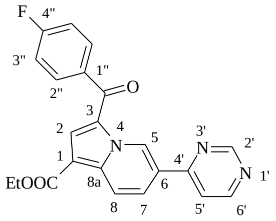

Ethyl 3-(4-Fluorobenzoyl)-6-(4-pyrimidinyl)indolizine-1-carboxylate (14e). Yellow crystals with mp 176-178 ^oC. Yield 60%. Calcd. $C_{22}H_{16}FN_3O_3$: C 67.86, H 4.14, N 10.79. Found C 67.61, H 4.39, N 11.06; ^{1}H-NMR ($CDCl_3$, 300 MHz, δ): 1.42 (t, 3H, *J* = 7.1 Hz, CH_3); 4.41 (q, 2H, *J* = 7.1 Hz, CH_2); 7.23 (t, 1H, *J* = 8.8 Hz, H-2", H-6"); 7.82 (dd, 1H, *J* = 5.2, 1.4 Hz, H-6'); 7.84 (s, 1H, H-2); 7.86-7.91 (m, 2H, H-2", H-6"); 8.17 (dd, 1H, *J* = 9.3, 1.6 Hz, H-7); 8.48(dd, 1H, *J* = 9.3, 1.1 Hz, H-8); 8.83 (d, *J* = 5.2, 1H, H-5'); 9.30 (d, 1H, *J* = 1.4 Hz, H-2'); 10.69-10.70 (m, 1H, H-5); ^{13}C-NMR ($CDCl_3$, 75 MHz, δ): 14.7 (CH_3); 60.6 (CH_2); 115.8 (*J* = 21.7 Hz, C-3", C-5"); 107.2 (C-1); 116.7 (C-5'); 119.8 (C-8); 125.7 (C-7); 129.0 (C-5); 129.6 (C-2); 131.6 (*J* = 9.0 Hz, C-2", C-6"); 123.1, 125.4, 135.8, 139.9, 160.9 (C-3, C-8a, C-6, C-4', C-1"); 157.9 (C-6'); 159.4 (C-2'); 163.8 (CO); 165.1 (*J* = 252.3 Hz, C-4"); 184.3 (COAr); IR (KBr, cm^{-1}); 1701, 1616, 1579, 1463, 1359, 1213, 1156.

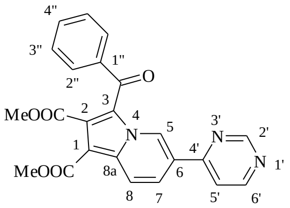

Dimethyl 3-benzoyl-6-(4-pyrimidinyl)indolizine-1,2-dicarboxylate (14f). Orange crystals with mp 133-136 ^oC. Yield 45%. Calcd. $C_{23}H_{17}N_3O_5$: C 66.50, H 4.12, N 10.12. Found : C 66.82, H 4.29, N 10.41; ^{1}H-NMR ($CDCl_3$, 300 MHz, δ): 3.34, 3.98 (2s, 6H, $2CH_3$); 7.45-7.50 (m, 2H, H-3", H-5"); 7.53-7.60 (m, 1H, H-4"); 7.71-8.12 (m, 2H, H-2", H-6"); 7.81 (dd, 1H, *J* = 5.2, 1.4 Hz, H-6'); 8.17 (dd, 1H, *J* = 9.3, 1.6 Hz, H-7); 8.48(dd, 1H, *J* = 9.3, 1.1 Hz, H-8); 8.84 (d, *J* = 5.2, 1H, H-5'); 9.30 (d, 1H, *J* = 1.4 Hz, H-2'); 10.39-10.40 (m, 1H, H-5); ^{13}C-NMR ($CDCl_3$, 75 MHz, δ): 51.9, 52.4 ($2CH_3$); 104.8 (C-1); 116.8 (C-5'); 120.3 (C-8); 125.9 (C-7); 128.3 (C-2", C-6"); 128.8 (C-3", C-5"); 128.5 (C-5); 132.4 (C-4"); 121.8, 131.0, 132.3, 138.3, 139.3, 160.4 (C-2, C-3, C-8a, C-6, C-4', C-1"); 158.0 (C-6'); 159.4 (C-2'); 163.2, 164.9 (2CO); 187.0 (COAr); IR (ATR, cm^{-1}): 1725 (with sh), 1615, 1579, 1492, 1385, 1221.

7. General procedure for obtaining the 4-(4-pyrimidinyl)pyridinium bromides 15.

4-(4-Pyridyl)pyrimidine **8** (10 mmol) and different bromoacetophenones **9** (10 mmol) were stirred in 50 mL acetone under reflux for 8 h and then were left at room temperature overnight. The precipitated bromides **15a-b** were removed by filtration.

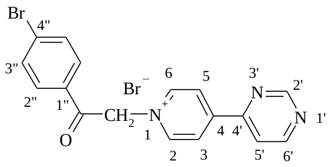

1-[2-(4-Bromophenyl)-2-oxoethyl]-4-(4-pyrimidinyl)pyridinium bromide (15a).

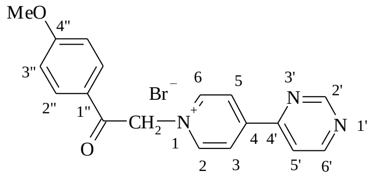

1-[2-(4-Methoxyphenyl)-2-oxoethyl]-4-(4-pyrimidinyl)pyridinium bromide (15b). Brown powder with mp 238-240 ^{o}C. Yield 88%. Calcd. $C_{18}H_{16}BrN_3O_2$: N 10.88. Found N 11.19; ^{1}H-NMR (DMSO, 300 MHz, δ): 3.90 (OMe); 6.60 (s, 2H, CH_2); 7.19 (d, *J* = 8.7 Hz, 2H, H-3", H-5"); 8.05 (d, *J* = 8.7 Hz, 2H, H-2", H-6"); 8.55 (dd, *J* = 5.3, 1.3 Hz, H-5'); 9.00 (d, *J* = 6.9 Hz, 2H, H-3, H-5); 9.21 (d, *J* = 5.3 Hz, 1H, H-6'); 9.25 (d, *J* = 6.9 Hz, 2H, H-2, H-6); 9.53 (d, *J* = 1.3 Hz , 1H, H-2'); ^{13}C-NMR (DMSO, 75 MHz, δ): 55.9 (OMe); 66.0 (CH_2); 114.5 (C-3", C-5"); 119.9 (C-5'); 125.0 (C-3, C-5); 126.4, 151.4, 157.1, 164.4 (C-4, C-1', C-1", C-4"); 130.9 (C-2", C-6"); 147.3 (C-2, C-6); 159.3 (C-6'); 160.0 (C-2'); 188.9 (COAr).

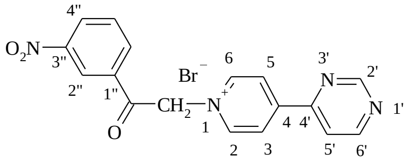

8. General procedure for obtaining the indolizines 16a-f.

4-(4-Pyridyl)pyrimidine **8** (3 mmol), bromoacetophenone **9** (3 mmol) acetylenic dipolarophile **10** (3.5 mmol) in 20 mL 1,2-epoxybutane were stirred under reflux for 48 h. The products were precipitated with ethanol and removed by filtration. Further

purification was performed by crystallization from ethanol or column chromatography on Silicagel-60 (Merck) or neutral Al_2O_3 using methylene chloride as eluent.

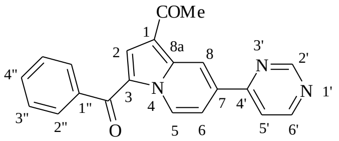

1-Acetyl-3-benzoyl-7-(4-pyrimidinyl)indolizine (16a).

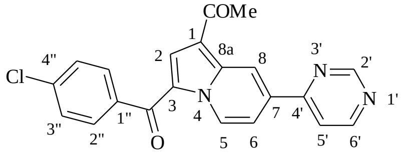

1-Acetyl-3-(4-chlorobenzoyl)-7-(4-pyrimidinyl)indolizine (16b). Yellow crystals with mp 287-289 ^oC. Yield 56%. Calcd. $C_{21}H_{14}ClN_3O_2$: C 67.12, H 3.75, Cl 9.43, N 11.18. Found C 67.45, H 3.57, Cl 9.76, N 11.36; ^{1}H-NMR ($CDCl_3$, 300 MHz, δ): 2.57 (s, 3H, Me); 7.54 (d, *J* = 8.5 Hz, 2H, H-3”, H-5”); 7.72 (s, 1H, H-2); 7.81(d, *J* = 8.5 Hz, 2H, H-2”, H-6”); 7.97 (dd, *J* = 5.3, 1.4 Hz, 1H, H-5’); 8.03 (dd, *J* = 7.4, 1.9 Hz, 1H, H-6); 8.90 (d, *J* = 5.4 Hz, 1H, H-6’); 9.32 (dd, *J* = 1.9, 0.8 Hz, 2H, H-8); 9.36 (d, *J* = 1.4 Hz, 1H, H-2’); 10.00 (dd, *J* = 7.4, 0.8 Hz, 1H, H-5); ^{13}C-NMR ($CDCl_3$, 75 MHz, δ): 28.1 (Me); 114.2 (C-6); 116.5 (C-1); 117.4 (C-5’); 119.0 (C-8); 122.9, 136.7, 137.9, 138.4, 139.0, 160.8 (C-3, C-8a, C-7, C-1’, C-1”, C-4”); 129.2 (C-5); 130.2 (C-2); 129.0, 130.5 (C-2”, C-6”, C-3”, C-5”); 158.2 (C-6’); 159.4 (C-2’); 184.4 (COAr); 193.3 (COMe); IR (KBr, cm^{-1}): 1651, 1610, 1577, 1504, 1478, 1422, 1340, 1227, 1191.

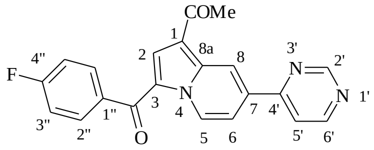

1-Acetyl-3-(4-fluorobenzoyl)-7-(4-pyrimidinyl)indolizine (16c). Yellow crystals with mp 240-242 ^oC. Yield 62%. Calcd. $C_{21}H_{14}FN_3O_2$: C 70.19, H 3.93, N 11.69. Found C 70.34, H 4.28, N 11.92; ^{1}H-NMR ($CDCl_3$, 300 MHz, δ): 2.51 (s, 3H, Me); 7.18 (t, *J* = 8.5 Hz, 2H, H-3”, H-5”); 7.65 (s, 1H, H-2); 7.80-7.85 (m, 2H, H-2”, H-6”); 7.91 (d, *J* = 5.4 Hz, 1H, H-5’); 7.94 (dd, *J* = 7.4, 1.9 Hz, 1H, H-6); 8.83 (d, *J* = 5.4 Hz, 1H, H-6’); 9.25-9.29 (m, 2H, H-8, H-2’); 9.91 (d, *J* = 7.4, 0.8 Hz, 1H, H-5); ^{13}C-NMR ($CDCl_3$, 75 MHz, δ): 28.1 (Me); 114.1 (C-6); 115.9 (*J* = 21.8 Hz, C-3”, C-5”); 116.4 (C-1); 117.4 (C-5’); 119.0 (C-8);

123.0, 135.8, 136.6, 138.9, 160.9 (C-3, C-8a, C-7, C-1’, C-1”); 128.9 (C-5); 129.2 (C-2); 131.5 (*J* = 9.2 Hz, C-2”, C-6”); 158.2 (C-6’); 159.4 (C-2’); 165.1 (*J* = 253.4 Hz, C-4”); 184.4 (COAr); 193.4 (COMe); IR (KBr, cm^{-1}): 1650, 1623, 1578, 1519, 1505, 1426, 1342, 1229, 1199.

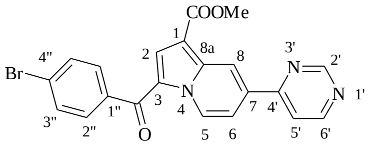

Methyl 3-(4-bromobenzoyl)-7-(4-pyrimidinyl)indolizine-1-carboxylate (16d). Yellow crystals with mp 281-282 °C. Yield 50%. Calcd. $C_{21}H_{14}BrN_3O_3$: C 57.82, H 3.23, Br 18.32, N 9.63. Found C 58.07, H 3.44, Br 18.60, N 9.91; ^{1}H-NMR ($CDCl_3$+TFA, 300 MHz, δ): 4.05 (s, 3H, Me); 7.73 (d, *J* = 8.7 Hz, 2H, H-3”, H-5”); 7.75 (d, *J* = 8.7 Hz, 2H, H-2”, H-6”); 8.00 (s, 1H, H-2); 8.10 (dd, 1H, *J* = 7.4, 2.0 Hz, H-6); 8.68 (dd, *J* = 6.6, 1.1 Hz, 1H, H-5’); 9.28 (d, *J* = 6.6, 1.4 Hz, 1H, H-6’); 9.46 (dd, *J* = 2.0, 0.8 Hz, H-8); 9.66 (t, *J* = 1.1 Hz, H-2’); 10.07 (dd, *J* = 7.4, 0.8 Hz, 1H, H-5); ^{13}C-NMR ($CDCl_3$+TFA, 75 MHz, δ): 53.1 (Me); 110.5 (C-1); 113.7 (C-6); 117.3 (C-5’); 118.1 (C-8); 124.4, 128.7, 132.3, 136.6, 139.4, 168.9 (C-3, C-8a, C-7, C-1’, C-1”, C-4”); 130.5 (C-5); 131.5 (C-2); 130.9 (C-2”, C-6”); 132.5 (C-3”, C-5”); 149.4 (C-6’); 151.8 (C-2’); 165.6 (CO); 187.0 (COAr); IR (ATR, cm^{-1}): 1721, 1610, 1579, 1455, 1352, 1216.

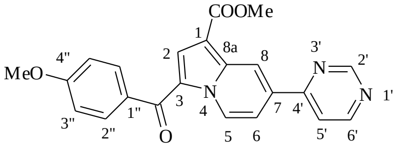

Methyl 3-(4-methoxybenzoyl)-7-(4-pyrimidinyl)indolizine-1-carboxylate (16e). Yellow crystals with mp 249-250 °C. Yield 54%. Calcd. $C_{22}H_{17}N_3O_4$: C 68.21, H 4.42, N 10.85. Found C 68.53, H 4.29, N 11.11; ^{1}H-NMR ($CDCl_3$+TFA, 300 MHz, δ): 3.95 (s, 3H, Me); 4.04 (s, 1H, OMe); 7.09 (d, *J* = 9.0 Hz, 2H, H-3”, H-5”); 7.87 (d, *J* = 9.0 Hz, 2H, H-2”, H-6”); 8.00 (s, 1H, H-2); 8.01 (dd, *J* = 7.4, 1.9 Hz, 1H, H-6); 8.60 (dd, *J* = 6.6, 2.0 Hz, 1H, H-5’); 9.27 (dd, *J* = 6.6, 1.1 Hz, 1H, H-6’); 9.41 (dd, *J* = 1.9, 0.8 Hz, H-8); 9.63 (t, *J* = 1.1 Hz, H-2’); 9.97 (dd, *J* = 7.4, 0.8 Hz, 1H, H-5); ^{13}C-NMR ($CDCl_3$+TFA, 75 MHz, δ): 52.8 (Me); 55.8 (OMe); 110.2 (C-1); 114.5 (C-3”, C-5”); 118.6 (C-5’); 121.1 (C-8); 124.7, 130.2, 131.5, 138.8, 168.7 (C-3, C-8a, C-7, C-4’, C-1”); 130.1 (C-5); 130.7 (C-2); 132.1

(C-2”, C-6”); 149.5 (C-6’); 151.9 (C-2’); 164.1 (C-4”); 165.4 (CO); 186.8 (COAr); IR (ATR, cm^{-1}): 1692, 1603, 1574, 1530, 1458, 1354, 1301, 1262, 1221.

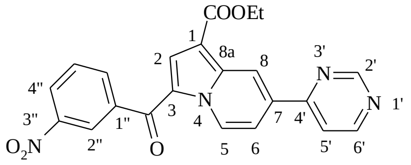

Ethyl 3-(3-nitrobenzoyl)-7-(4-pyrimidinyl)indolizine-1-carboxylate (16f). Yellow crystals with mp 208-210 ^{o}C. Yield 42%. Calcd. $C_{22}H_{16}N_4O_5$: C 63.46, H 3.87, N 13.46. Found C 63.29, H 4.07, N 13.39; ^{1}H-NMR ($CDCl_3$, 300 MHz, δ): 1.43 (t, *J* = 7.1 Hz, 3H, CH_3); 4.43 (q, *J* = 7.1 Hz, 2H, CH_2); 7.76 (t, *J* = 8.0, 1H, H-5”); 7.80 (s, 1H, H-2); 7.92 (dd, *J* = 5.4, 1.4 Hz, 1H, H-5’); 7.93 (dd, *J* = 7.4, 2.0 Hz, 1H, H-6); 8.15-8.19(m, 1H, H-4”); 8.45-8.49 (m, 1H, H-6”); 8.67 (t, *J* = 1.9 Hz, 1H, H-2”); 8.90 (d, *J* = 5.4 Hz, 1H, H-6’); 9.17(dd, *J* = 1.9, 0.8 Hz, H-8); 9.37 (t, *J* = 1.4 Hz, H-2’); 10.02 (d, *J* = 7.4, 0.8 Hz, 1H, H-5); ^{13}C-NMR ($CDCl_3$, 75 MHz, δ): 14.7 (Me); 60.7 (CH_2); 109.1 (C-1); 113.7 (C-6); 117.3 (C-5’); 118.3 (C-8); 123.9, 126.2, 129.9, 134.6 (C-2”, C-4”, C-5”, C-6”); 122.6, 136.0, 139.9, 141.2, 160.8 (C-3, C-8a, C-7, C-1’, C-1”); 129.2 (C-5); 129.5 (C-2); 148.4 (C-3”); 158.2 (C-6’); 159.5 (C-2’); 163.6 (CO); 182.8 (COAr); IR (ATR, cm^{-1}): 1706, 1612, 1579, 1533, 1347, 1204.

---

9. Annexes (Spectral Data)

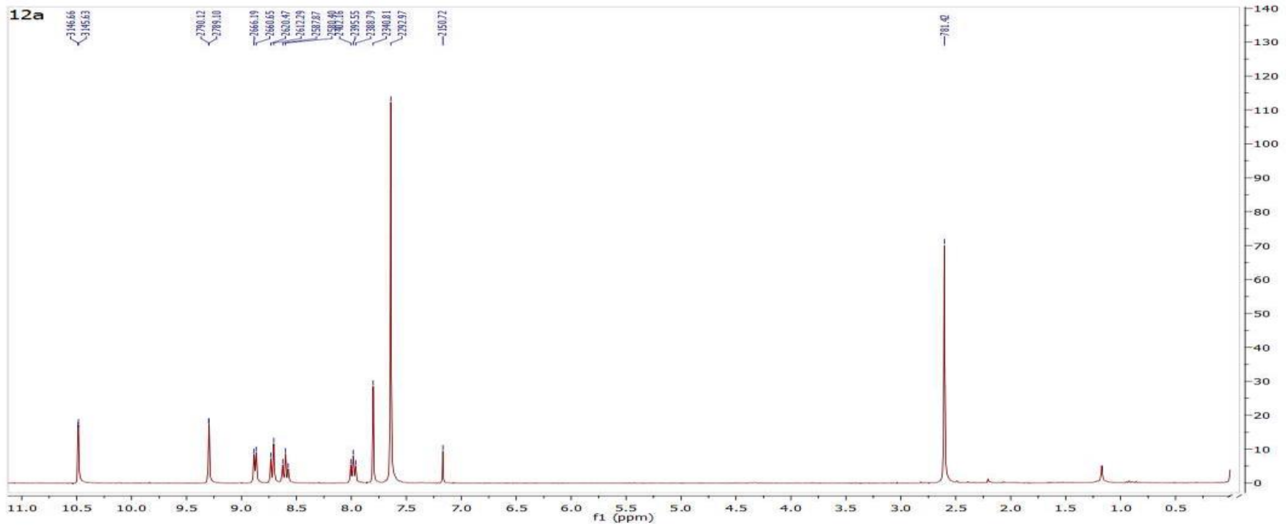

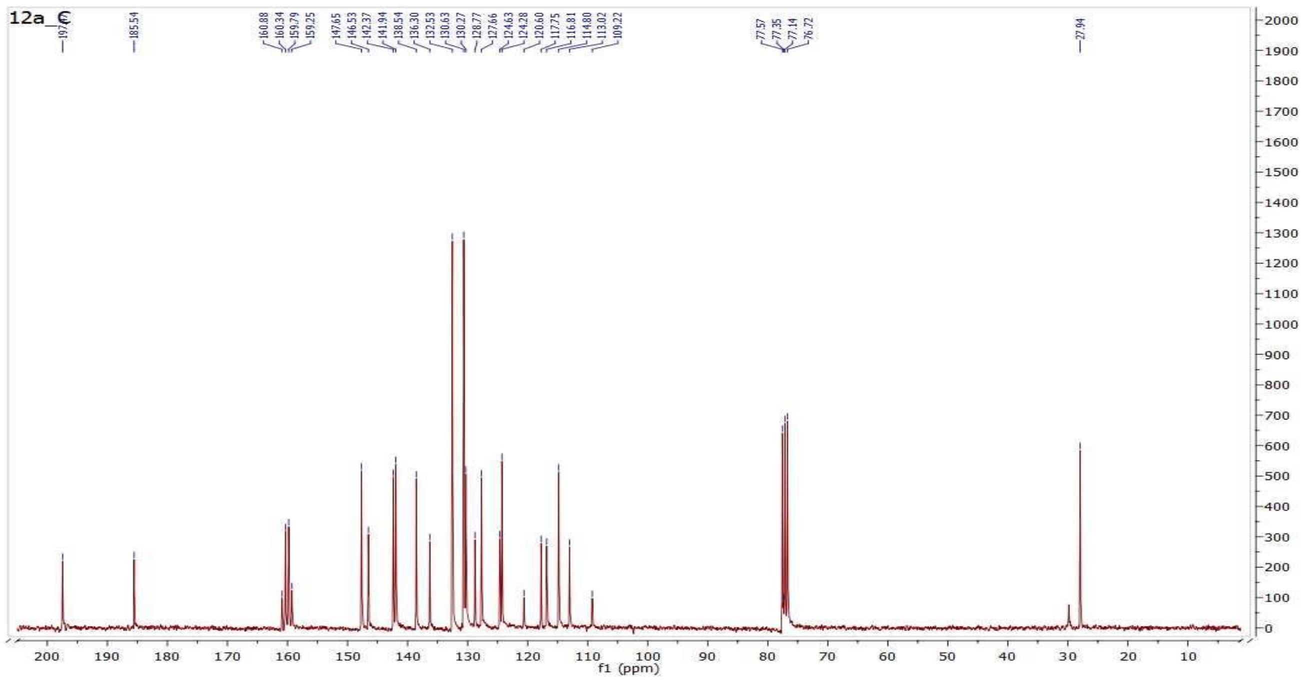
12a_C
f1 (ppm)

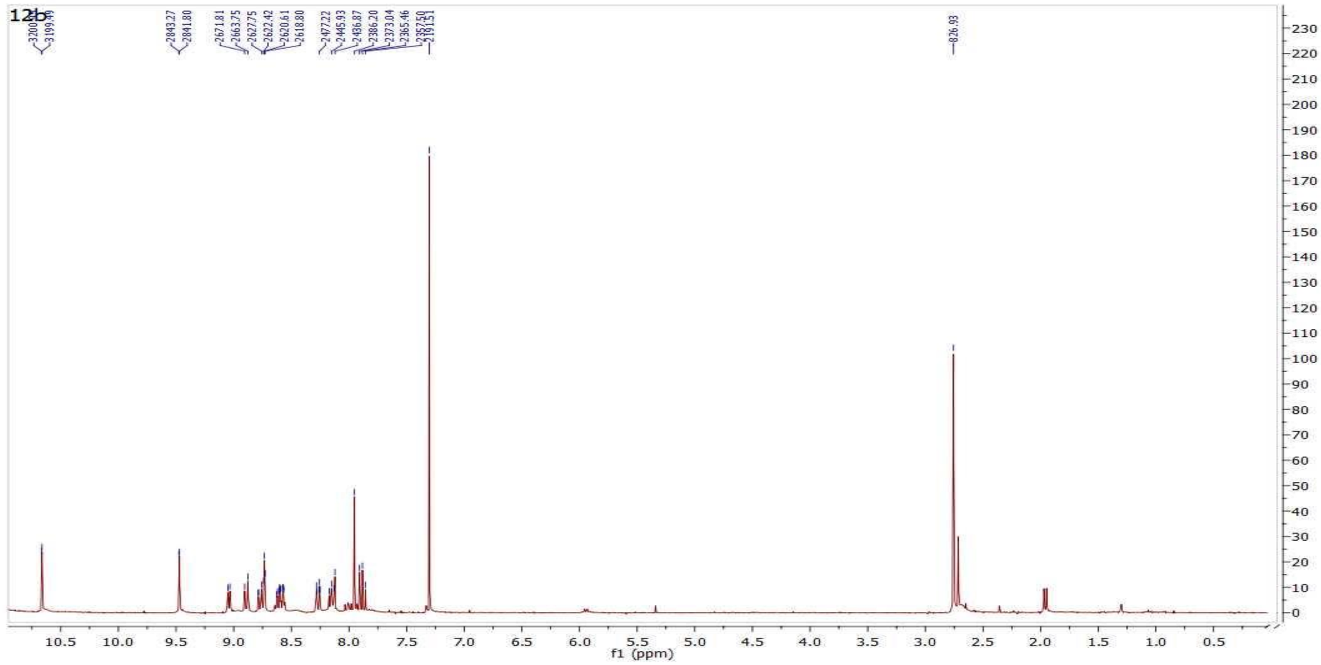
12b
f1 (ppm)

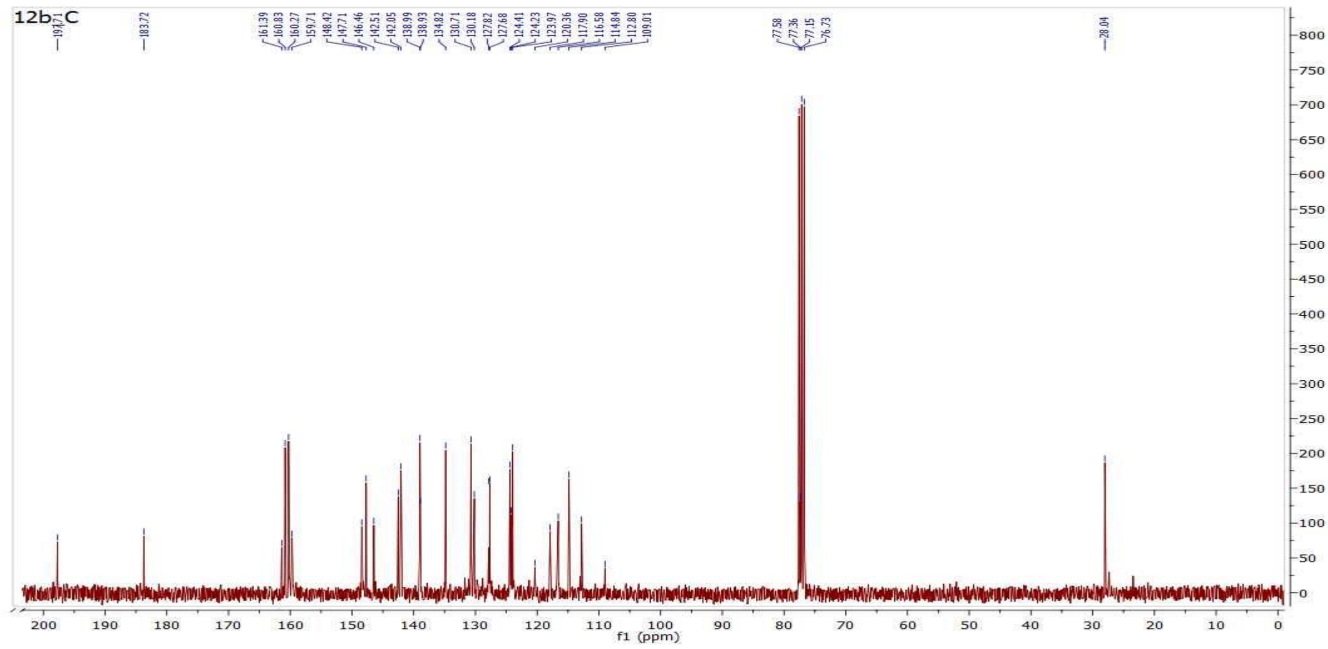
12b C
f1 (ppm)

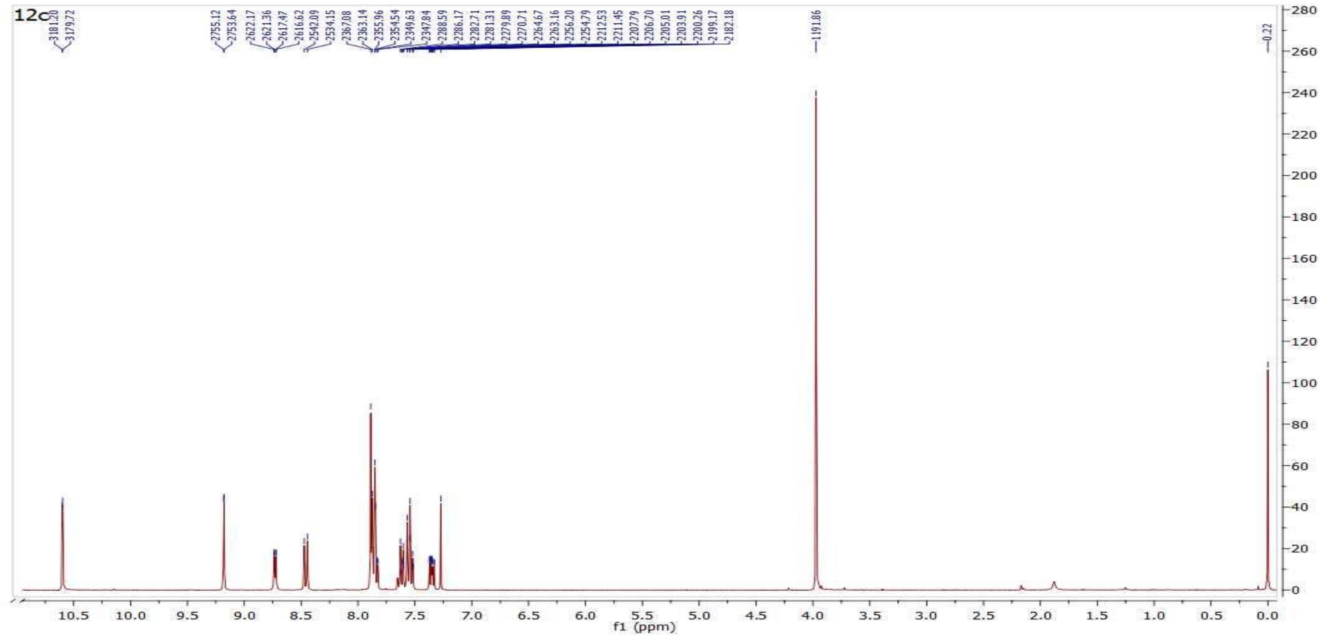
12c
f1 (ppm)

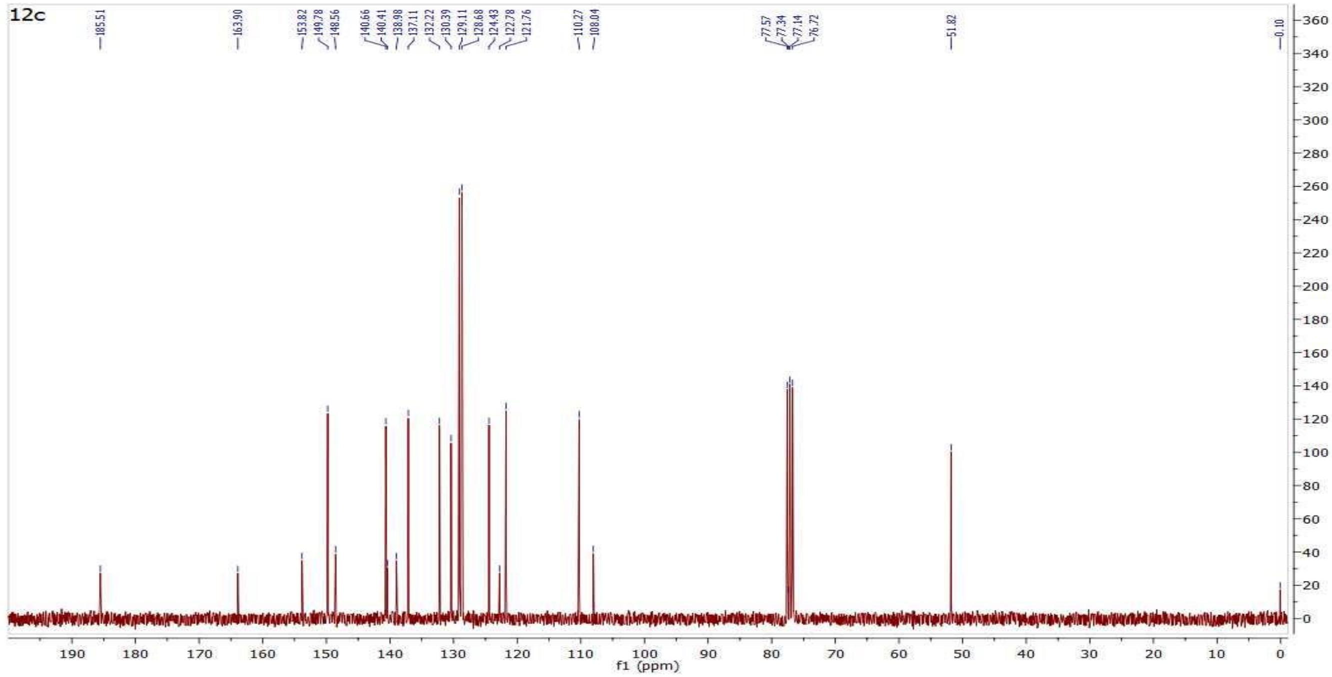
12c
185.51
163.90
153.82
149.78
148.56
140.66
140.41
138.98
137.11
132.22
130.39
129.11
128.68
124.43
122.78
121.76
110.27
108.04
77.57
77.34
77.14
76.72
51.82
0.10
f1 (ppm)

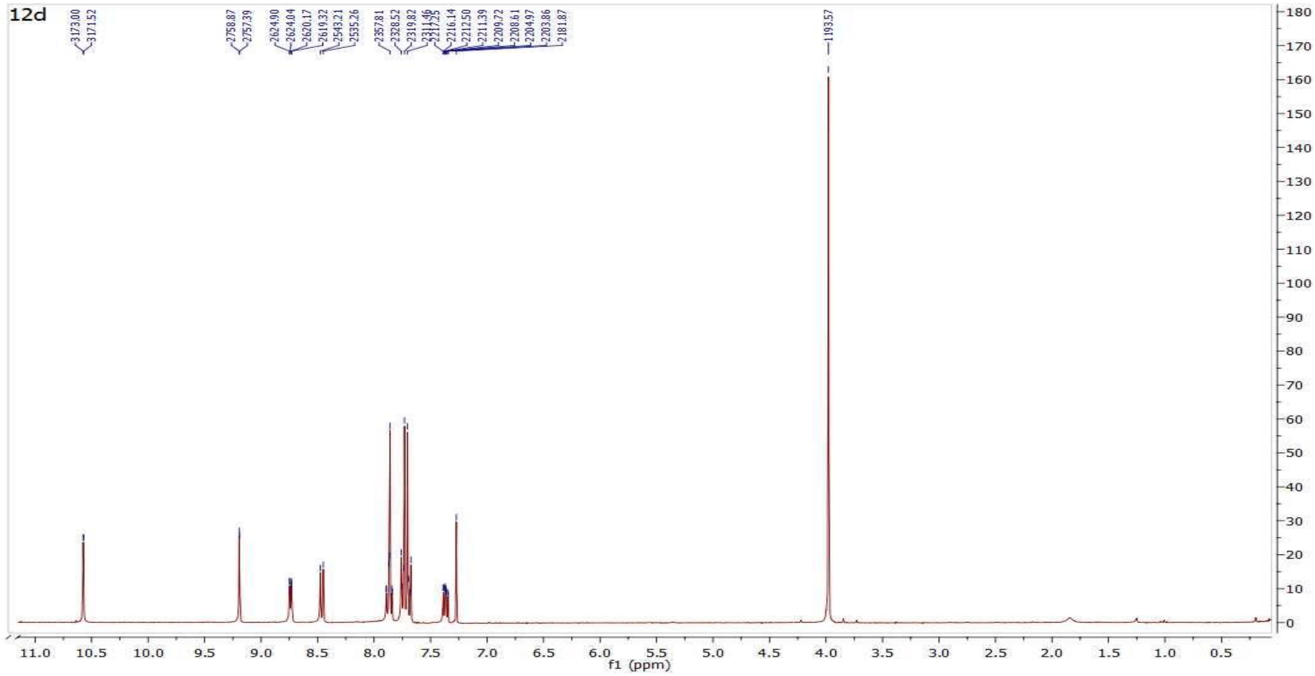
12d
3173.00
3171.52
2758.87
2757.39
2624.90
2624.04
2620.17
2619.32
2543.21
2535.26
2357.81
2328.52
2319.82
2216.14
2212.50
2211.39
2209.72
2208.61
2204.97
2203.86
2181.87
1193.57
f1 (ppm)

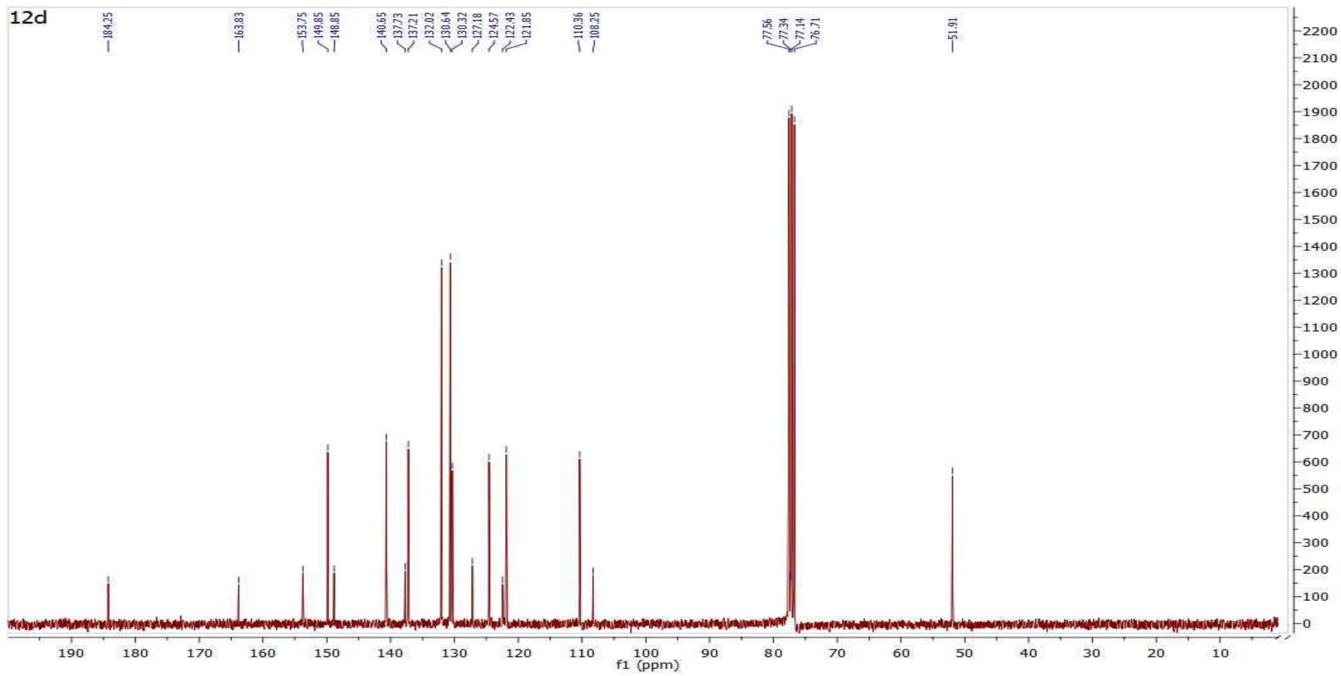
12d
184.25
163.83
153.75
149.85
148.85
140.65
137.73
137.21
132.02
130.64
130.32
127.18
124.57
122.43
121.85
110.36
108.25
77.56
77.34
77.14
76.71
51.91
f1 (ppm)

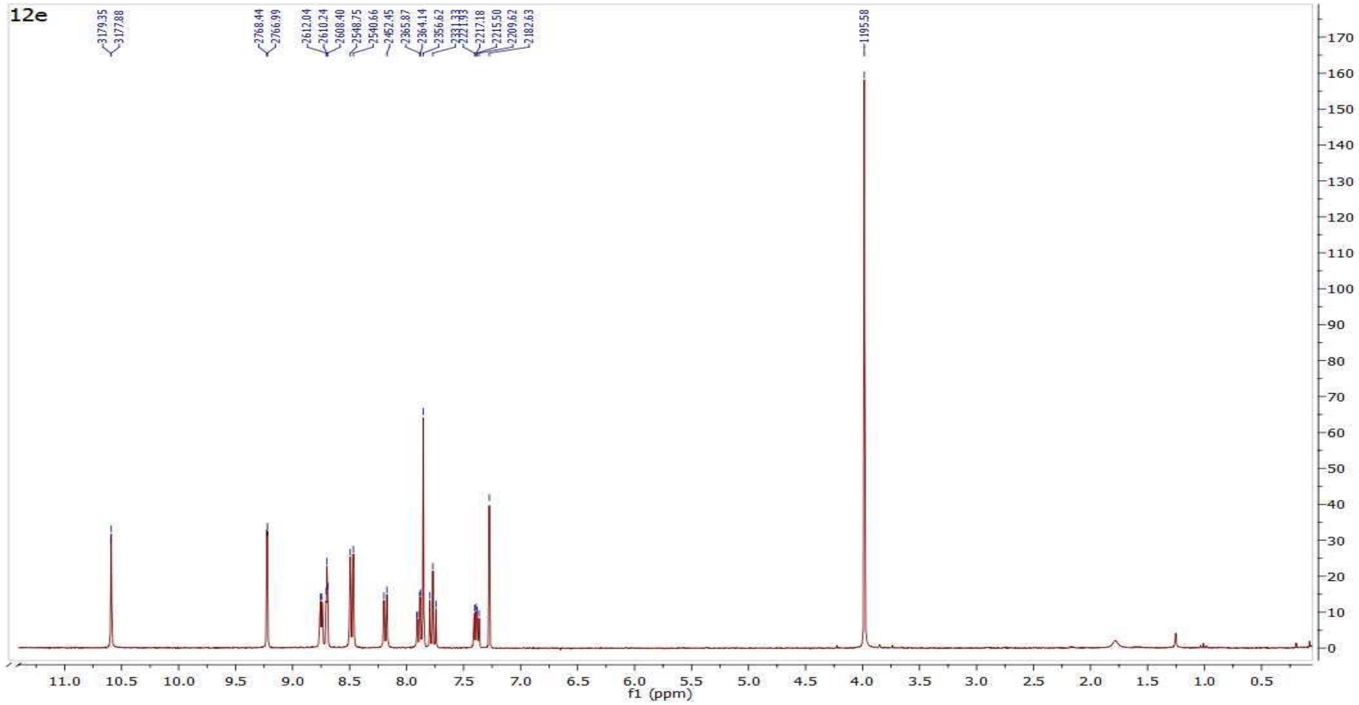
12e
3179.35
3177.88
2768.44
2766.99
2612.04
2610.24
2608.40
2548.75
2540.66
2452.45
2365.87
2364.14
2356.62
2331.33
2221.93
2217.18
2215.50
2209.62
2182.63
1195.58
f1 (ppm)

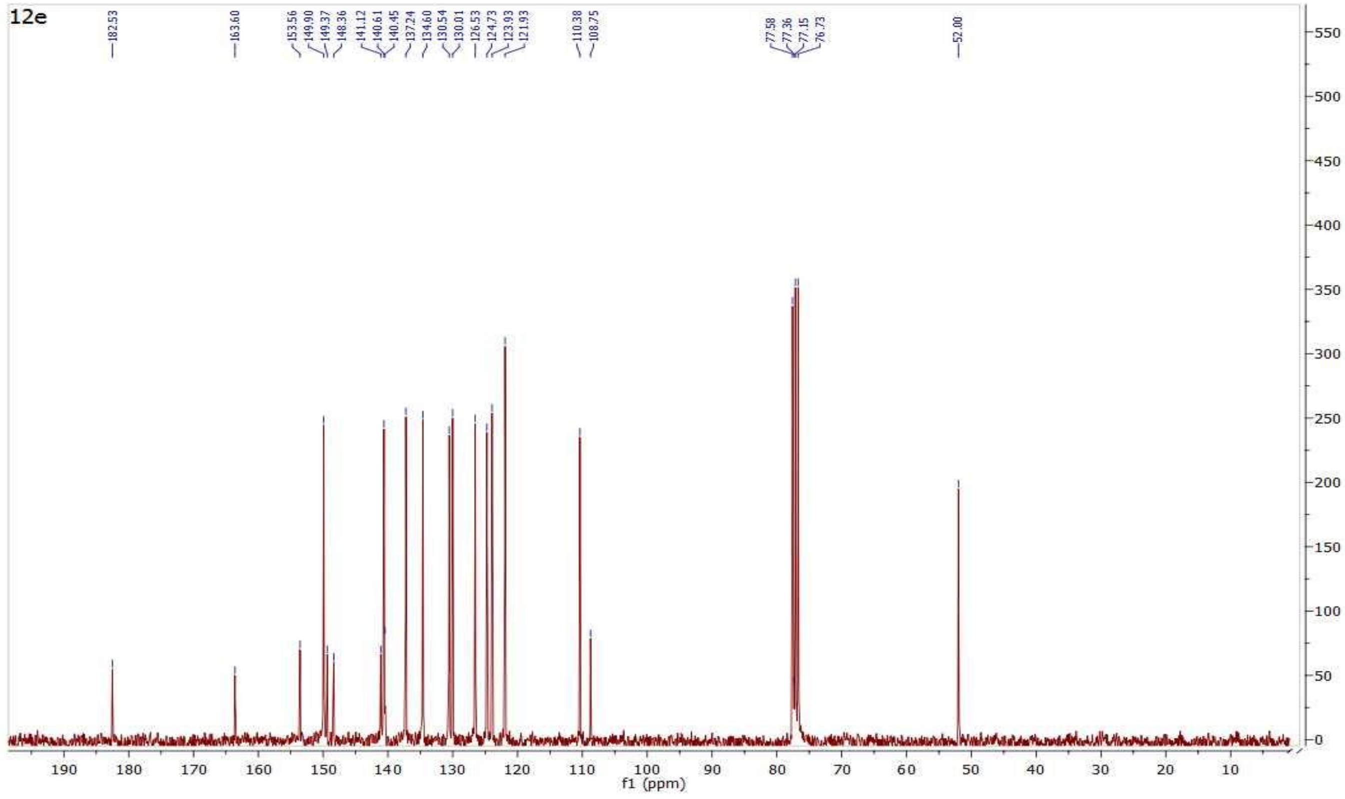
12e
182.53
163.60
153.56
149.90
149.37
148.36
141.12
140.61
140.45
137.24
134.60
130.54
130.01
126.53
124.73
123.93
121.93
110.38
108.75
77.58
77.36
77.15
76.73
52.00
f1 (ppm)

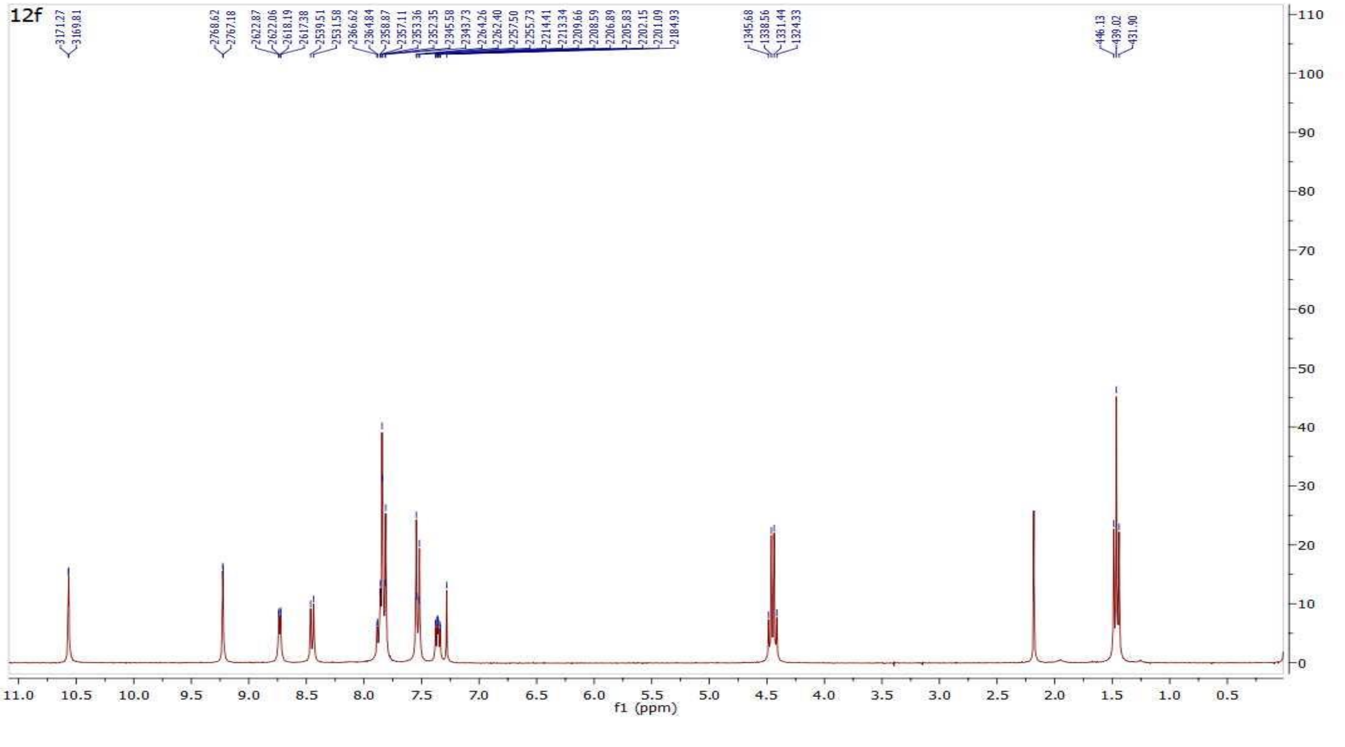
12f
3171.27
3169.81
2768.62
2767.18
2622.87
2622.06
2618.19
2617.38
2539.51
2531.58
2366.62
2364.84
2358.87
2357.11
2353.36
2352.35
2345.58
2343.73
2264.26
2262.40
2257.50
2255.73
2214.41
2213.34
2209.66
2208.59
2206.89
2205.83
2202.15
2201.09
2184.93
1345.68
1338.56
1331.44
1324.33
446.13
439.02
431.90
f1 (ppm)

12f

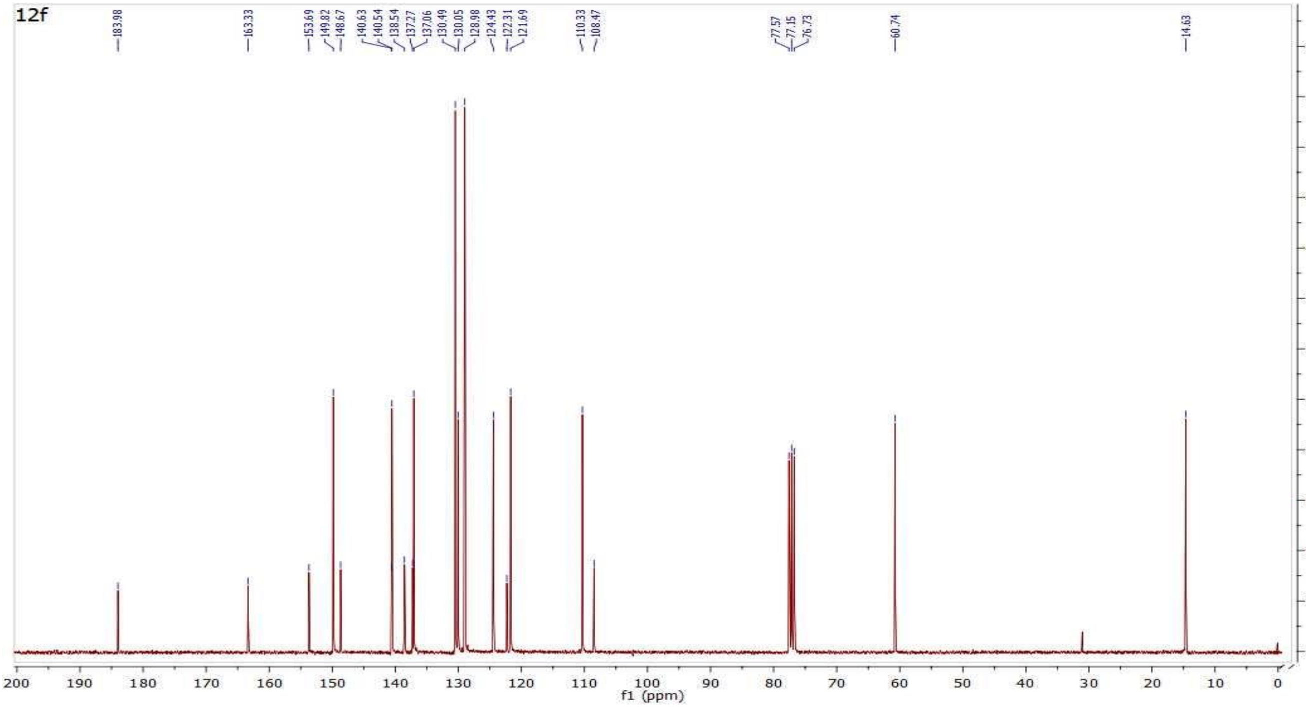

12g

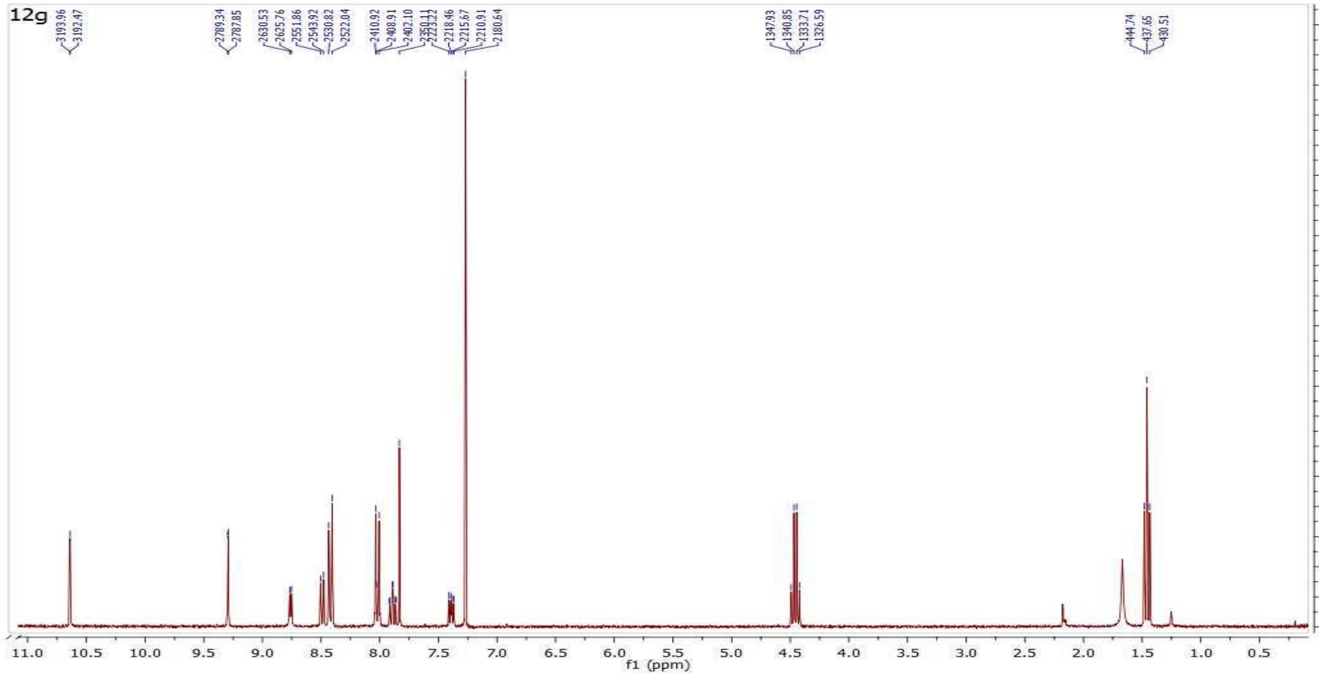

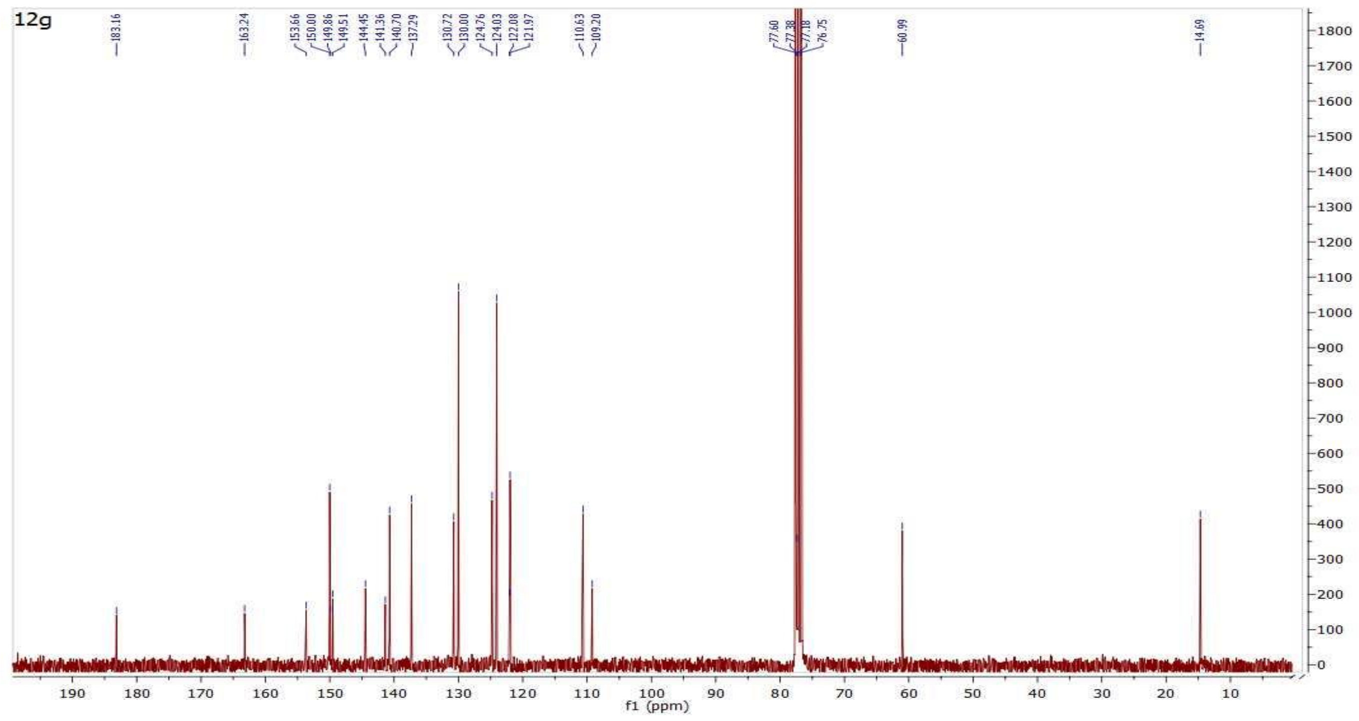
12g
183.16
163.24
153.66
150.00
149.86
149.51
144.45
141.36
140.70
137.29
130.72
130.00
124.76
124.03
122.08
121.97
110.63
109.20
77.60
77.38
77.18
76.75
60.99
14.69
f1 (ppm)

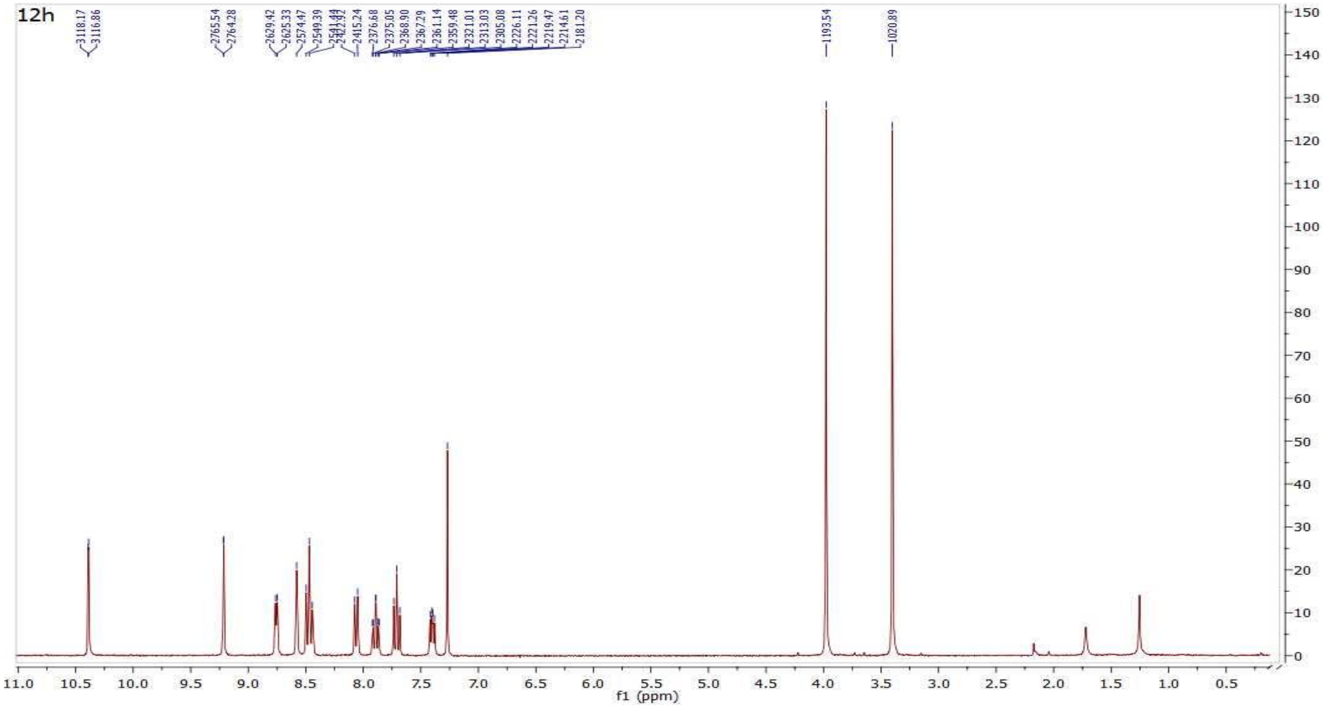
12h
3118.17
3116.86
2765.54
2764.28
1193.54
1020.89
f1 (ppm)

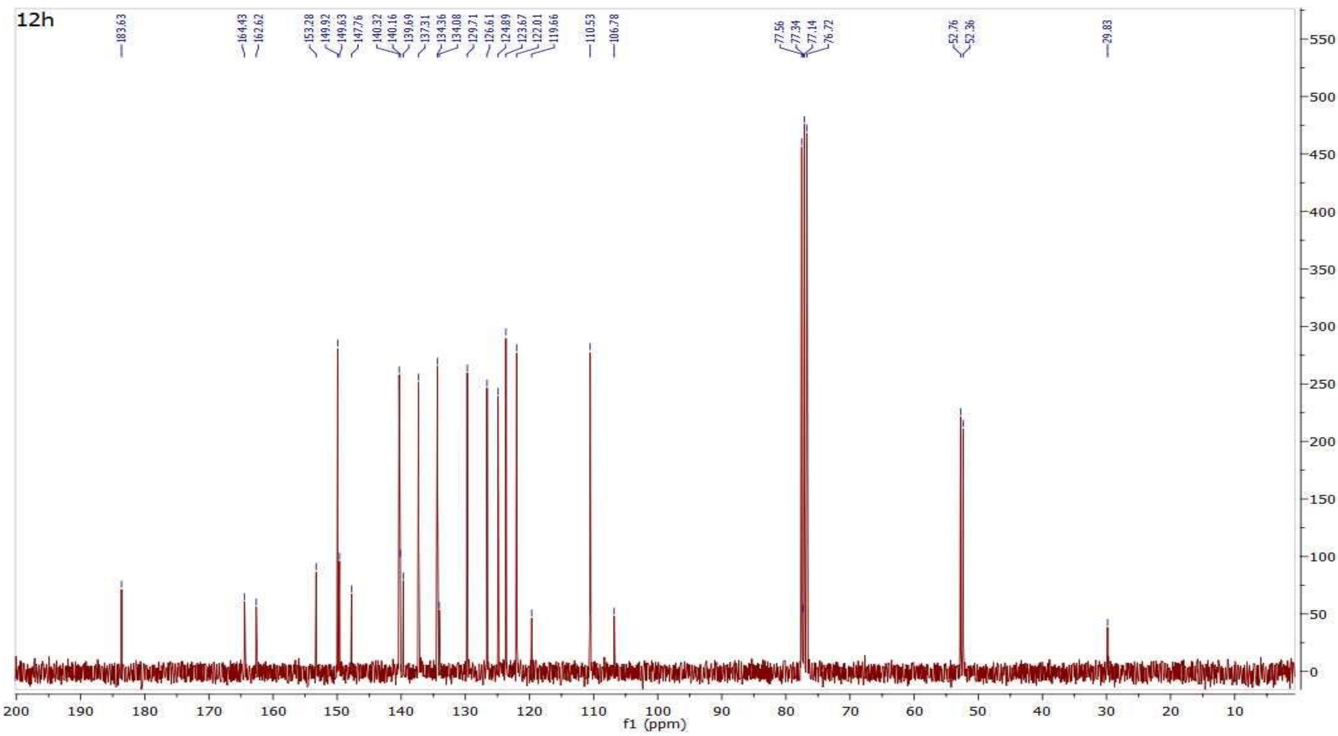

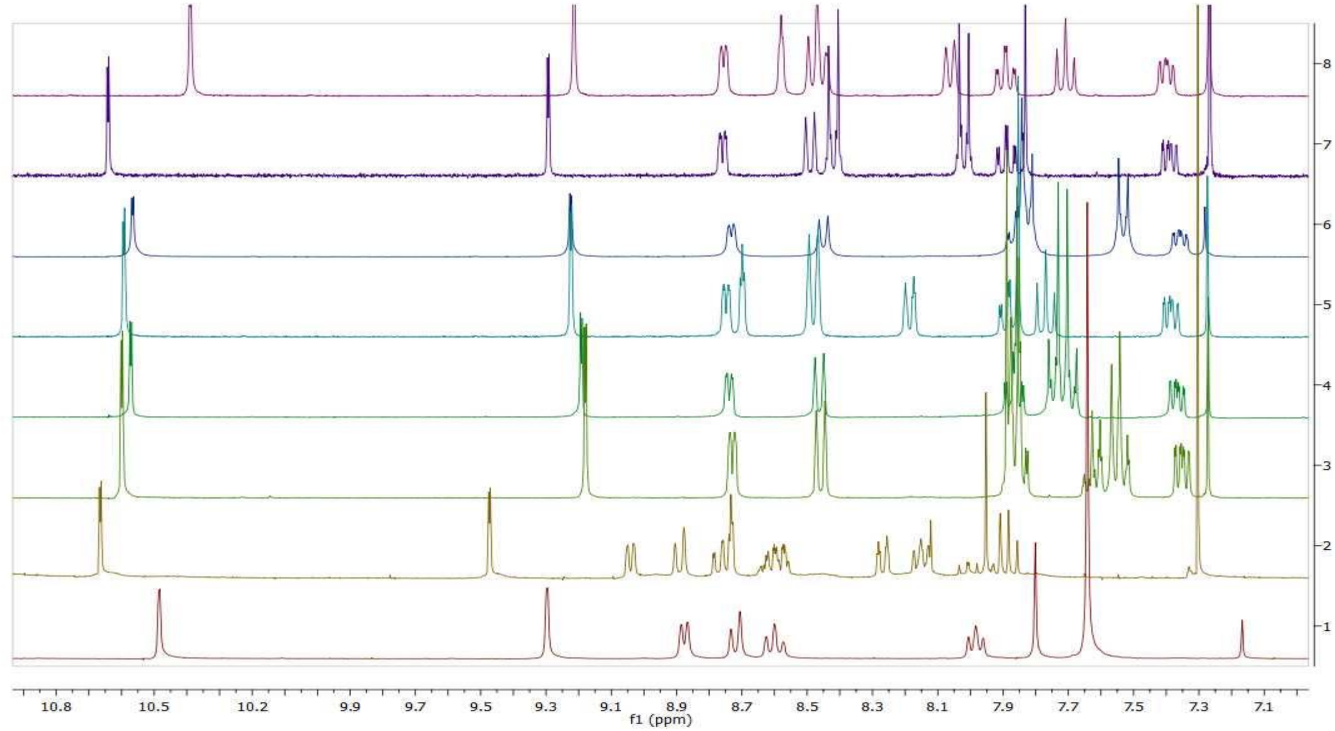

(Superimposed ^{1}H-NMR spectra of compounds **11a-h**-aromatic region)

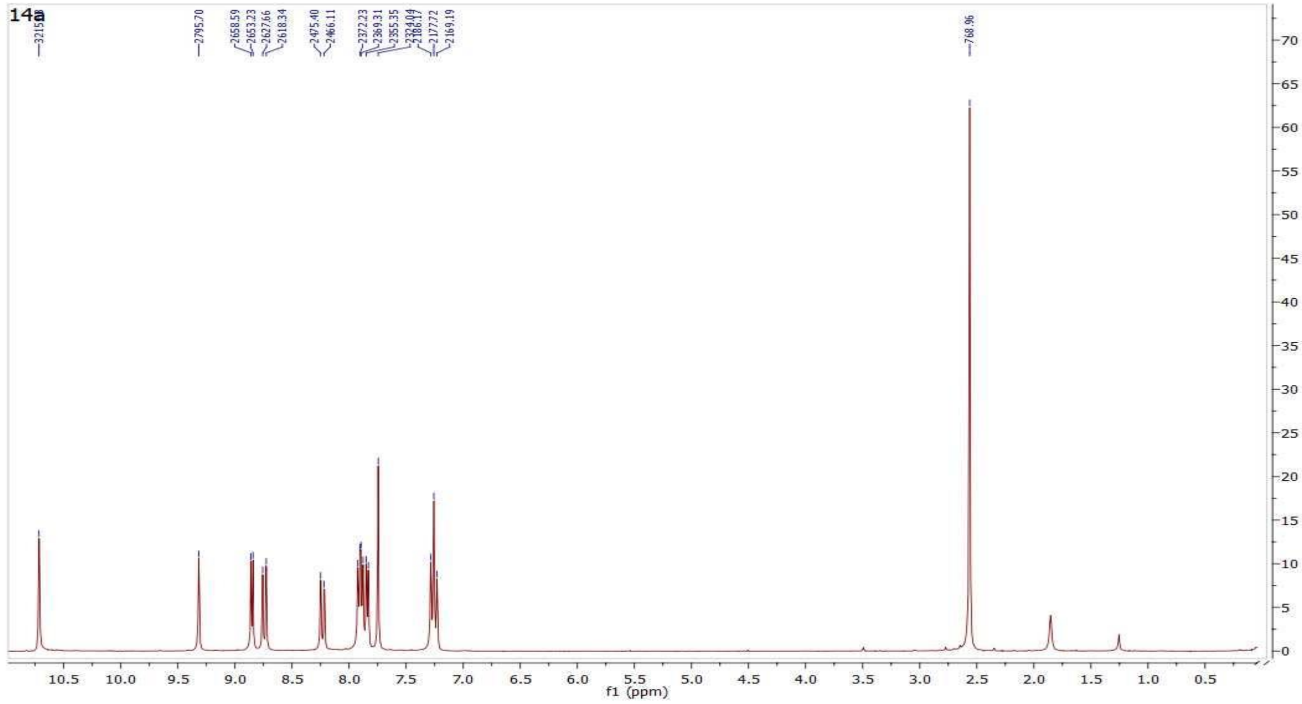
14a
f1 (ppm)

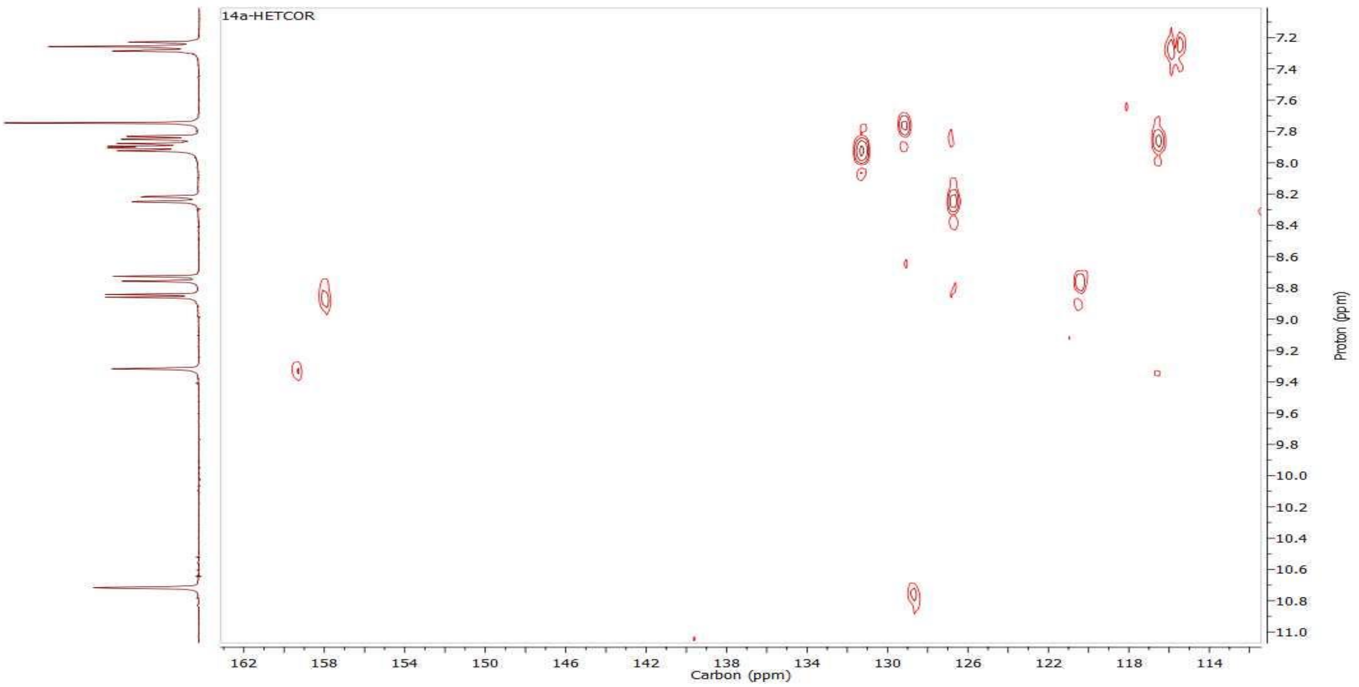
14a-HETCOR
Carbon (ppm)
Proton (ppm)

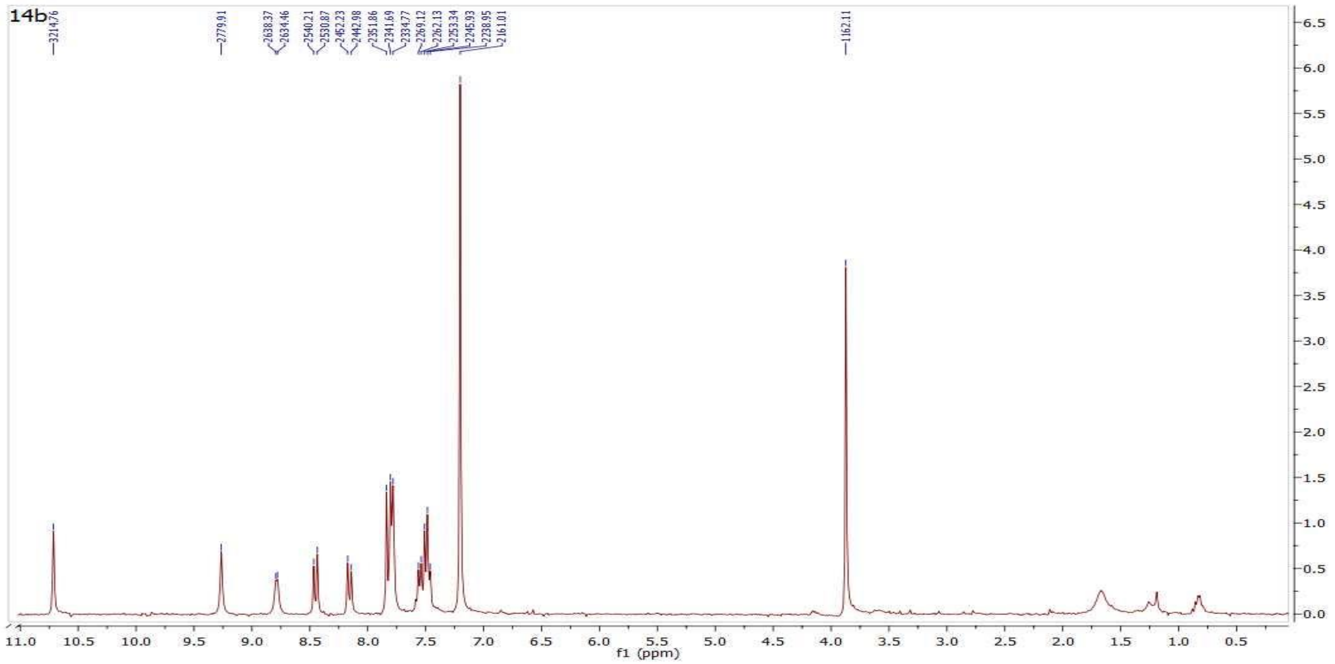
14b
3214.76
2779.91
2638.37
2634.46
2540.21
2530.87
2452.23
2442.98
2351.86
2341.69
2334.77
2269.12
2262.13
2253.34
2245.93
2238.95
2161.01
1162.11
f1 (ppm)

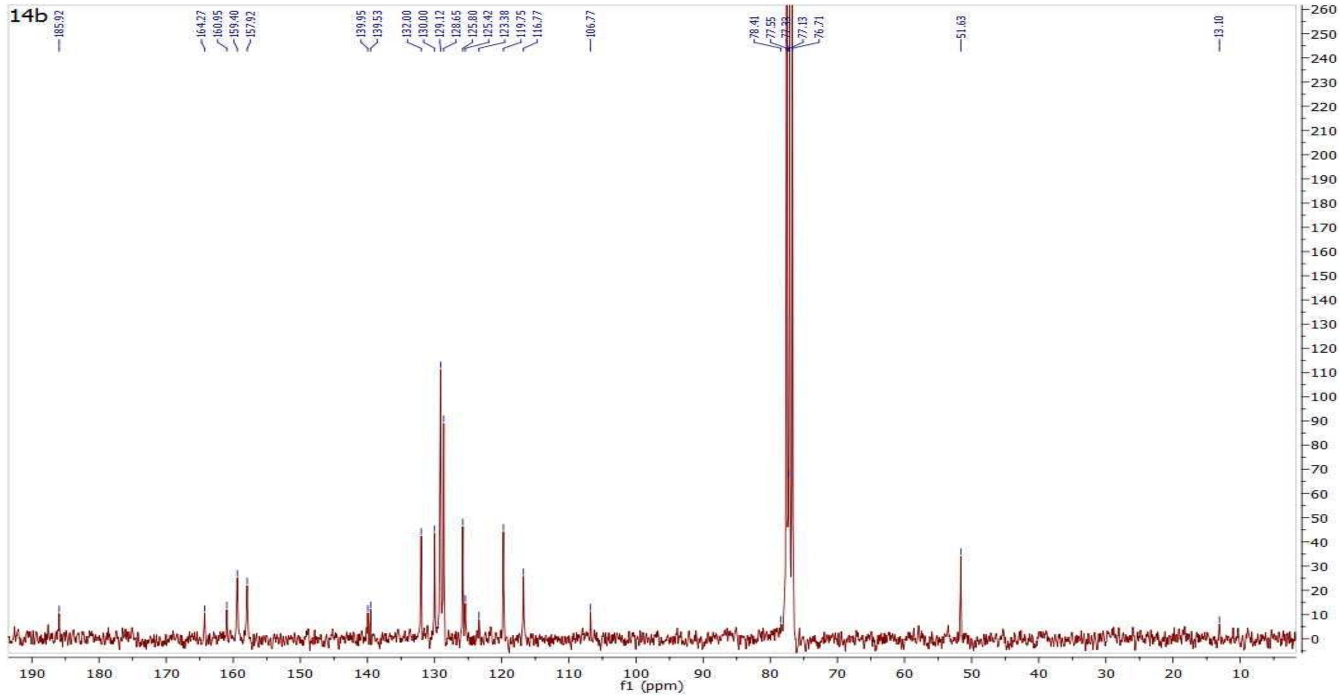
14b
185.92
164.27
160.95
159.40
157.92
139.95
139.53
132.00
130.00
129.12
128.65
125.80
125.42
123.38
119.75
116.77
106.77
78.41
77.55
77.32
77.13
76.71
51.63
13.10
f1 (ppm)

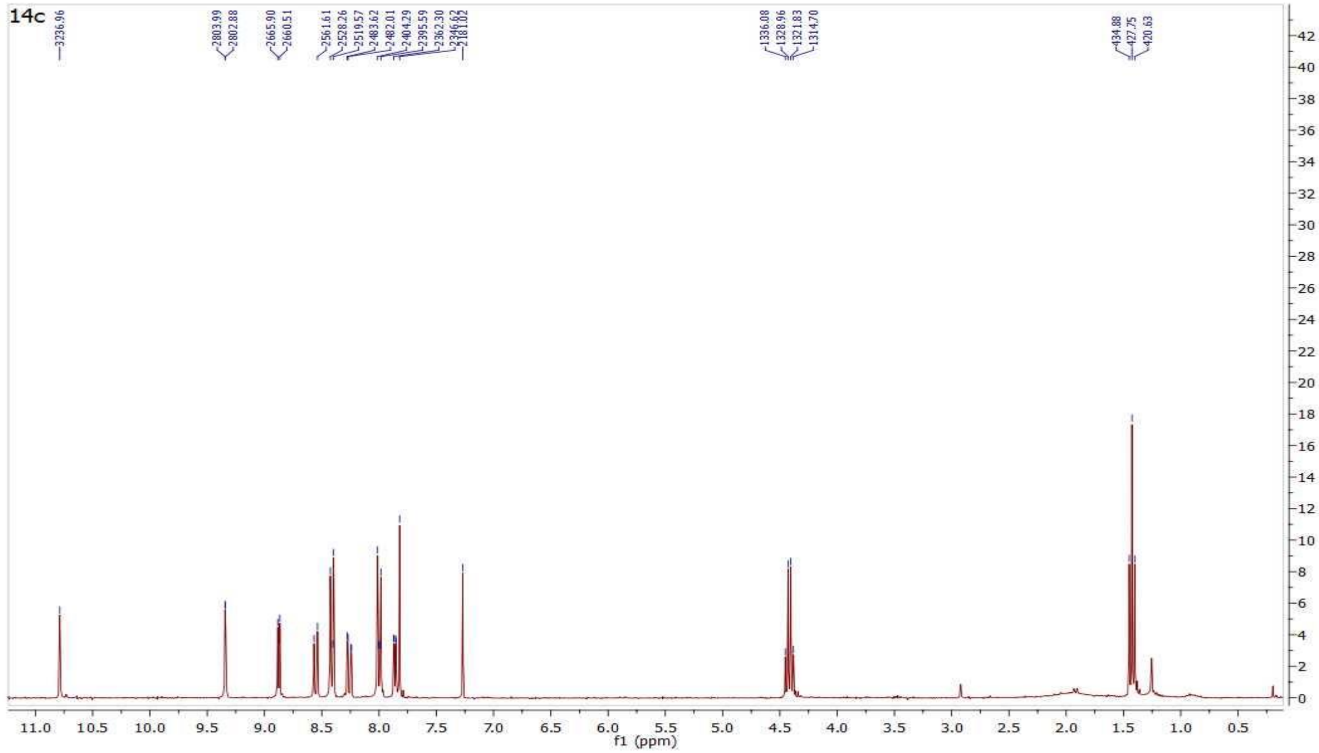
14c
3236.96
2803.99
2802.88
2665.90
2660.51
2561.61
2528.26
2519.57
2483.62
2482.01
2404.29
2395.59
2362.30
2346.62
2181.02
1336.08
1328.96
1321.83
1314.70
434.88
427.75
420.63
f1 (ppm)

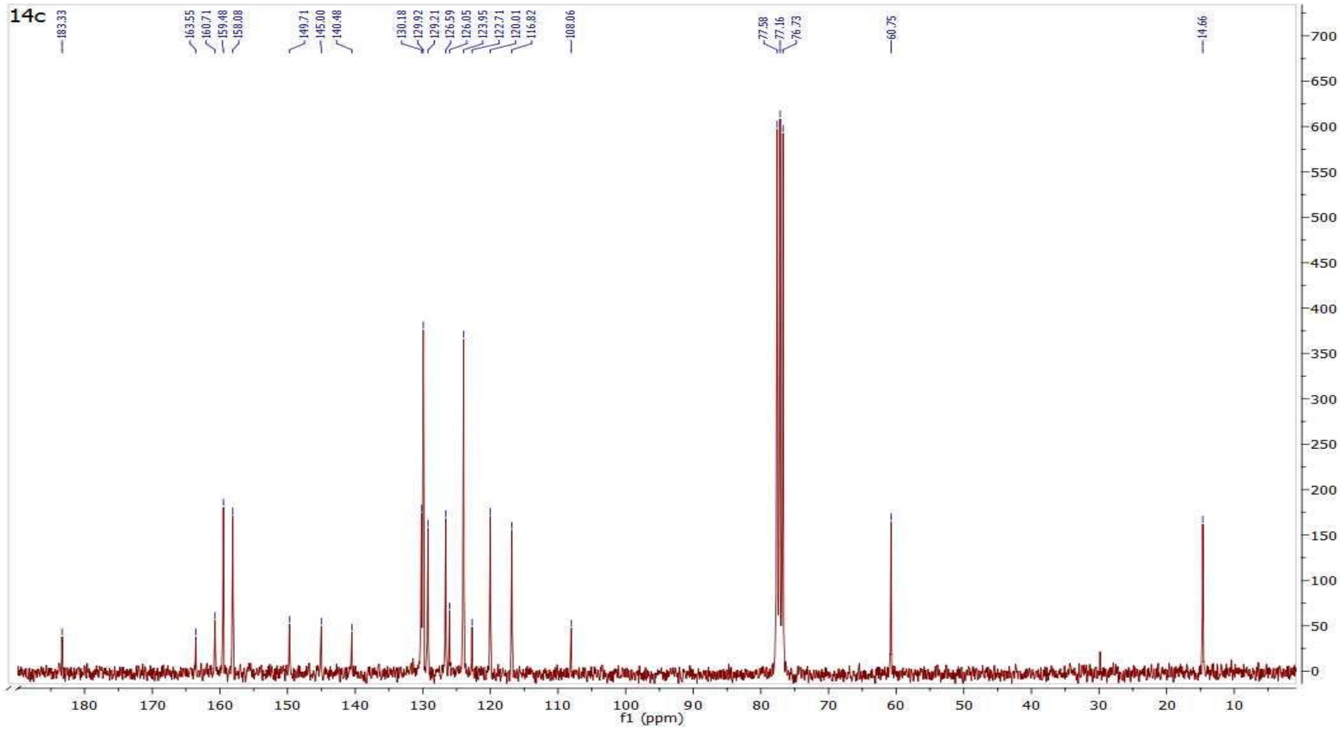
14c
183.33
163.55
160.71
159.48
158.08
149.71
145.00
140.48
130.18
129.92
129.21
126.59
126.05
123.95
122.71
120.01
116.82
108.06
77.58
77.16
76.73
60.75
14.66
f1 (ppm)

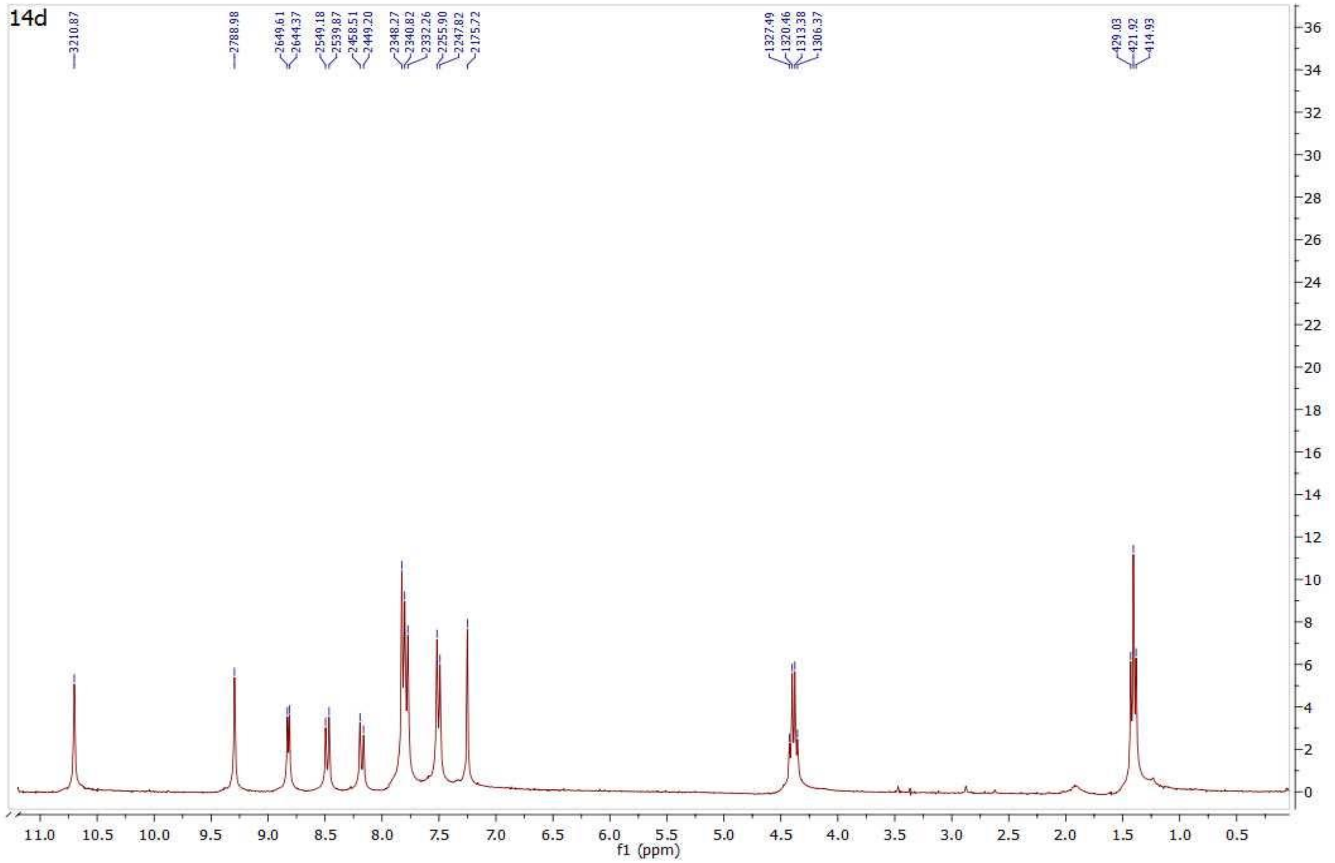
14d
3210.87
2788.98
2649.61
2644.37
2549.18
2539.87
2458.51
2449.20
2348.27
2340.82
2332.26
2255.90
2247.82
2175.72
1327.49
1320.46
1313.38
1306.37
429.03
421.92
414.93
f1 (ppm)

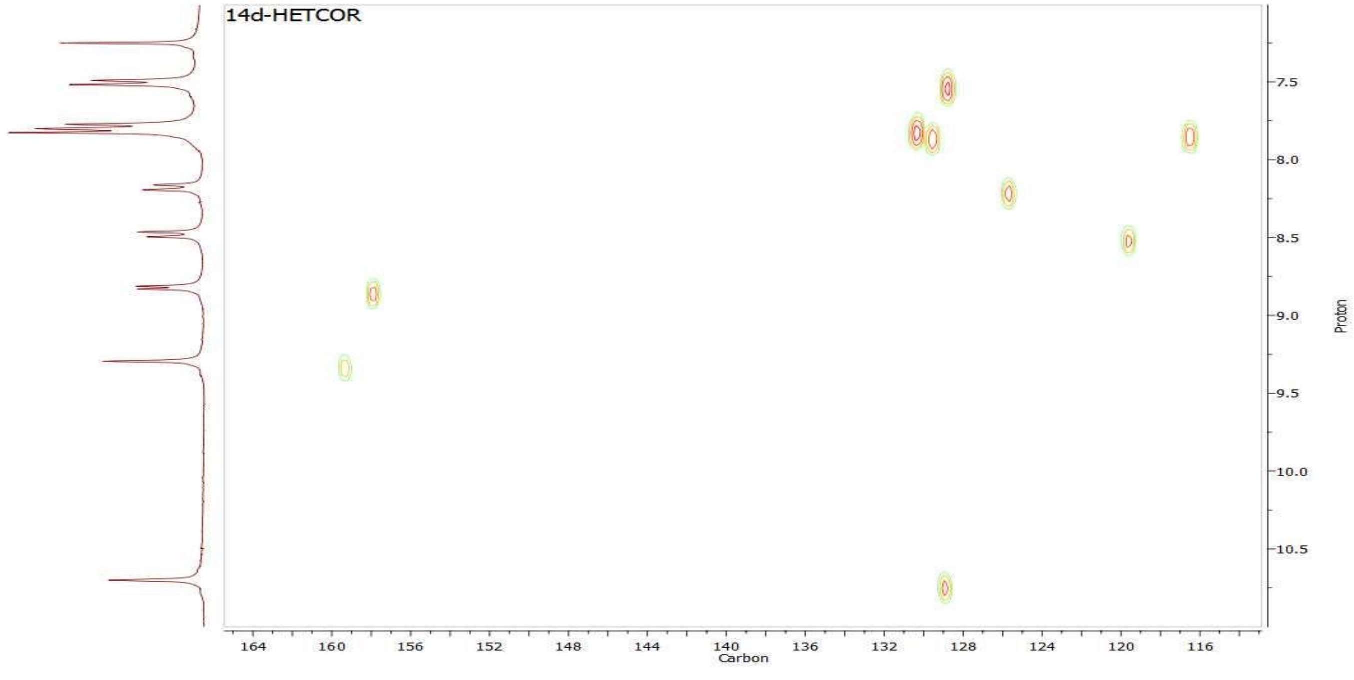
14d-HETCOR
Carbon
Proton

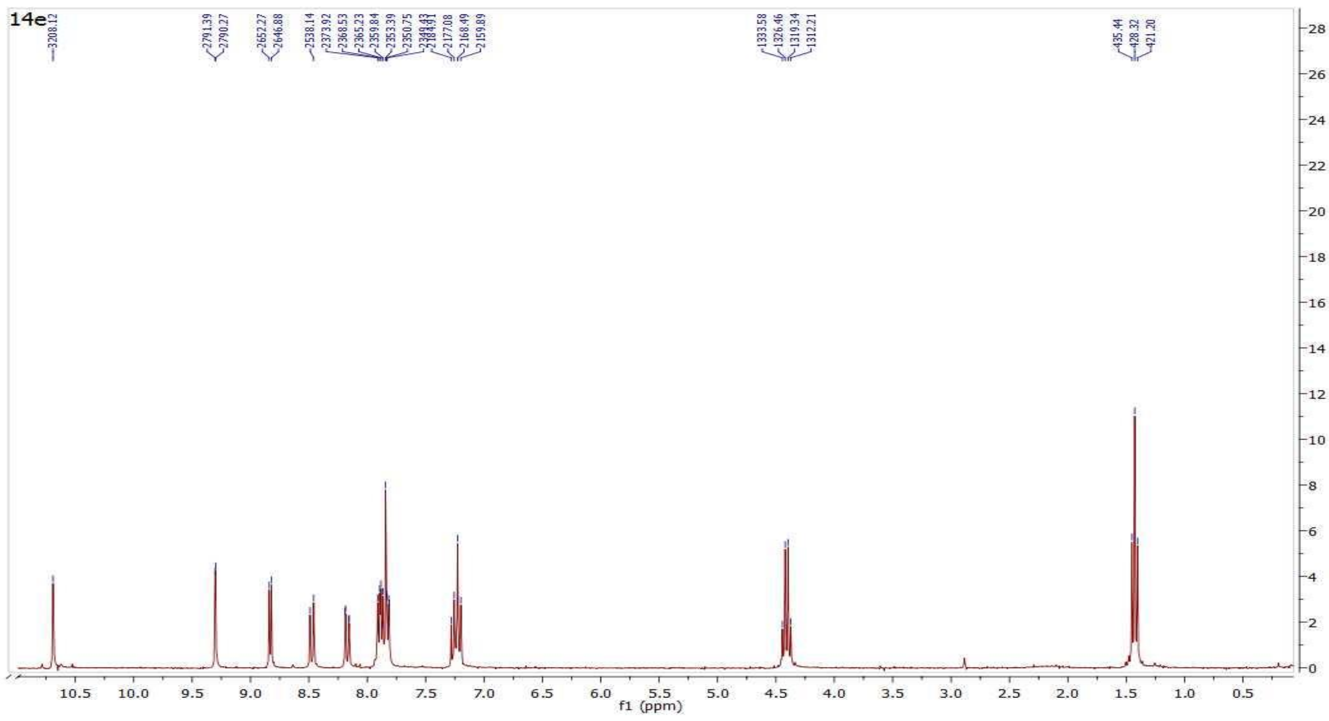
14e
3208.12
2791.39
2790.27
2652.27
2646.88
2538.14
2373.92
2368.53
2365.23
2359.84
2353.39
2350.75
2177.08
2168.49
2159.89
1333.58
1326.46
1319.34
1312.21
435.44
428.32
421.20
f1 (ppm)

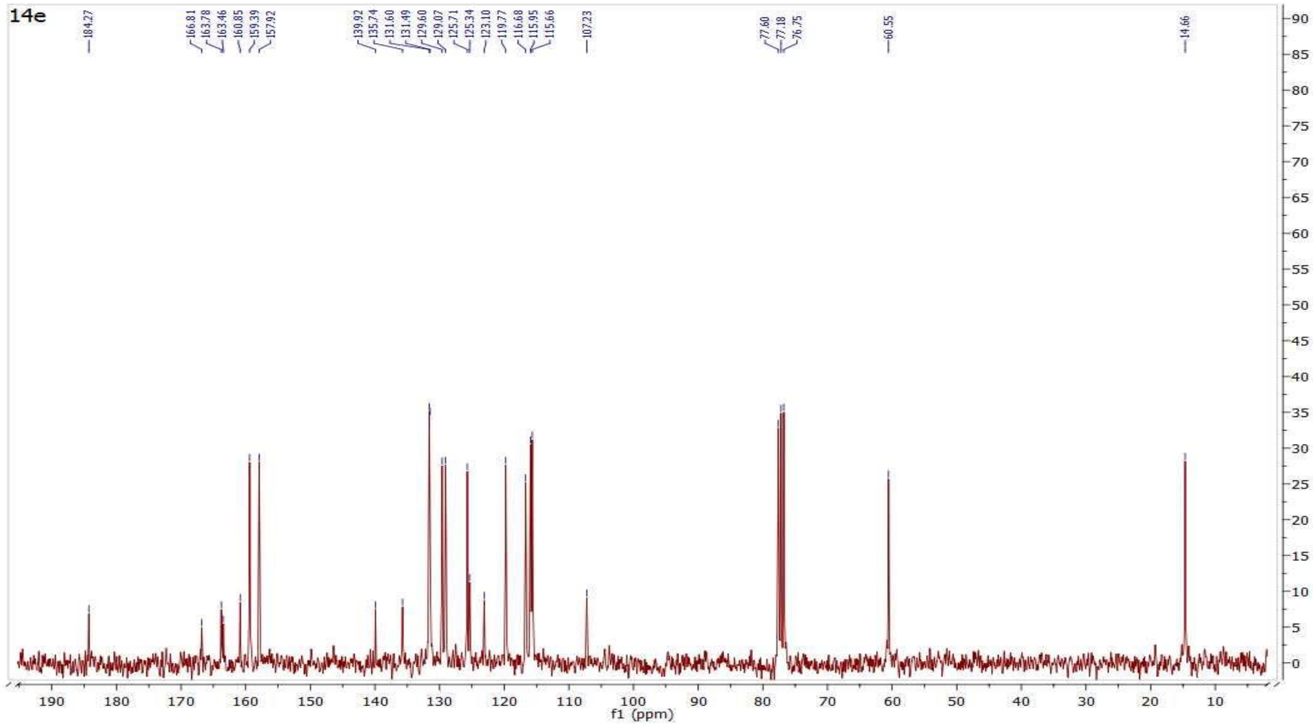
14e
184.27
166.81
163.78
163.46
160.85
159.39
157.92
139.92
135.74
131.60
131.49
129.60
129.07
125.71
125.34
123.10
119.77
116.68
115.95
115.66
107.23
77.60
77.18
76.75
60.55
14.66
f1 (ppm)

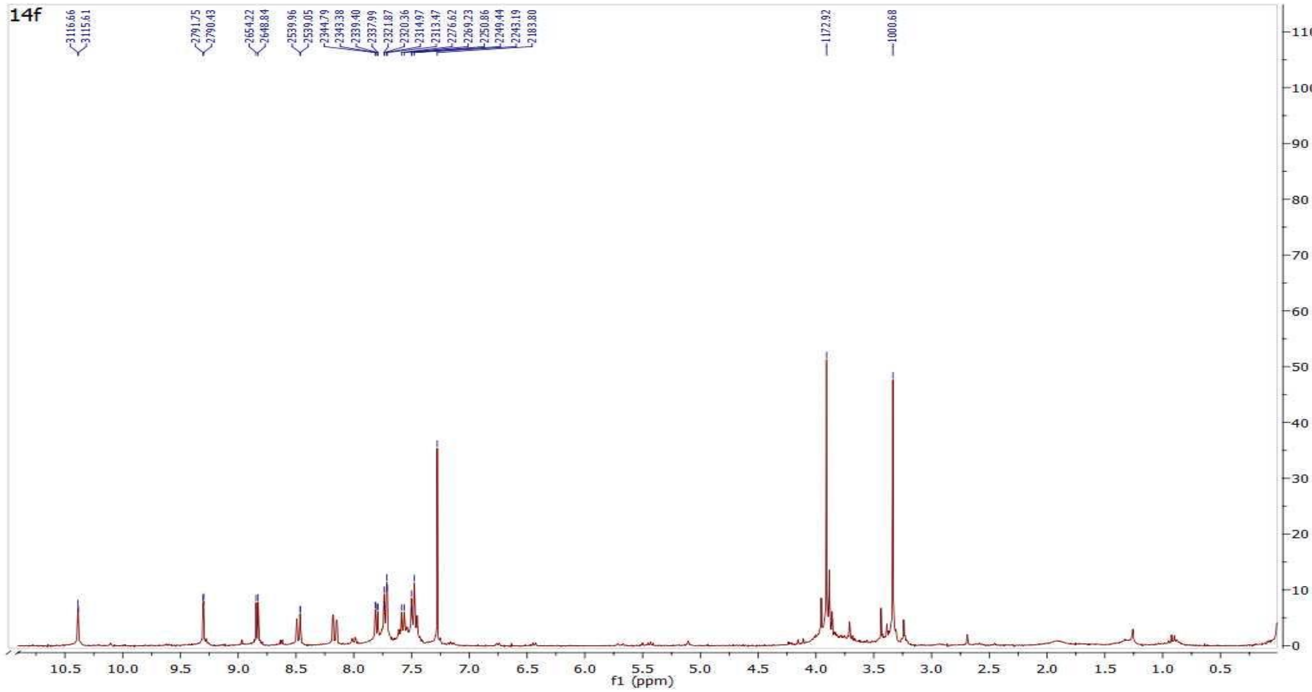
14f
3116.66
3115.61
2791.75
2790.43
2654.22
2648.84
2539.96
2539.05
2344.79
2343.38
2339.40
2337.99
2321.87
2320.36
2314.97
2313.47
2276.62
2269.23
2250.86
2249.44
2243.19
2183.80
1172.92
1000.68
f1 (ppm)

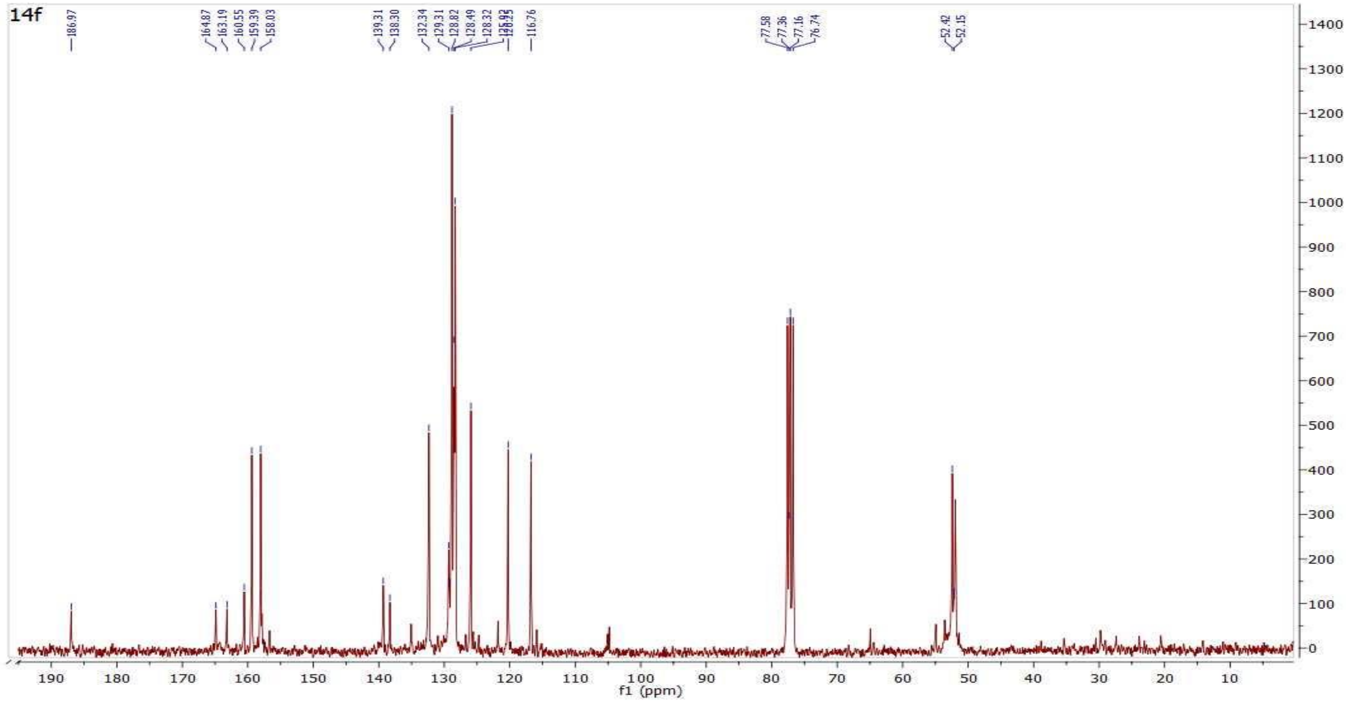
14f
186.97
164.87
163.19
160.55
159.39
158.03
139.31
138.30
132.34
129.31
128.82
128.49
128.32
116.76
77.58
77.36
77.16
76.74
52.42
52.15
f1 (ppm)

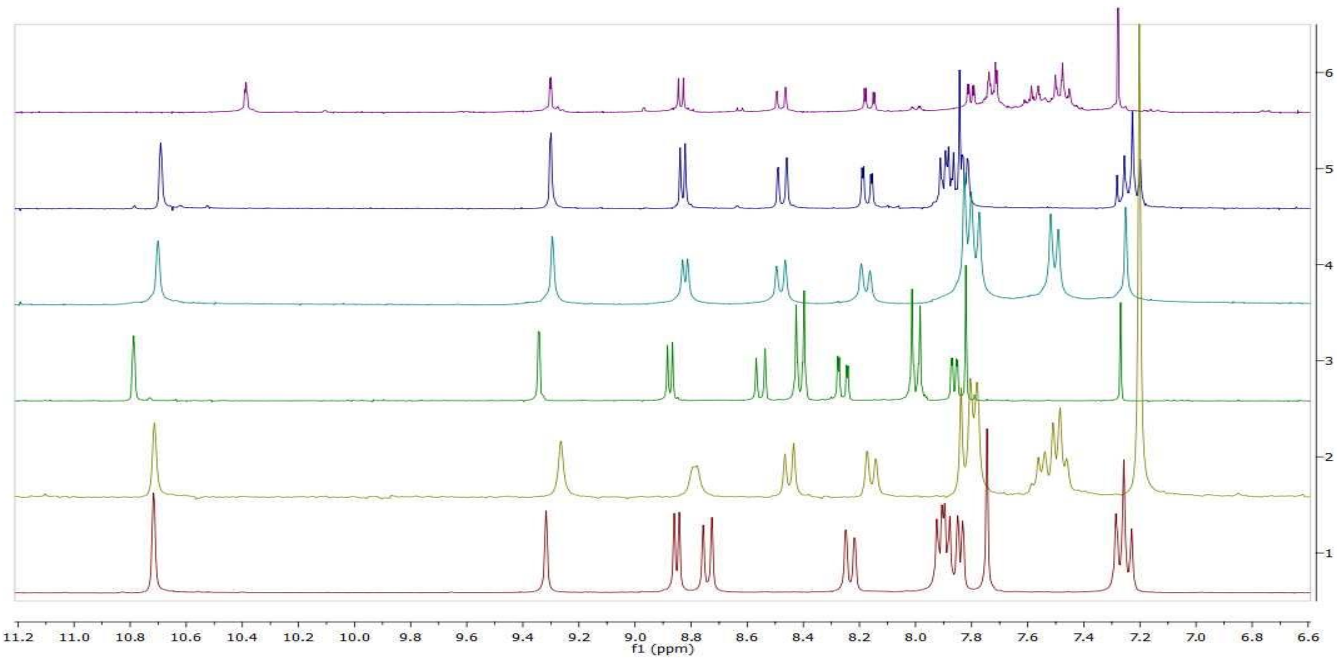

(Superimposed ^{1}H-NMR spectra of compounds **14-f** -aromatic region)

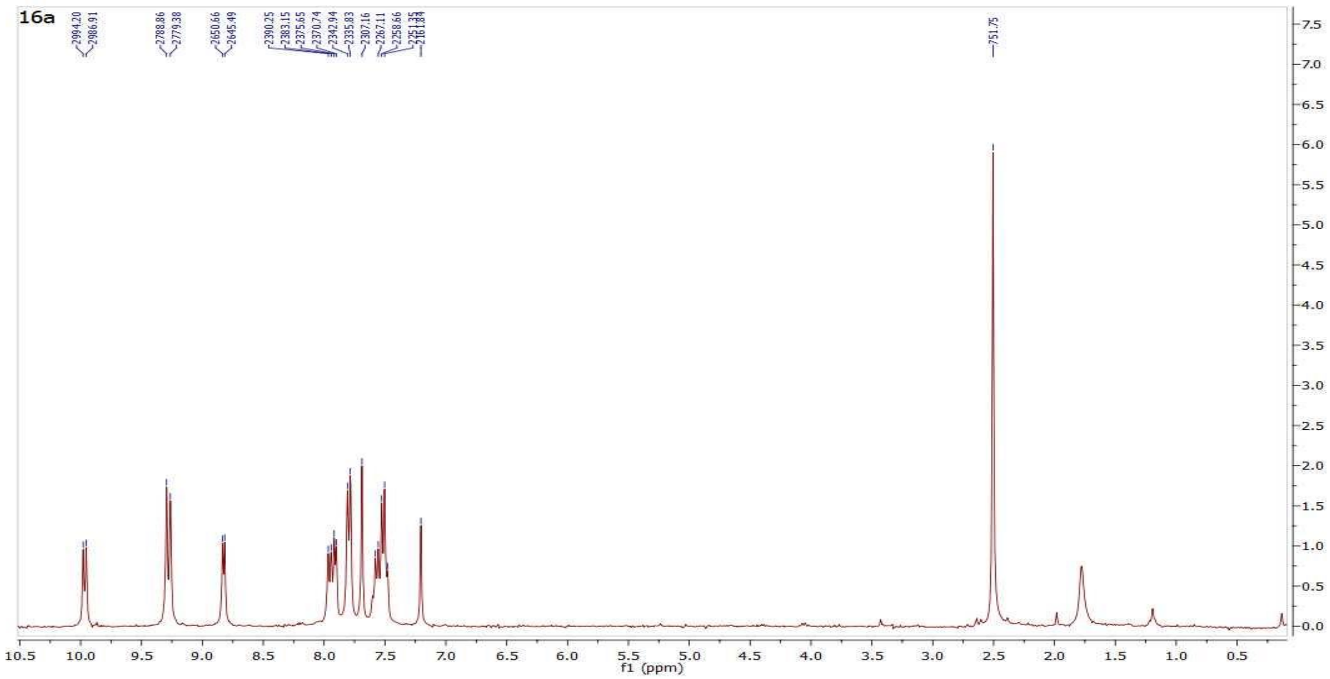

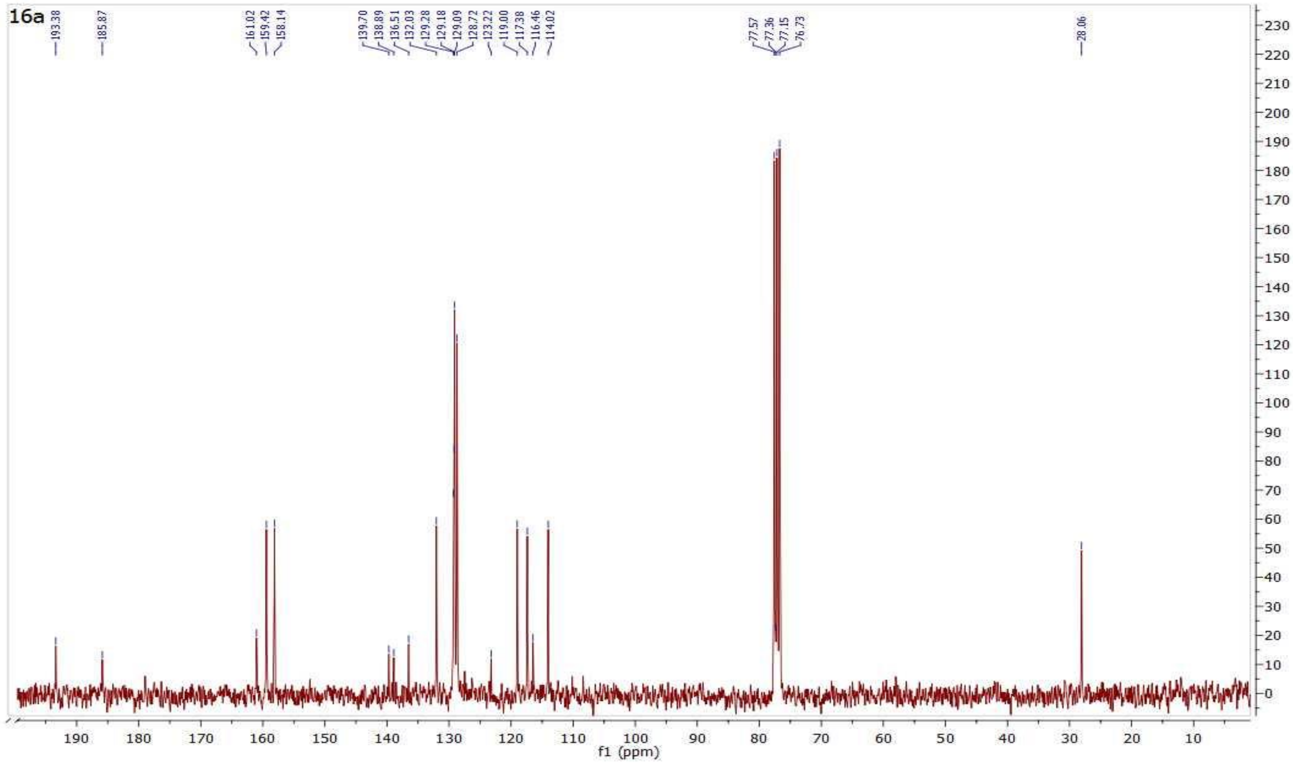
16a
193.38
185.87
161.02
159.42
158.14
139.70
138.89
136.51
132.03
129.28
129.18
129.09
128.72
123.22
119.00
117.38
116.46
114.02
77.57
77.36
77.15
76.73
28.06
f1 (ppm)

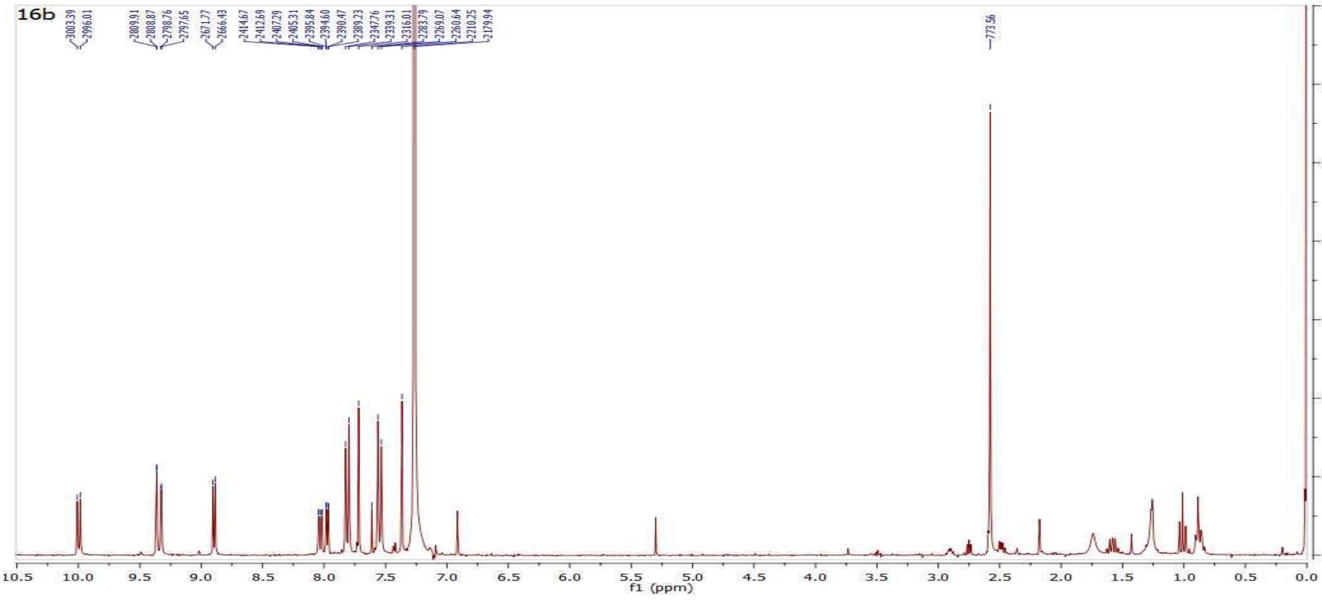
16b
f1 (ppm)

16b

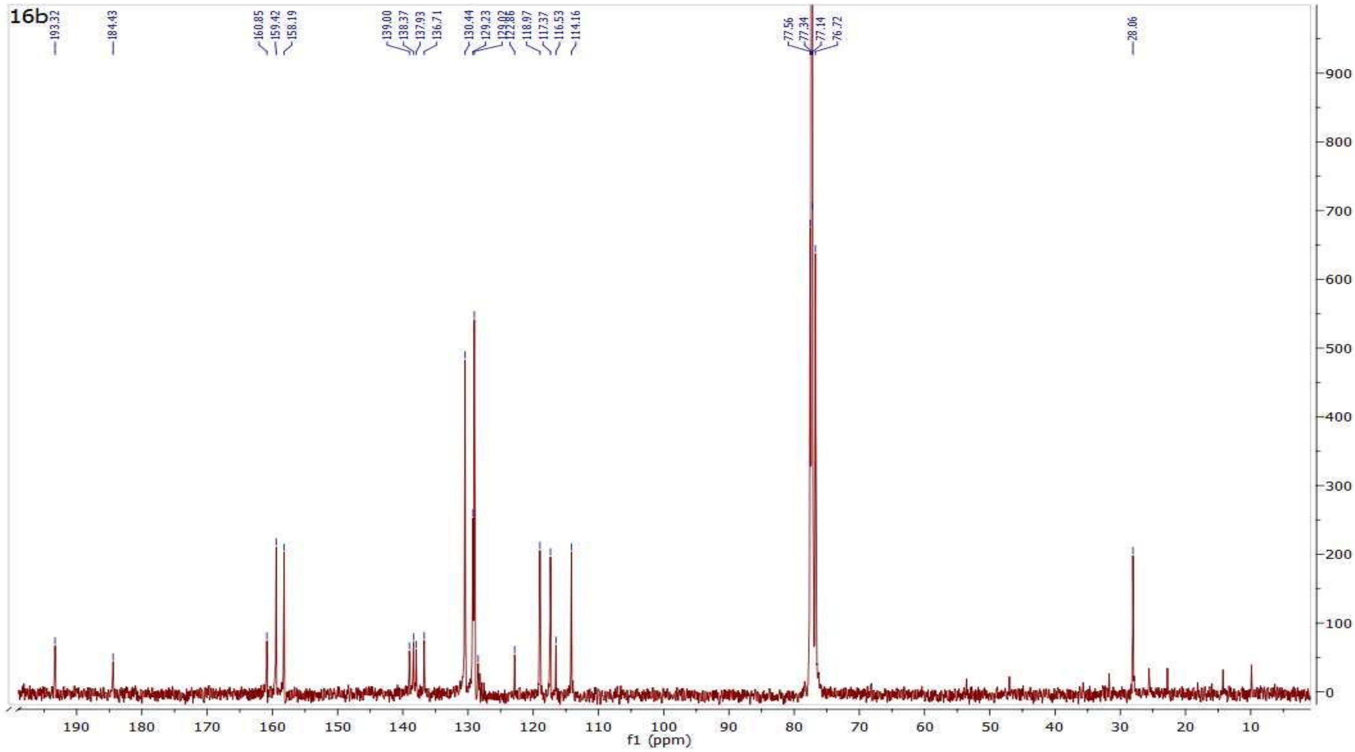
193.32
184.43
160.85
159.42
158.19
139.00
138.37
137.93
136.71
130.44
129.23
129.02
128.86
118.97
117.37
116.53
114.16
77.56
77.34
77.14
76.72
28.06
f1 (ppm)

16c

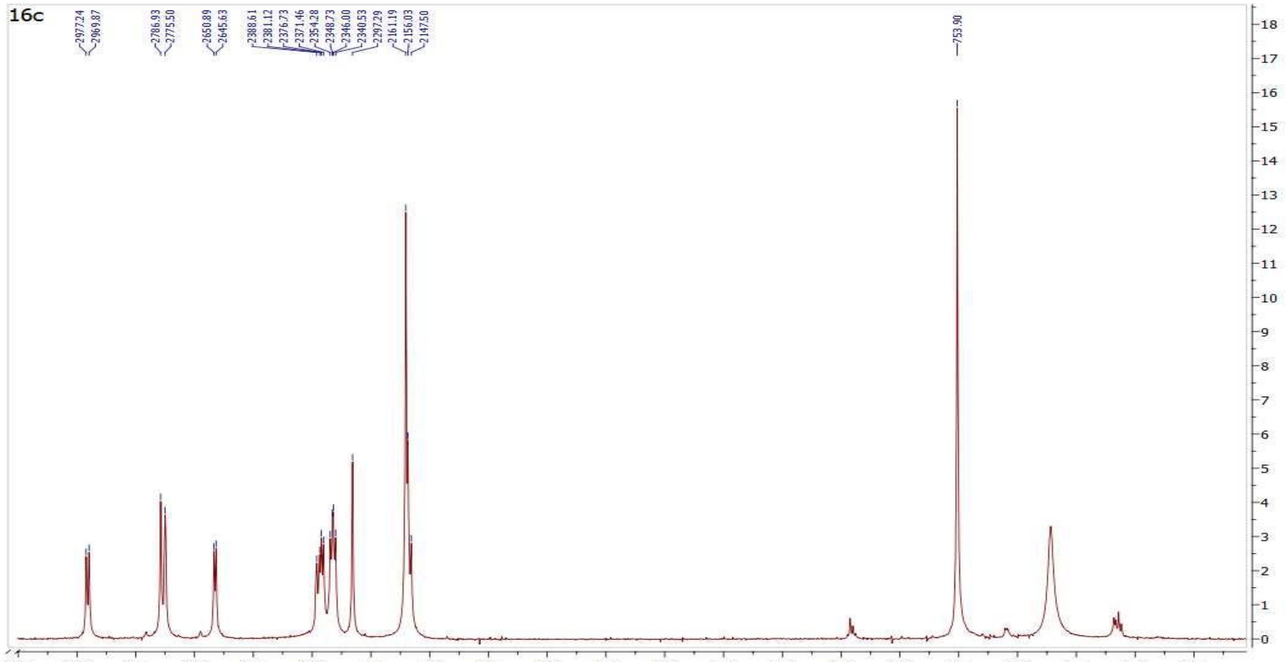
2977.24
2969.87
2786.93
2775.50
2650.89
2645.63
2388.61
2381.12
2376.73
2371.46
2354.28
2348.73
2346.00
2340.53
2297.29
2161.19
2156.03
2147.50
753.90
f1 (ppm)

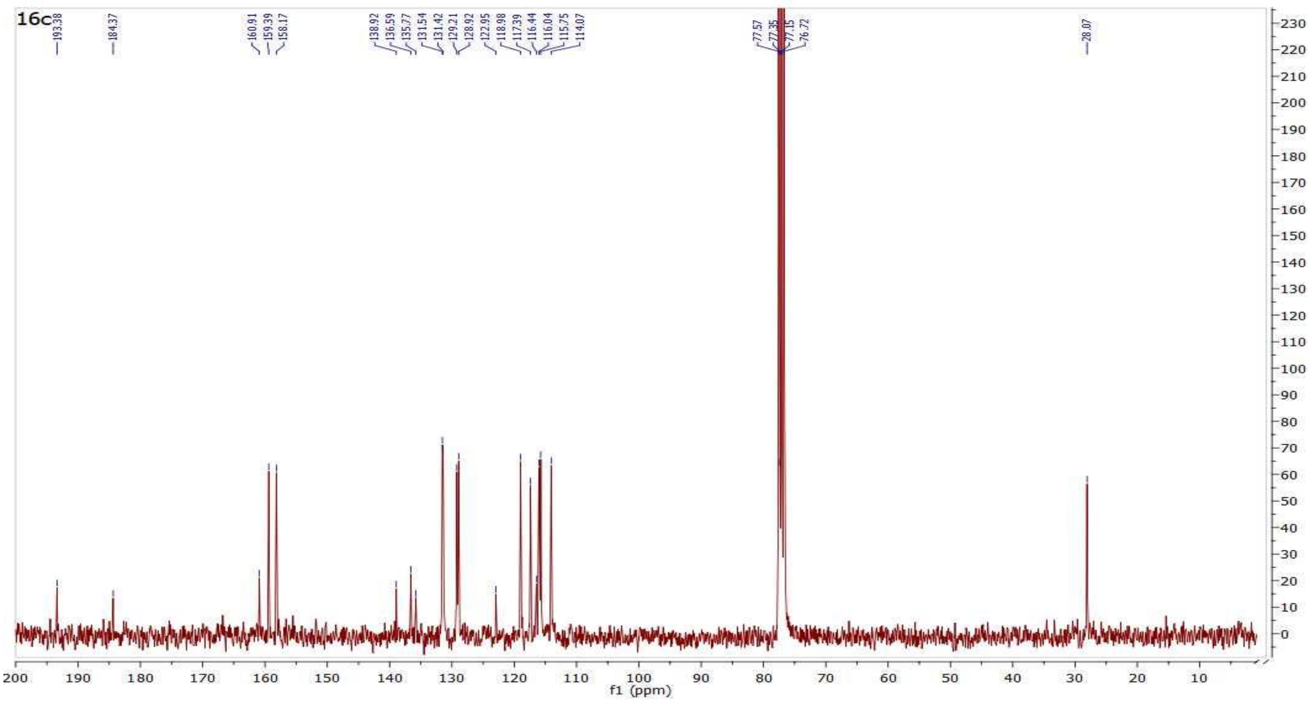
16c
f1 (ppm)

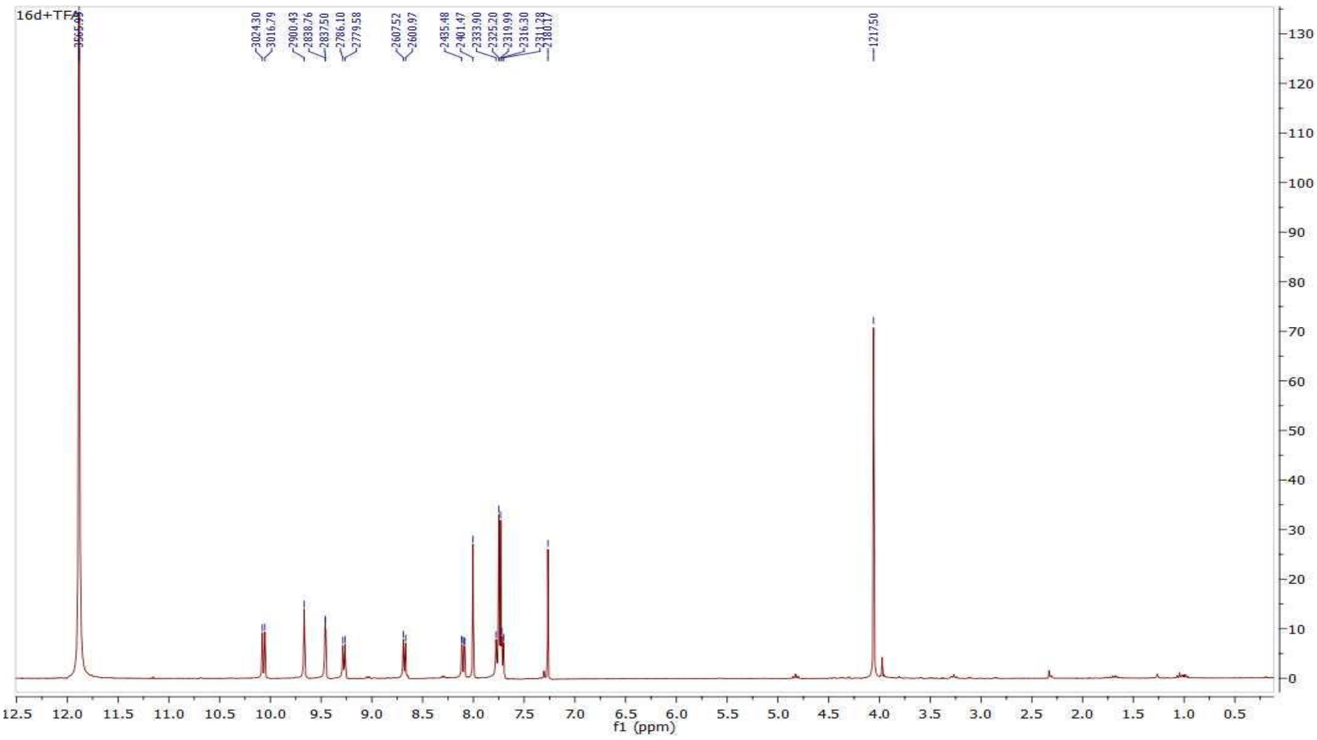
16d+TFA
f1 (ppm)

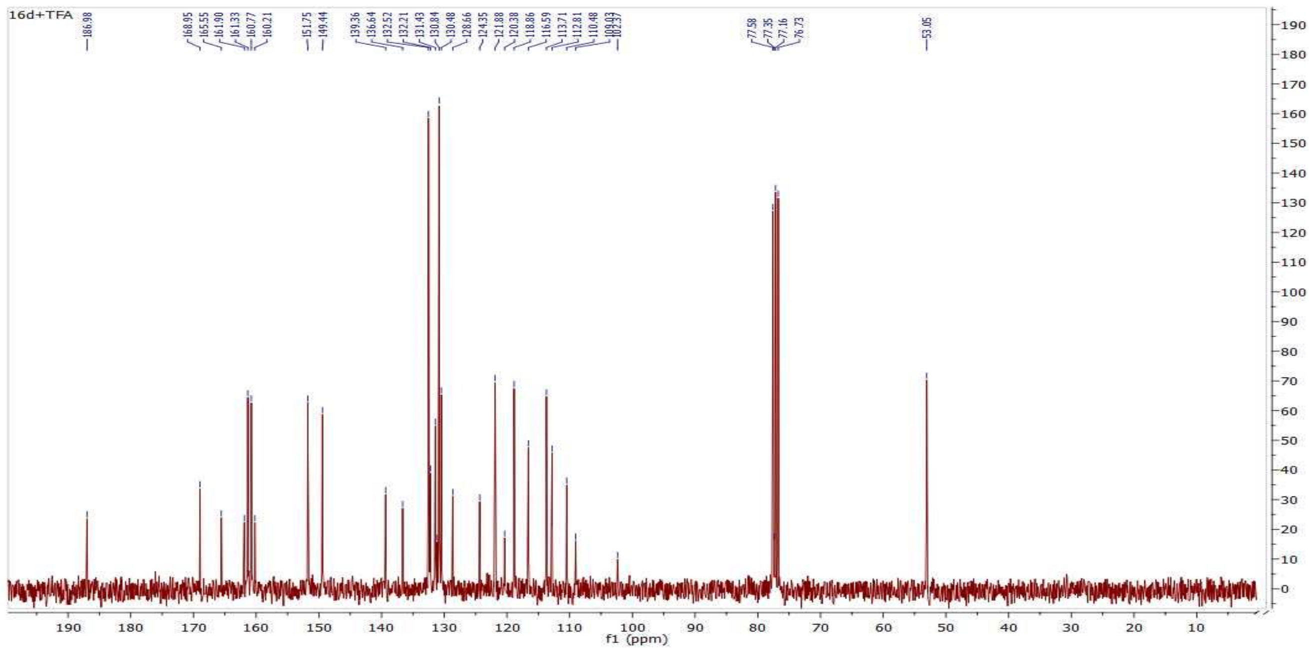
16d+TFA
186.98
168.95
165.55
161.90
161.33
160.77
160.21
151.75
149.44
139.36
136.64
132.52
132.21
131.43
130.84
130.48
128.66
124.35
121.88
120.38
118.86
116.59
113.71
112.81
110.48
109.03
102.57
77.58
77.35
77.16
76.73
53.05
f1 (ppm)

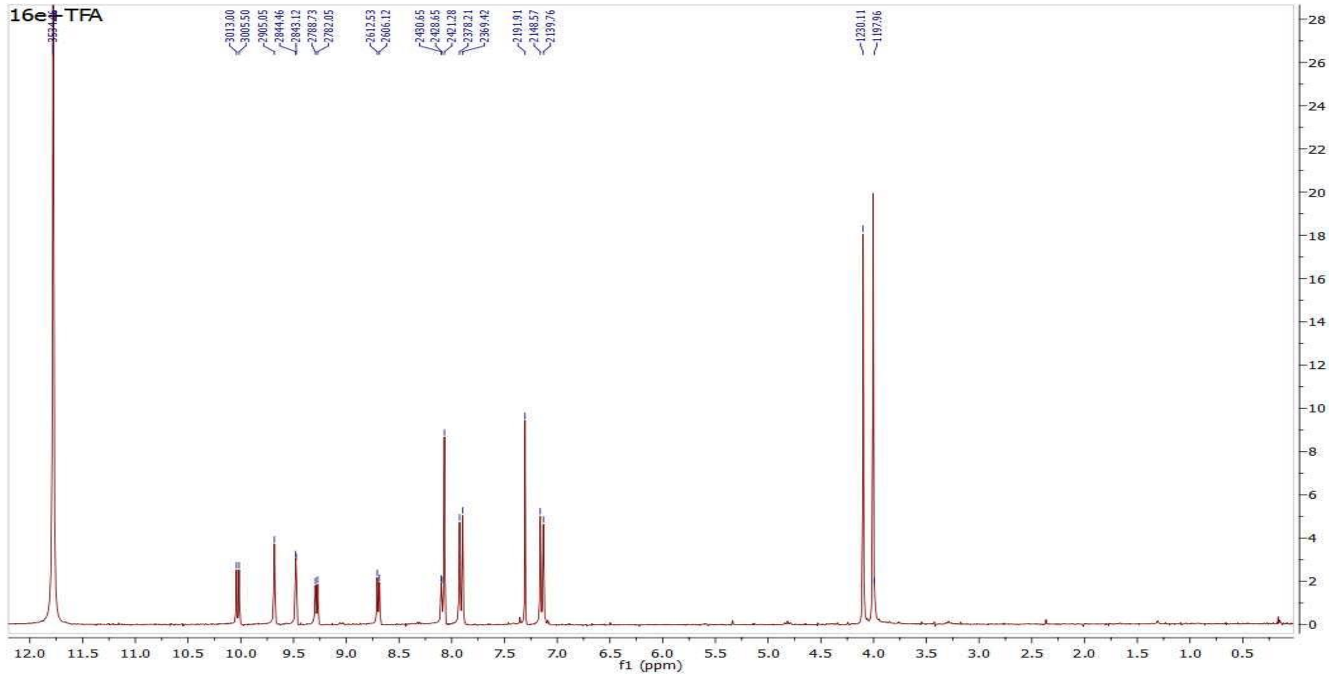
16e+TFA
3013.00
3005.50
2905.05
2844.46
2843.12
2788.73
2782.05
2612.53
2606.12
2430.65
2428.65
2421.28
2378.21
2369.42
2191.91
2148.57
2139.76
1230.11
1197.96
f1 (ppm)

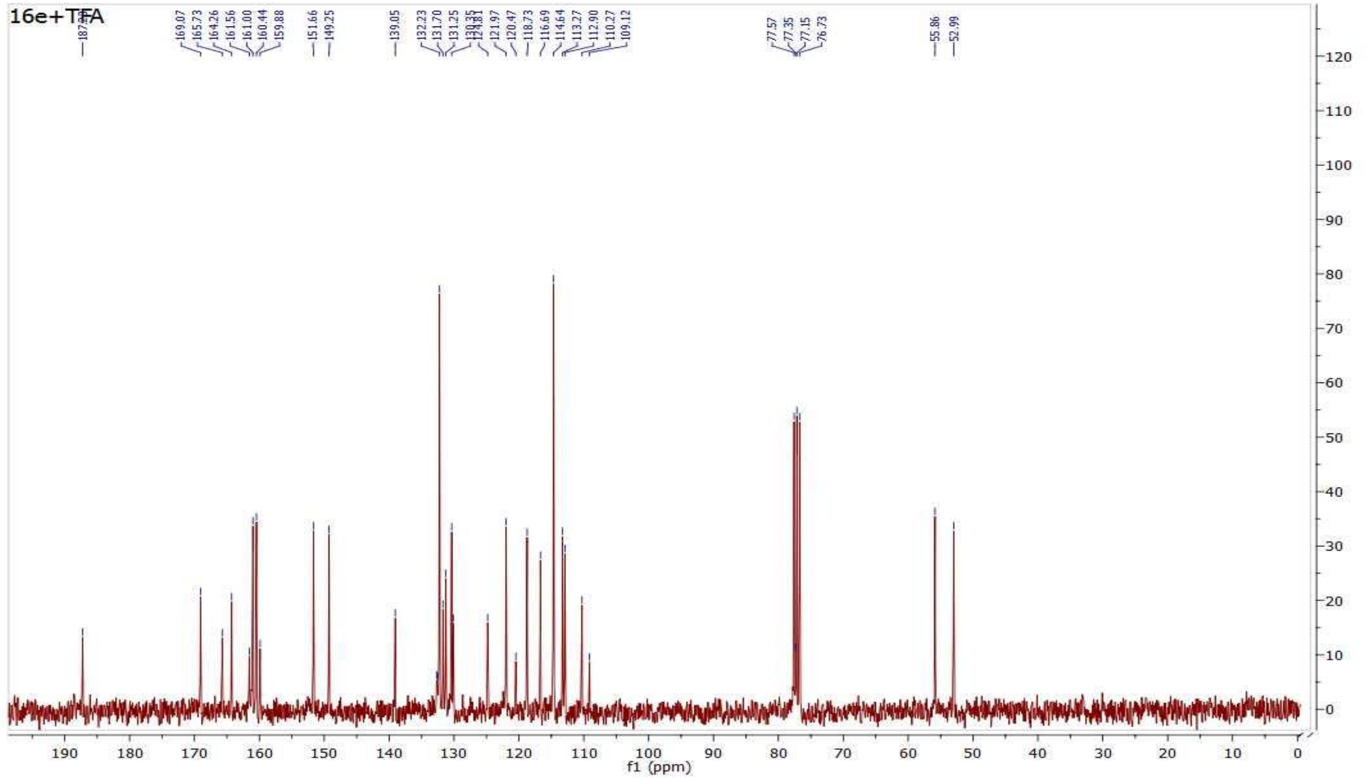
16e+TFA
f1 (ppm)

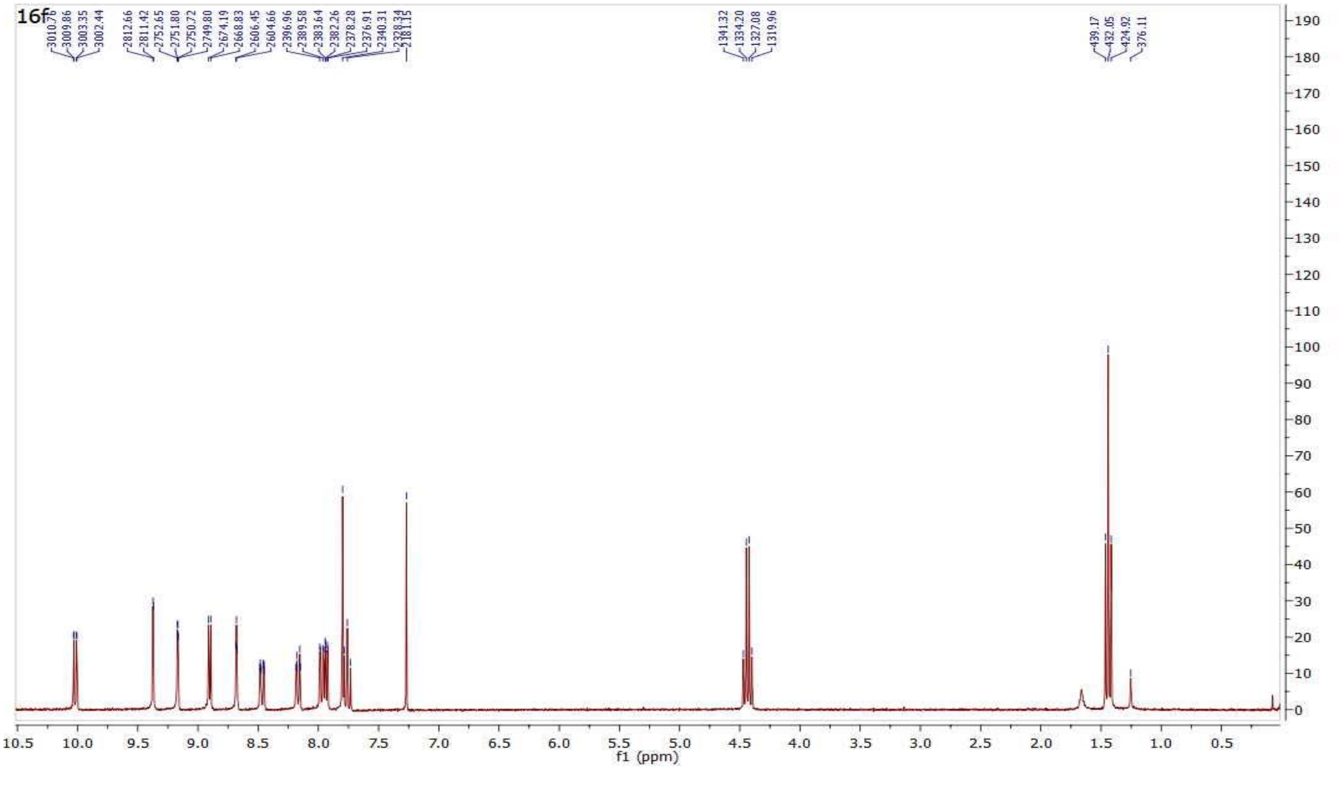
16f
f1 (ppm)

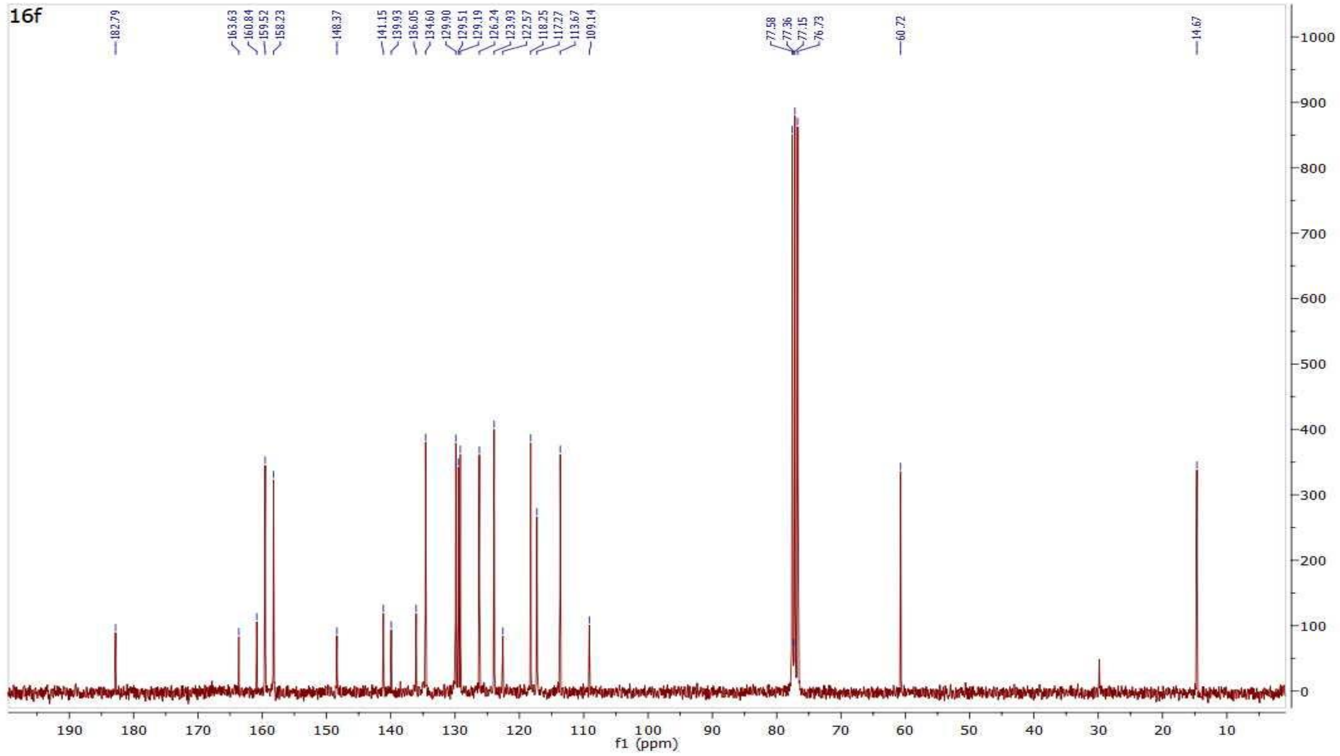

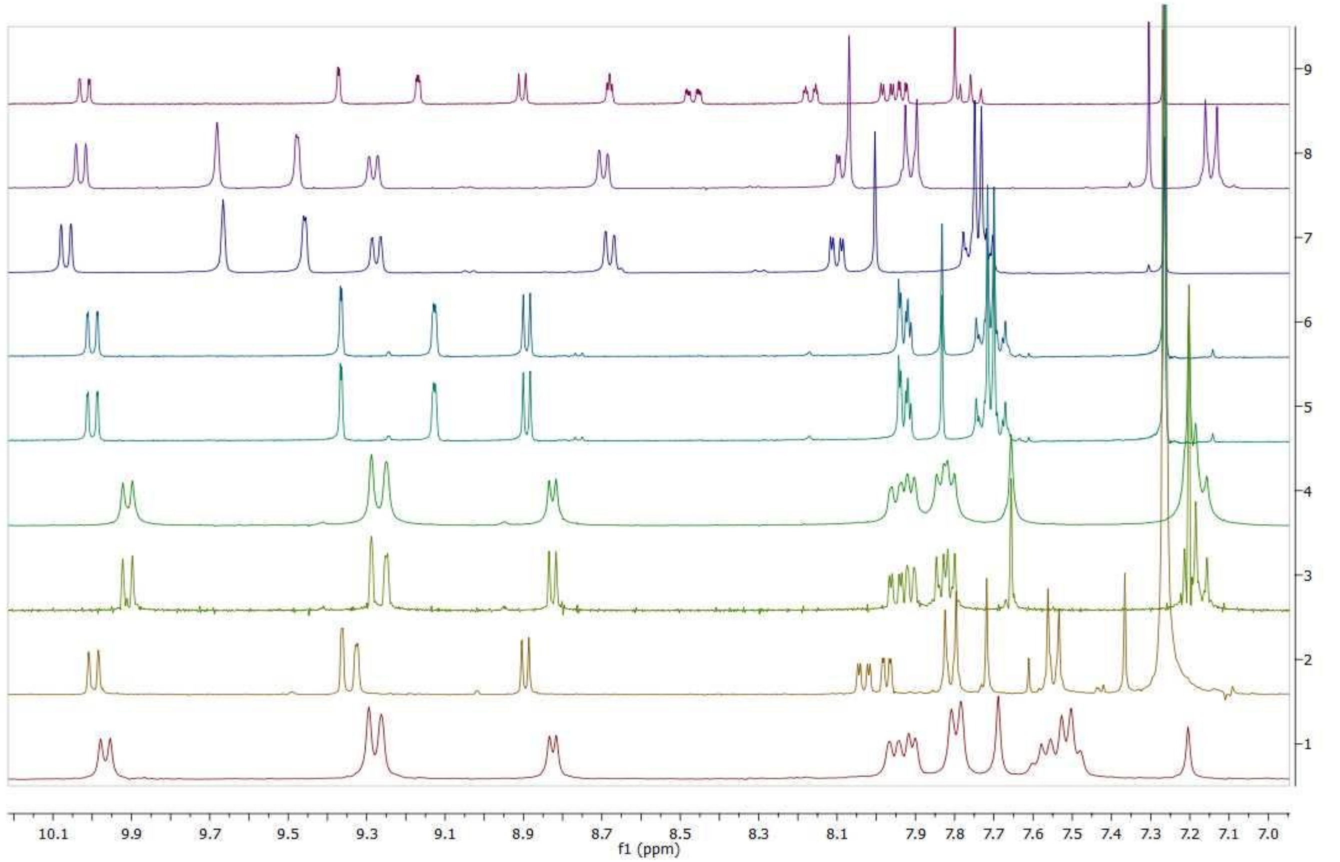

(Superimposed ^{1}H-NMR spectra of compounds **16a-f**-aromatic region)
